# Hydrogen Drives Part of the Reverse Krebs Cycle under Metal or Meteorite Catalysis

**DOI:** 10.1002/anie.202212932

**Published:** 2022-11-22

**Authors:** Sophia A. Rauscher, Joseph Moran

**Affiliations:** ^1^ Institut de Science et d'Ingénierie Supramoléculaires (ISIS) CNRS UMR 7006 University of Strasbourg 8 Allée Gaspard Monge 67000 Strasbourg France; ^2^ Institut Universitaire de France (IUF) France

**Keywords:** Hydrogenation, Metabolism, Prebiotic Chemistry, Reduction, Reverse Krebs Cycle

## Abstract

Hydrogen (H_2_) is a geological source of reducing electrons that is thought to have powered the metabolism of the last universal common ancestor to all extant life, and that is still metabolized by various modern organisms. It has been suggested that H_2_ drove a geochemical analogue of some or all of the reverse Krebs cycle at the emergence of the metabolic network, catalyzed by metals, but this has yet to be demonstrated experimentally. Herein, we show that three consecutive steps of the reverse Krebs cycle, converting oxaloacetate into succinate, can be driven without enzymes and in one‐pot by H_2_ as the reducing agent under mild conditions compatible with biological chemistry. Low catalytic amounts of nickel (10–20 mol %) or platinum group metals (0.1–1 mol %) or even small amounts of ground meteorites were found to promote the reductive chemistry at temperatures between 5 and 60 °C and over a wide pH range, including pH 7. These results lend additional support to the hypothesis that geologically produced hydrogen and metal catalysts could have initiated early metabolic networks.

## Graphical abstract

Hydrogen is a geological reducing agent that powers the metabolisms of modern organisms and potentially also powered the metabolism of the last universal common ancestor to all extant life. It is shown that catalytic amounts of transition metals or small amounts of powdered meteorites allow hydrogen to drive a key sequence of the reverse Krebs cycle under mild aqueous non‐enzymatic conditions potentially relevant to the emergence of metabolic networks.

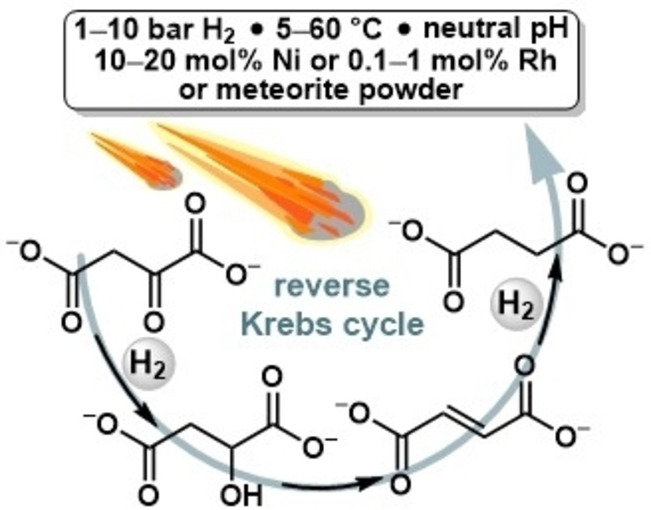

Metabolic networks are fundamental to biochemistry. Therefore, some metabolic theories of the origin of life revolve around the idea that metabolism emerged first from self‐organized reaction networks in the early stages of the emergence of life.[^1^, ^2^, ^3^, ^4^, ^5^, ^6^, ^7^] In the absence of enzymes, it is has been proposed that metals or minerals could have promoted the formation of metabolites, including genetic molecules, some of which could have catalyzed their own formation or enabled new reactivity. In this way, the metabolic network is suggested to have expanded over time, eventually coming to resemble modern core metabolic pathways. The organic molecules produced by the reaction network are thought to have helped replace the original metal catalysts through kinetic competition, eventually leading to the enzymes, metalloenzymes, and cofactors that catalyze many of the reactions of core metabolism today.^[8]^

A requirement for (proto‐)metabolic networks, today as then, is a constant flow of energy to enable and sustain chemical processes.^[9]^ Several arguments support H_2_ as a likely driver of proto‐metabolic networks. From a biological perspective, multiple lines of evidence suggest that H_2_ drove the metabolism of the last universal common ancestor to all living organisms.[^10^, ^11^, ^12^, ^13^] H_2_ is still widely used in metabolism by some modern bacteria, archaea, and lower eukaryotes. Many of these organisms harness H_2_ using [FeFe]‐ or [NiFe]‐ hydrogenase enzymes.[^14^, ^15^] From a geological perspective, H_2_ is continuously generated by two ubiquitous processes in the Earth's crust. The first process, serpentinization, involves the H_2_‐releasing reaction of water with Fe^2+^ in rocks.[^16^, ^17^] The second process, water radiolysis, involves the splitting of water into H_2_ and reactive oxygen species through the decay of naturally occurring radioactive elements.[^18^, ^19^, ^20^] These two processes are collectively estimated to have generated roughly 10^11^ mol of H_2_ per year during the pre‐Cambrian era,^[21]^ much of which is naturally funneled into specific locations, like hydrothermal vents, or underground reservoirs. From a chemical perspective, H_2_ is well known to be reactive in the presence of various metallic catalysts. Despite these strong arguments, we have surprisingly little experimental evidence on whether and how metallic catalysts might have allowed H_2_ to drive protometabolism.

The extent to which prebiotic chemistry resembled metabolism is highly debated.[^22^, ^23^] However, it is parsimonious to expect continuity, and thus some similarity between them.[^24^, ^25^, ^26^] Recently, efforts have been made to recreate ancient metabolic pathways under non‐enzymatic conditions.[^27^, ^28^, ^29^, ^30^, ^31^, ^32^, ^33^, ^34^] Among biological anabolic pathways, attention has largely focused on two interconnected CO_2_‐fixing pathways, the Acetyl‐CoA (AcCoA) pathway and the reverse Krebs cycle (Figure 1), both of which have H_2_‐dependent variants. A non‐enzymatic H_2_‐dependent variant of the AcCoA pathway, which converts CO_2_ and H_2_ into formate, acetate, and pyruvate, was found to be promoted by Fe or Ni minerals in 2020.^[27]^ Non‐enzymatic analogs of the reverse Krebs cycle have also been investigated, but so far, none of them have been found that depend on H_2._ The reverse Krebs cycle is of great biochemical significance because five of its eleven intermediates are the starting points for the biosynthesis of sugars, amino acids, lipids, cofactors, and pyrimidine nucleotides. In addition to this central biosynthetic role, the reverse Krebs cycle is an autocatalytic cycle. This allows it to concentrate matter and energy into the critical intermediates for life.^[35]^ However, only parts of the reverse Krebs cycle operate in most of the organisms that use it.[^36^, ^37^] For this reason, partial versions of the pathway that are not autocatalytic have also been proposed to be primordial^[1]^, and it might not be necessary to find a complete prebiotic version of the pathway. Most attempts to uncover a partial non‐enzymatic analog of the reverse Krebs cycle have focused on two of the reduction reactions in the first half of the cycle (Figure 1): the reduction of oxaloacetate to malate (reaction 3) and of fumarate to succinate (reaction 5). The action of UV photochemistry of sulfide on semiconducting ZnS colloids was shown to promote reactions 3 and 5.^[38]^ Electroreduction on metal sulfides^[39]^ or cyanide^[40]^ were shown to promote both reactions. In an attempt to create a coherent reaction network, rather than focusing on individual reactions, it was shown that reactions 3—5 could be carried out in a continuous manner in the same pot using metallic iron as a reducing agent together with stoichiometric Zn^2+^ as a promoter for the dehydration of malate under highly acidic (1 M HCl) conditions at high temperatures (70—140 °C).^[41]^ However, these harsh conditions are likely of no direct relevance to the origin of the metabolic network. Ideally, a partial and non‐enzymatic version of the reverse Krebs cycle would be compatible with the conditions of biological chemistry, geologically viable within a localized environment, and aligned with ancient biochemistry from the standpoint of its inputs (i.e., driven by H_2_). We, therefore, set out to identify mild conditions where a partial reverse Krebs cycle could be driven by H_2_ using metal catalysis.

**Figure 1:**
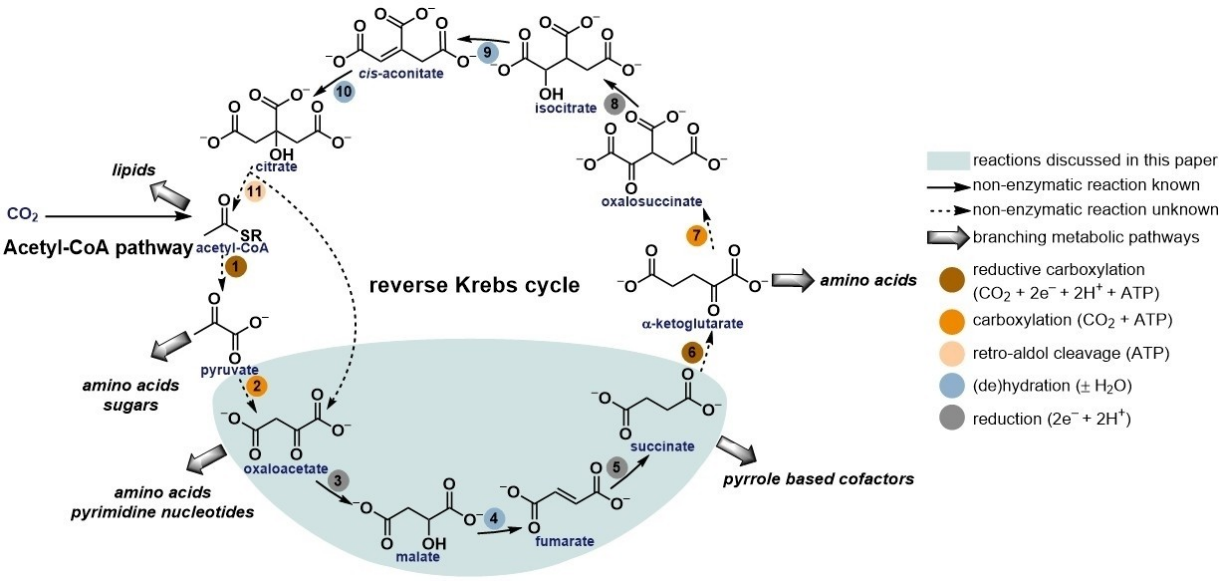
Reactions of the reverse Krebs cycle.

To identify inorganic catalysts that could affect H_2_‐dependent variants of the reduction reactions of the reverse Krebs cycle, we elected to screen a wide variety of simple inorganic catalysts, including metals known to be hydrogenation catalysts.^[42]^ Homogeneous and heterogeneous forms of earth‐abundant metals found in biology were chosen, such as Fe, Ni, and Co, but also rarer platinum group metals, such as Ru, Rh, Pd, Ir, and Pt. The first reaction assayed was the reduction of fumarate to succinate (reaction 5 in Figure 1), which is an alkene hydrogenation. We selected rather mild conditions for the screening protocol: room temperature, neutral unbuffered water (pH adjusted manually before the reaction), and roughly 1 atm H_2_ using an H_2_ balloon. After 18 h, the yields were determined by quantitative ^1^H NMR spectroscopy by integrating against dimethyl sulfone (DMS) as internal standard. Among the catalysts assayed under these conditions (Table 1 and Table S1 in Supporting Information for the complete screen), succinate was obtained almost quantitatively using 1 mol % of RhCl_3_, PdCl_2_, or Rh/Al_2_O_3_ (entries 1, 3 and 4). Under the same conditions, PtCl_2_ and Pt/Al_2_O_2_ reduced fumarate to succinate in yields of 76 % and 91 %, respectively (entries 2 and 5). For Ni/SiO_2_−Al_2_O_3_, succinate was obtained in 33 % yield at 1 mol % loading but was produced quantitatively when a higher catalyst loading of 10 mol % was used (entries 6—7). Thus, numerous metals in various oxidation states are able to serve as catalysts for fumarate hydrogenation under conditions compatible with biological chemistry.

**Table 1:** Catalyst screen for the reduction of fumarate (30 mM) to succinate.^[a]^

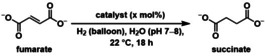			
Entry	Catalyst	Catalyst loading [mol %]	Yield [%]^[b]^
1	RhCl_3_	1	96.6±0.3
2	PtCl_2_	1	76.4±1.8
3	PdCl_2_	1	95.7±1.1
4	Rh/Al_2_O_3_	1	99.1±0.2
5	Pt/Al_2_O_3_	1	90.7±0.7
6	Ni/SiO_2_−Al_2_O_3_	1	32.7±3.4
7	Ni/SiO_2_−Al_2_O_3_	10	98.6±1.3

[a] Reaction conditions: fumarate (30 mM), catalyst, 1 mL H_2_O (pH 7–8), 22 °C, 1 atm H_2_ (balloon), 18 h. [b] Yields represent mean values of three experiments with associated standard errors and were determined by quantitative ^1^H NMR spectroscopy with dimethyl sulfone as an internal standard.

The second reaction assayed was the reduction of oxaloacetate to malate (reaction 3 in Figure 1), which is a ketone hydrogenation. The catalyst screening was performed under the same conditions used for the reduction of fumarate (Table 2, see Supporting Information Table S2 for complete screening). In general, oxaloacetate is unstable in water and prone to decarboxylate to pyruvate. This reaction is enhanced by the presence of metals or with increasing temperature.[^43^, ^44^] For this reason, pyruvate and its reduction product, lactate, are also observed in the reaction mixture. A catalyst loading of 1 mol % of RhCl_3_, PtCl_2_, or PdCl_2_ catalyzed the reduction of oxaloacetate to malate in yields between 2 and 6 % (entries 1—3), whereas 1 mol % Rh or Pt on alumina support gave malate in 92 % and 25 % yields, respectively (entries 4—5). For Ni/SiO_2_−Al_2_O_3_ (10 mol %), 23 % malate was obtained (entry 7). No reactivity was observed with catalytic amounts of Ir, Ru, Co, or Fe for either reduction reactions (see Supporting Information Table S1 and S2). As Rh/Al_2_O_3_ gave the highest reduction yields for both fumarate and oxaloacetate among the various platinum group metals tested, and since Ni/SiO_2_−Al_2_O_3_ was the only catalyst found to show the reactivity among more abundant coinage metals tested, we proceeded to study these two catalysts in further detail.

**Table 2:** Catalyst screen for the reduction of oxaloacetate (30 mM) to malate.^[a]^

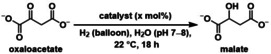			
Entry	Catalyst	Catalyst loading [mol %]	Yield [%]^[b]^
1	RhCl_3_	1	6.4±1.0
2	PtCl_2_	1	2.6±0.4
3	PdCl_2_	1	3.3±0.6
4	Rh/Al_2_O_3_	1	92.0±1.5
5	Pt/Al_2_O_3_	1	25.3±1.2
6	Ni/SiO_2_−Al_2_O_3_	1	1.0±0.2
7	Ni/SiO_2_−Al_2_O_3_	10	22.6±2.1

[a] Reaction conditions: oxaloacetate (30 mM), catalyst, 1 mL H_2_O (pH 7–8), 22 °C, 1 atm H_2_ (balloon), 18 h. [b] Yields represent mean values of three experiments with associated standard errors and were determined by quantitative ^1^H NMR spectroscopy with dimethyl sulfone as an internal standard.

A series of competition experiments was performed to compare selectivity among the reduction reactions. A mixture of equimolar amounts of oxaloacetate, fumarate, and pyruvate was subjected to the same reaction conditions (22 °C, hydrogen balloon, pH 7). NMR analysis revealed that the alkene of fumarate is reduced the fastest as compared to the ketone of oxaloacetate and pyruvate. Rh/Al_2_O_3_ (1 mol %) and Ni/SiO_2_−Al_2_O_3_ (10 mol %) show both roughly a 2 : 1 selectivity for the reduction of oxaloacetate over pyruvate at *t*=15 min (Figures S34 and S35) After both reactions were studied independently, we next wished to investigate the sequence of oxaloacetate to succinate in one‐ pot (Figure 2A). Therefore, the reaction parameters, like H_2_ pressure, temperature, pH, and catalyst loading, were varied to study their influence on the product distribution in the reduction of oxaloacetate compared to the standard conditions of pH 7, 22 °C with 1 atm H_2_ (balloon). These experiments are summarized in Figure 2, whereas the full details are given in the Supporting Information. For the Rh/Al_2_O_3_ catalyst (1 mol %), increasing the H_2_ pressure up to 10 bar did not show a large influence on the reaction compared to the standard reaction conditions. Additionally, the reaction still delivered malate in 84 % yield even when the temperature was decreased to 5 °C. Increasing the temperature to 40 or 60 °C led to 40 % and 16 % yield of malate, respectively, as the decarboxylation to pyruvate becomes dominating at higher temperatures. The yield of malate remained near 80 % even when highly acidic (pH 2) or alkaline (pH 11) conditions were employed, showing that the Rh catalyzed hydrogenation in water is highly robust to pH variation. However, in the experiment at pH 2, succinate was also observed in 5 % yield. To determine whether malate is an intermediate in the reaction sequence, it was individually resubjected to the reaction conditions (SI Table S8). Succinate was indeed obtained in 1 % yield at pH 2 or at neutral pH. The lower yield of succinate obtained when starting from malate than when starting from oxaloacetate suggests that a metal‐bound intermediate of oxaloacetate reduction, like a metal‐alkoxide, might also directly eliminate to form fumarate *in situ*. Finally, decreasing the catalyst loading of Rh/Al_2_O_3_ by an order of magnitude to 0.1 mol % (31 ppm Rh in the reaction mixture) still resulted in a 45 % yield of malate, indicating that very small quantities of Rh are sufficient for robust catalytic reduction of oxaloacetate (Figure 2B).

**Figure 2:**
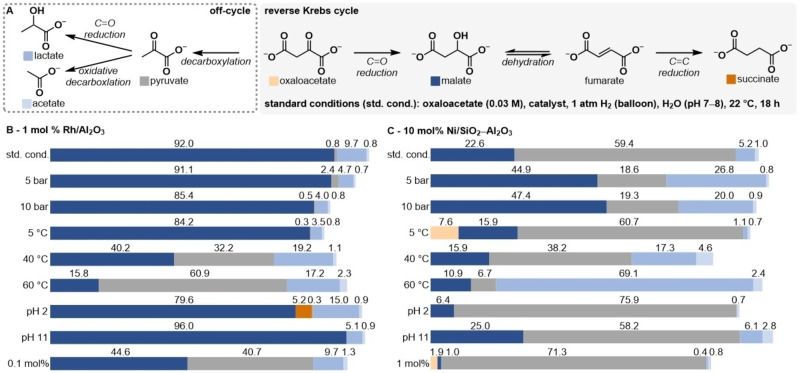
Variations of the standard reaction conditions (std. cond.) A) 30 mM oxaloacetate, 1 mL H_2_O (pH 7–8), 22 °C, 1 atm H_2_ (balloon), 18 h catalyzed by B) Rh/Al_2_O_3_ (1 mol %) or C) Ni/SiO_2_−Al_2_O_3_ (10 mol %). Absolute yields in B and C were determined by quantitative ^1^H NMR spectroscopy with dimethyl sulfone as an internal standard and represent mean values of at least two experiments. Standard errors are given in the Supporting Information.

For Ni/SiO_2_−Al_2_O_3_ (10 mol %), increasing the H_2_ pressure to 5 or 10 bar improved the yield of malate to 45 % and 47 %, respectively, compared to the standard conditions at 1 atm. Decreasing the reaction temperature from 22 °C to 5 °C resulted in a slight decrease in yield from 23 % to 16 %. However, unlike Rh, with Ni, the reaction functions best under alkaline conditions (pH 11) but poorly under acidic conditions (pH 2). Decreasing the catalyst loading by an order of magnitude from 10 mol % to 1 mol % resulted in a near total loss of reactivity; around 1 % of malate was obtained in this case (Figure 2C). Several general observations can therefore be made regarding the studied hydrogenations. First, for both catalysts, the yield of malate is the highest at 22 °C or below, as decarboxylation competes at higher temperatures. Second, Rh is a much more effective catalyst than Ni at low catalyst loadings and is also more robust with respect to variations in pH and H_2_ pressure, exhibiting <20 % variation in yield across the entire pH and pressure range tested. Ni shows the highest catalytic activity at higher H_2_ pressure and pH values >5 and generally requires much higher catalyst loadings to achieve similar yields than Rh. These observations are in line with the general trend that precious metal catalysts tend to be more active than first‐row transition metals since they are less prone to forming catalytically inactive higher order clusters and nanoparticles.[^45^, ^46^] Third, acidic pH values are best to facilitate the dehydration of malate to fumarate at 22 °C.

To obtain further insight into the hydrogenations under Rh/Al_2_O_3_ and Ni/SiO_2_−Al_2_O_3_ catalysis, we followed the reaction progress for both catalysts over two days at 5 °C and pH 2 under hydrogen atmosphere (balloon) by taking aliquots of the reaction mixture at different time points. Kinetic plots are shown in Figure S68. It was found that succinate could be obtained in 5 % yield with 1 mol % Rh/Al_2_O_3_ after 3.5 h, and in 0.1 % yield with 20 mol % Ni/SiO_2_‐Al_2_O_3_ after 9 h.

Based on these promising results, we elected to assay geological samples as catalysts. Fe meteorites are representative of the objects that impacted the Earth early in its history and are rich in both nickel and platinum group metals, with nickel being found in quantities of up to 33 % by mass.^[47]^ In addition, the catalytic properties of meteorites have been reported,[^48^, ^49^] including in a prebiotic context.[^50^, ^51^, ^52^] Three different meteorites (Campo del Cielo, Gibeon, Sikhote Alin; see Supporting Information for more details) were purchased and ground into a powder. The compositions of these meteorites have previously been characterized.^[53]^ For example, an analysis of the Campo del Cielo meteorite found it to contain 6.7 % Ni by weight and 2.39 ppm of Rh.^[47]^ Therefore, we assayed the meteoritic powders as catalysts for the reduction of oxaloacetate at pH 7 and 22 °C under 5 bar H_2_ (Table 3). Under these conditions, malate was observed with all three meteorites (10 mg) in yields between 14 and 19 % (entries 1—3). Corresponding control experiments in the absence of H_2_ did not produce any detectable malate, demonstrating that the metals present in the meteorite are not themselves acting as reducing agents. When similar experiments were carried out at pH 2 and 1 atm of H_2_, succinate could be observed in trace quantities, indicating that all reactions of the sequence can occur in one‐pot in the presence of meteorites (Table S15).

**Table 3:** Reduction of oxaloacetate promoted by iron meteorites.^[a]^

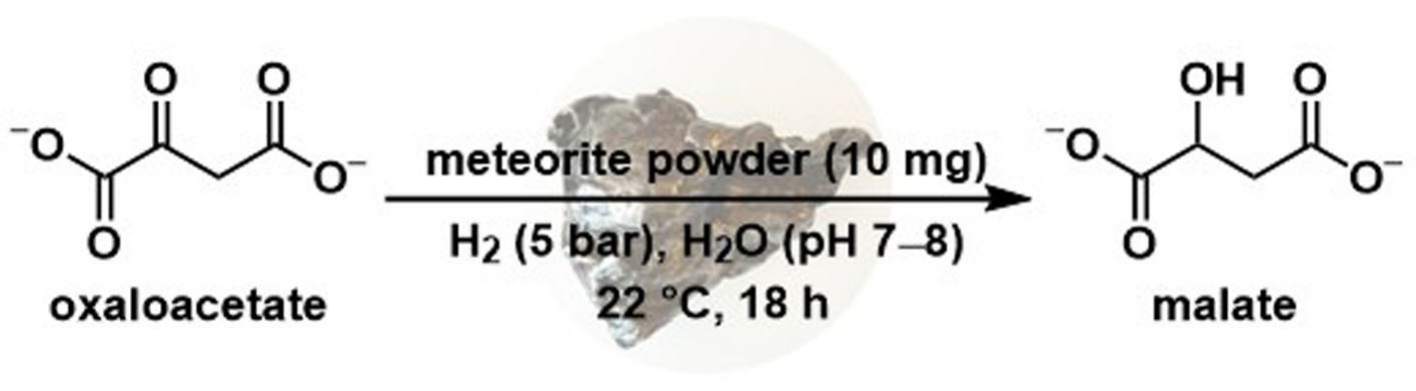					
Entry	Meteorite	Amount [mg]			Yield [%]^[b]^
1	Campo del Cielo	10			14.8±2.7
2	Gibeon	10			18.9±0.3
3	Sikhote Alin	10			14.1±2.1

[a] Reaction conditions: oxaloacetate (30 mM), catalyst, 1 mL H_2_O (pH 7–8), 22 °C, H_2_ (5 bar), 18 h. [b] Yields represent mean values of two experiments with associated standard errors and were determined by quantitative ^1^H NMR spectroscopy with dimethyl sulfone as an internal standard.

In summary, we have identified metallic and meteoritic catalysts capable of using H_2_ to enable both reduction reactions in the first half of the reverse Krebs cycle under biologically compatible conditions in water. The reactions occur at ambient temperature, or even at 5 °C, at near‐neutral pH and atmospheric pressure. Furthermore, the reduction and dehydration reactions of the first half of the reverse Krebs cycle were found to occur under mutually compatible conditions, allowing oxaloacetate to be directly converted to succinate with H_2_ as the reducing agent under mild conditions potentially relevant to the emergence of the metabolic network. The platinum‐group metal Rh was found to act as a hydrogenation catalyst for these reactions in water at loadings at least as small as 0.1 mol %, whereas catalyst loadings of 10 mol % are required if nickel on silica‐alumina is used as catalyst under the same conditions. This work therefore constitutes a rare example of catalytic turnover (i.e., TON >1) in prebiotic catalysis.^[54]^ Furthermore, the observation that meteorite samples also catalyze these reactions strengthens the geological plausibility of a H_2_‐driven prebiotic analog of a partial reverse Krebs cycle. However, it should be noted that the heterogeneous composition of a meteorite does not allow us to attribute this reactivity to a particular metal or mixture of metals. Nickel is found in the active sites of some enzymes like the [FeNi]‐hydrogenases,^[55]^ whereas platinum group metals like Rh are not. However, it may be premature to dismiss Rh in a prebiotic context since it is found in meteorites. Platinum metals are found to be locally concentrated in nature in amounts many orders of magnitude higher than their average abundance on earth,[^56^, ^57^, ^58^] and simple mechanisms for the concentration of Rh have been described.^[59]^ Perhaps more importantly, self‐organized chemistry can happen on local, microscopic scales. Since the only stoichiometric inputs into protometabolic networks are thought to be ubiquitous gases, rare metal catalysts could still be useful to initiate reaction networks on local, microscopic scales. Complex organics produced by a localized proto‐metabolic reaction network could then ligate metals that are more widely abundant (e.g., Fe, Ni, etc.) to produce catalysts that then replace rare metal catalysts altogether, allowing the reaction network to migrate to new environments. In such an “initiator” role, a widespread abundance of rare metal catalysts on the early Earth would not be required—its concentrated presence in a single microscopic location may be sufficient. Nevertheless, it cannot be excluded that inorganic catalysts bearing a stronger semblance to modern enzymes will be discovered in the future. The conditions presented in this study are potentially relevant to environments with high natural concentrations of H_2_ over long time scales. The interface between alkaline hydrothermal vents and seawater displays gradients of pH (5–11) and temperature (2–60 °C) compatible with the conditions described here. Underground locations concentrating H_2_ generated from water radiolysis may also be consistent. Moving forward, we aim to constrain these environments by accounting for the conditions required to drive other critical protometabolic reactions and by incorporating them into larger integrated reaction networks.

## Conflict of interest

The authors declare no conflict of interest.

## Supporting information

As a service to our authors and readers, this journal provides supporting information supplied by the authors. Such materials are peer reviewed and may be re‐organized for online delivery, but are not copy‐edited or typeset. Technical support issues arising from supporting information (other than missing files) should be addressed to the authors.

Supporting Information

## Data Availability

The data that support the findings of this study are available in the supplementary material of this article.
