# Supplementary material for: Hydrogen Drives Part of the Reverse Krebs Cycle under Metal or Meteorite Catalysis
Source: Angew Chem Int Ed Engl. 2022 Nov 22;61(51):e202212932. doi: 10.1002/anie.202212932 (PMC10100321; doi:10.1002/anie.202212932)
Supplement: Supplementary file 1 — Supporting Information [file ANIE-61-0-s001.pdf]

Angewandte Chemie
Eine Zeitschrift der Gesellschaft Deutscher Chemiker

Supporting Information

Hydrogen Drives Part of the Reverse Krebs Cycle under Metal or Meteorite Catalysis

*S. A. Rauscher, J. Moran**

Table of Contents

1 General 2

1.1 Materials 2

1.2 Reaction set-up 2

1.3 NMR spectroscopy 2

1.4 Analytical methods 3

1.4.1 NMR standards 3

1.4.2 NMR quantification 8

2 Synthetic procedures and quantification 9

2.1 Catalyst screen for the reduction of fumarate to succinate 9

2.2 Catalyst screen for the reduction of oxaloacetic acid to malate 17

2.3 Competition experiment 26

2.4 Variation of reaction parameters for Rh/Al_2O_3 and Ni/SiO_2-Al_2O_3 27

2.4.1 Standard reaction conditions 27

2.4.2 Variation of H_2 pressure 30

2.4.3 Variation of temperature 34

2.4.4 Variation of pH 41

2.4.5 Variation of catalyst loading 45

2.5 Malate under standard reaction conditions 47

2.6 Reaction progress over time at 5 °C 52

2.7 Control reactions 60

2.7.1 Argon atmosphere 60

2.7.2 Support without metal 62

2.8 Reaction with meteorites 64

2.8.1 Classification and composition of iron meteorites 64

2.8.2 Meteorite screen for the reduction of oxaloacetate at 5 bar hydrogen 66

2.8.3 Meteorite screen for the reduction of oxaloacetate at 1 atm hydrogen 70

2.8.4 Control reaction 72

3 References 77

1 General

1.1 Materials

Oxaloacetic acid (≥97%), DL-malic acid (ReagentPlus®, ≥99%), sodium fumarate dibasic (ReagentPlus®, ≥99%), succinic acid (BioXtra, ≥99.0%), sodium pyruvate (ReagentPlus®, ≥99%), lactic acid (ReagentPlus®, ≥99.0%), and malonic acid (ReagentPlus®, 99%) were purchased from Sigma-Aldrich. Ammonium acetate (≥99 %) was purchased from Fluka. Chemicals were used without further purification. The purity of chemicals was verified before use by quantitative NMR spectroscopy (qNMR).

Rh/Al_2O_3 (5% Rh), Pd/C (10% Pd), $Pd(OH)_2/C$ (20% Pd), $Pd/BaSO_4$ (5% Pd), Ru/C (5% Ru), Ni powder (<150 µm ,99.99%), $NiCl_2 \cdot 6\ H_2O$, and $CoCl_2$ were purchased from Sigma-Aldrich. Pt/C (5% Pt), Pt/Al_2O_3 (1% Pt), and $FeCl_2$ were purchased from Alfa Aesar. Ni/SiO_2-Al_2O_3 (67.9% Ni) and $CoAl_2O_4$ (39.69% Co) were purchased from Thermo Fisher Scientific. Ru/Al_2O_3 (5% Ru) and Co-Mo/Al_2O_3 (cobalt oxide 3.4−4.5%, molybdenum oxide 11.5−14.5% on alumina) were purchased form ABCR. $RhCl_3$, $PtCl_2$, $PdCl_2$, and Fe powder (99.9%) were purchased from Strem.

The meteorites Campo del Cielo, Gibeon, and Sikhote Alin were purchased from Decker Meteorite-Shop. To use the meteorites in the reaction, material was removed from the meteorite with a 150 mm file, and further mortared in an agate mortar. More details to the chemical composition of the meteorites are given in section 2.8.

Water used for preparation of buffers or solutions was obtained from a Sartorius Arium purification system (18.2 MΩcm).

Phosphate buffer (1 M, pH 3) used for the preparation of NMR samples was prepared from mixing phosphoric acid (1 M, 85 wt. % in H_2O) and $KHPO_4$ (1 M, ACS reagent, ≥98%, Sigma-Aldrich) to obtain pH 3 as monitored by a pH meter calibrated before use.

1.2 Reaction set-up

Reactions at 1 atm H_2 (balloon) were conducted in 4 mL glass vials with PTFE/silicone-lined caps equipped with a PTFE stir bar. Reactions at 5 or 10 bar H_2 were conducted in a stainless-steel pressure reactor from Equilabo in 1.5 mL glass vials with pierced PTFE-lined caps equipped with a PTFE stir bar. Reaction at 5 °C were controlled by an immersion cooler from Huber (model TC100E). New PTFE stir bars were used for the reactions with meteorites to exclude contamination.

1.3 NMR spectroscopy

NMR spectra were recorded on a Bruker Avance Neo-500 (500 MHz) or a Bruker UltraShield Plus Avance III spectrometer (400 MHz) equipped with Prodigy BBO cryoprobes at a sample temperature maintained at 23 °C. NMR shifts are reported in ppm. Water suppression in ^1H NMR spectra was achieved using the Bruker noesygppr1d pulse program acquiring 8 scans for each sample. For quantitative NMR spectra (qNMR) the relaxation delay d1 was set to 65 s for reaction containing fumarate or 36 s for reactions without fumarate after having determined the T1 relaxation times of all relevant resonances with the inversion recovery method.
Dimethyl sulfone (DMS, Sigma-Aldrich, TraceCERT) in D_2O was used as internal standard (CH_3 group set to 3.0000 ppm).

1.4 Analytical methods

1.4.1 NMR standards

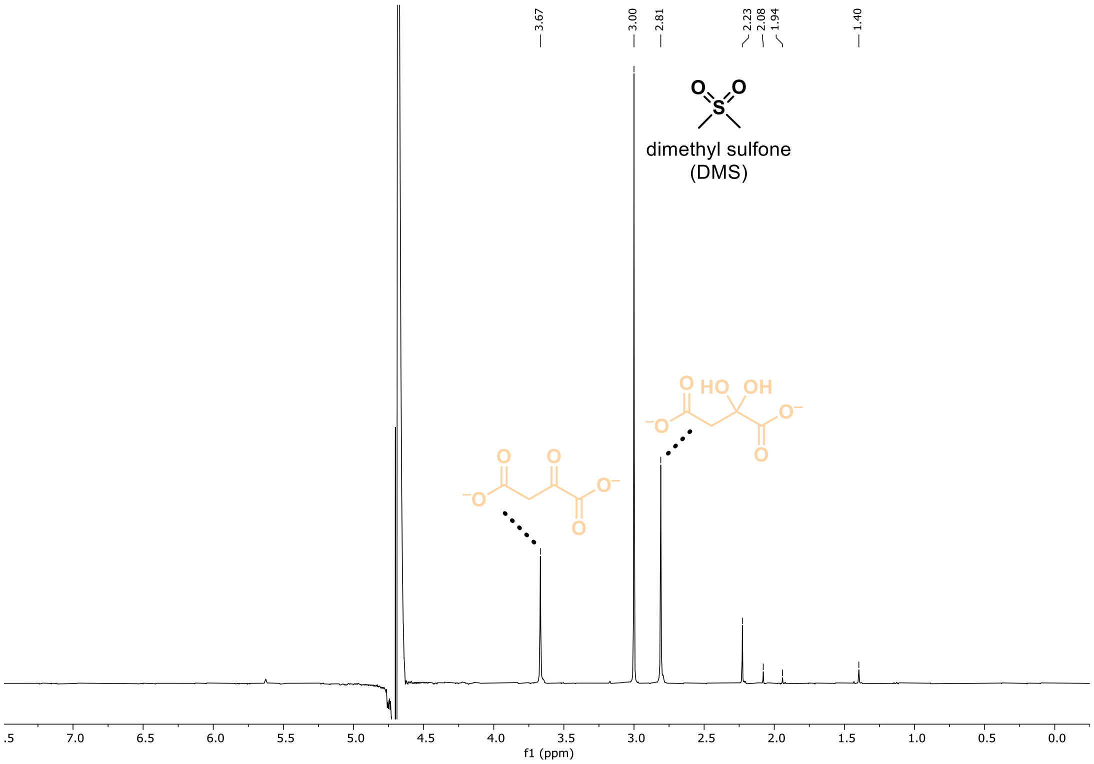

Figure S1. ^{1}H NMR of oxaloacetate with DMS as internal standard at pH 3. The peaks at 2.23 and 1.40 ppm correspond to pyruvate that spontaneously formed by decarboxylation of oxaloacetate. The singlet at 1.94 ppm corresponds to acetate and the singlet at 2.08 ppm corresponds to an impurity (most likely acetone).

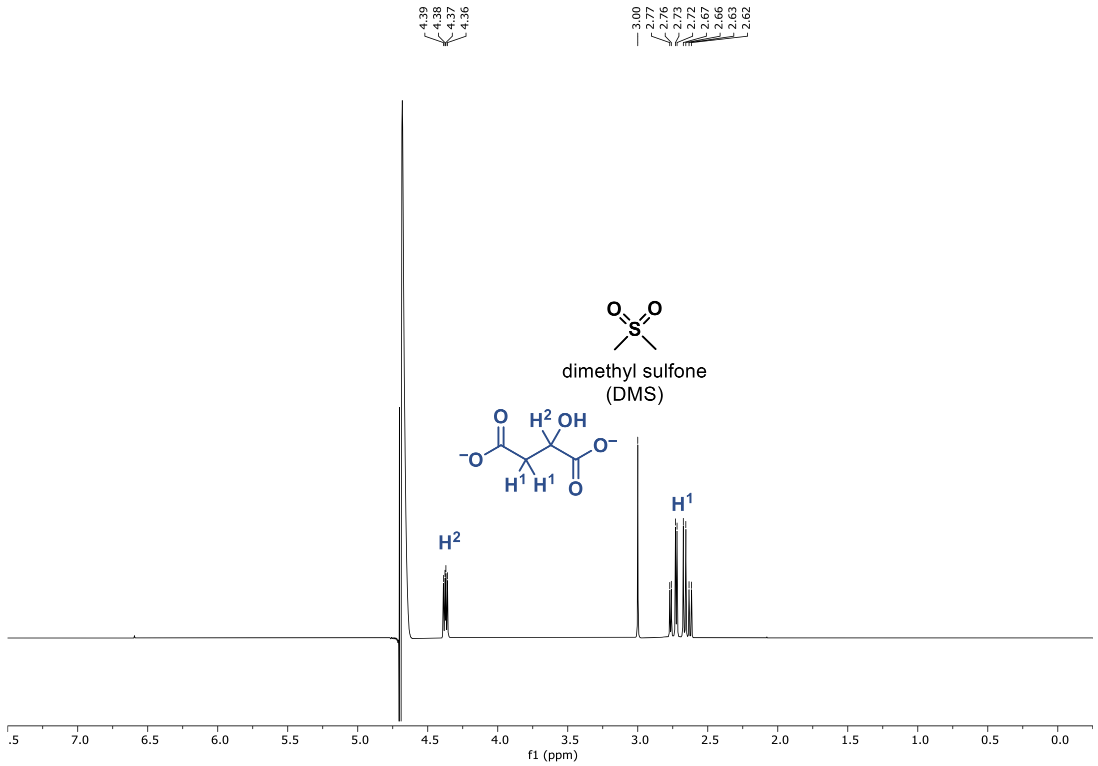

Figure S2. ^{1}H NMR of malate with DMS as internal standard at pH 3.

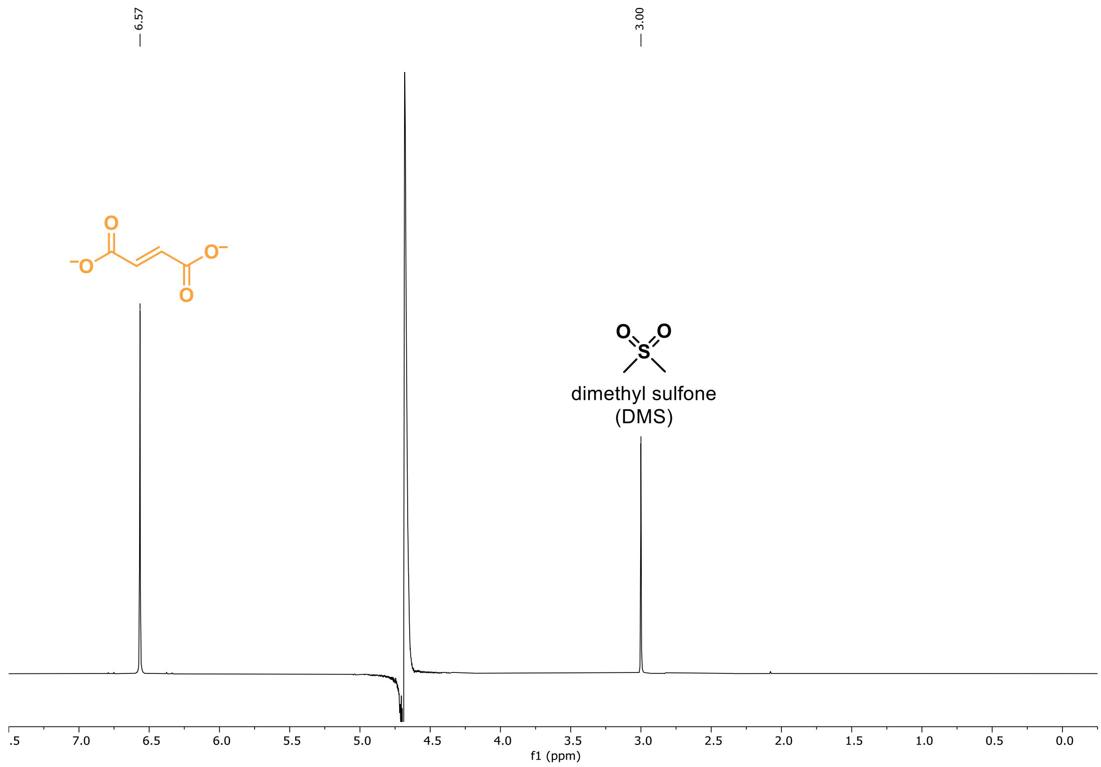

Figure S3. ^{1}H NMR of fumarate with DMS as internal standard at pH 3.

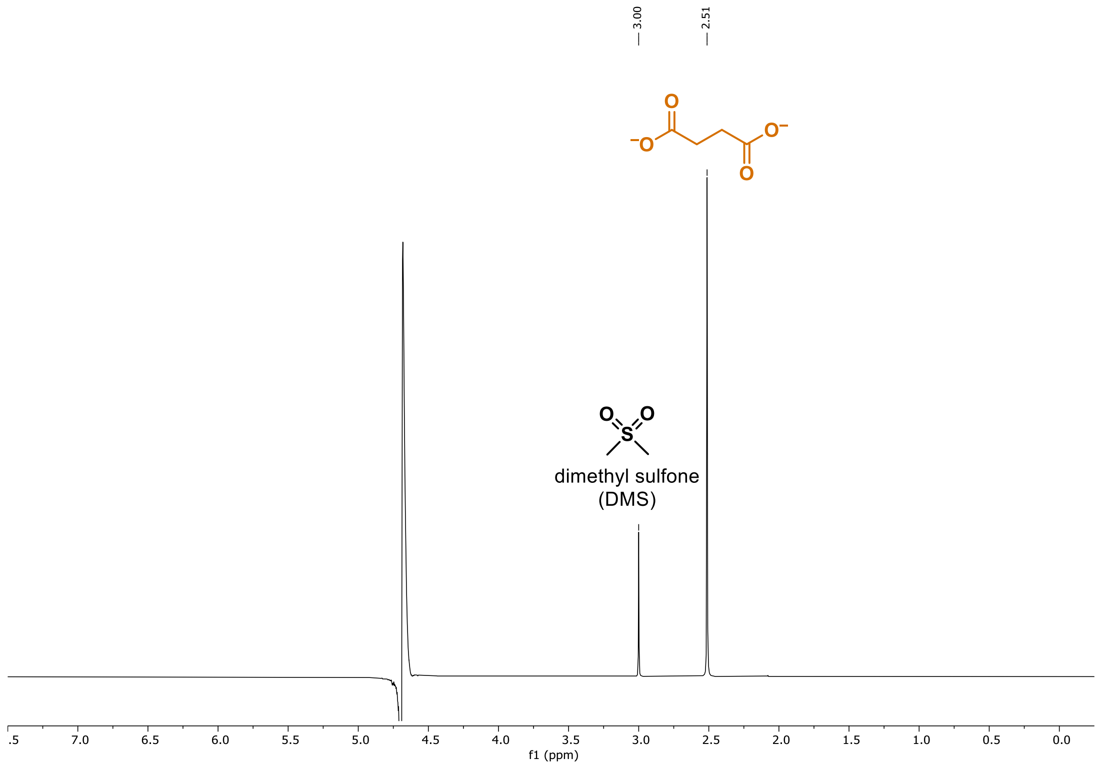

Figure S4. ^{1}H NMR of succinate with DMS as internal standard at pH 3.

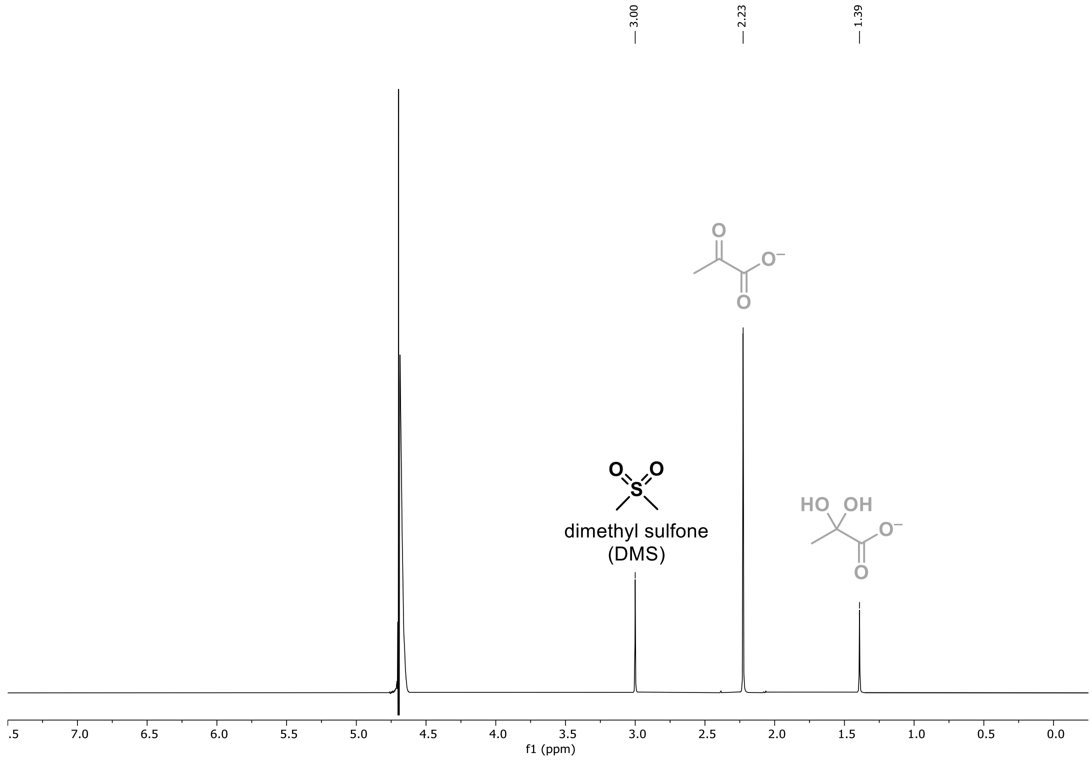

Figure S5. ^{1}H NMR of pyruvate with DMS as internal standard at pH 3.

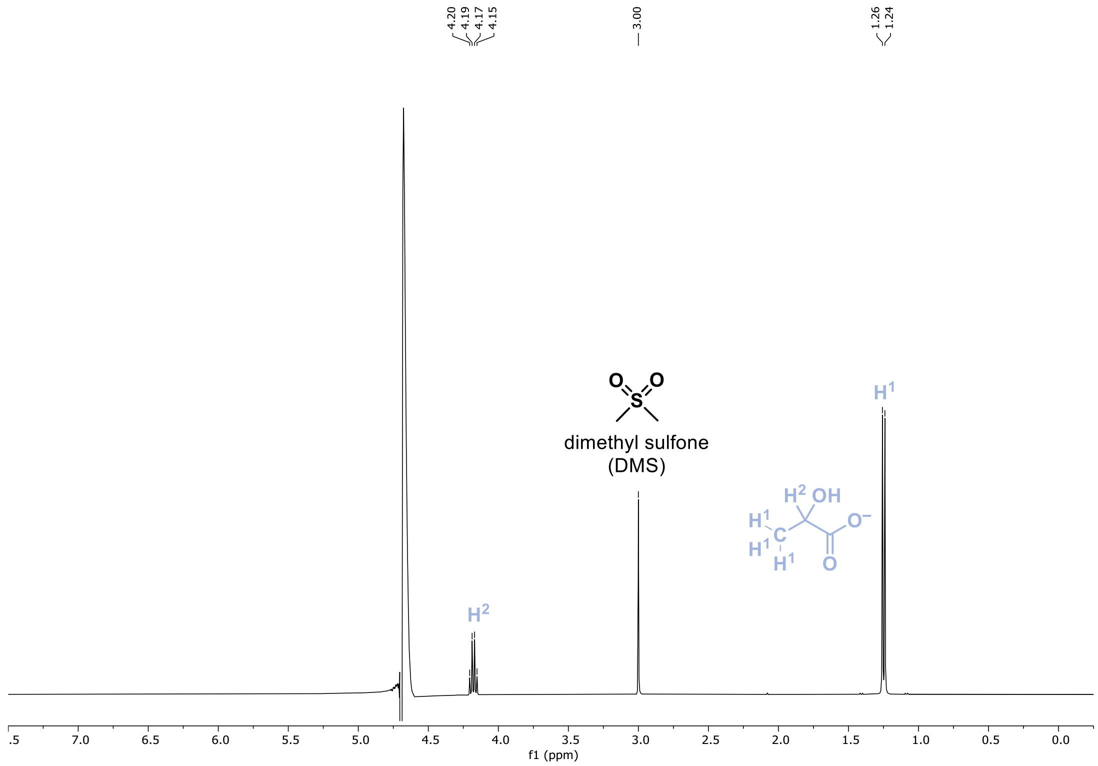

Figure S6. ^{1}H NMR of lactate with DMS as internal standard at pH 3.

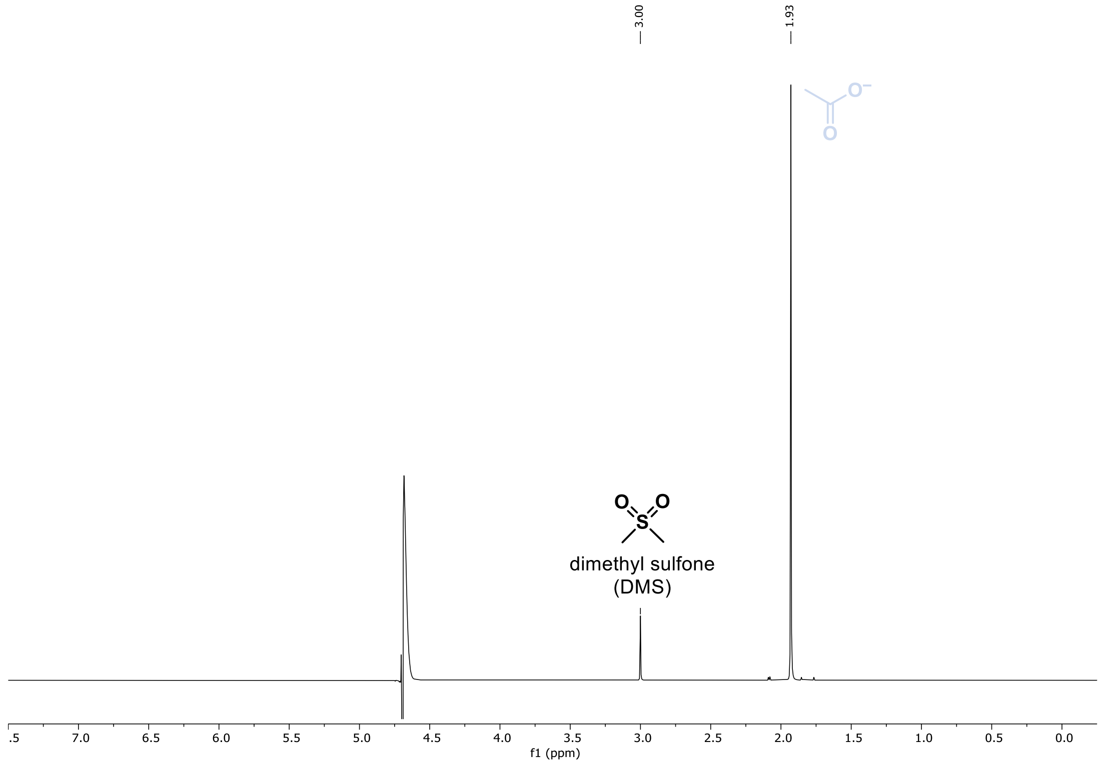

Figure S7. ^{1}H NMR of acetate with DMS as internal standard at pH 3.

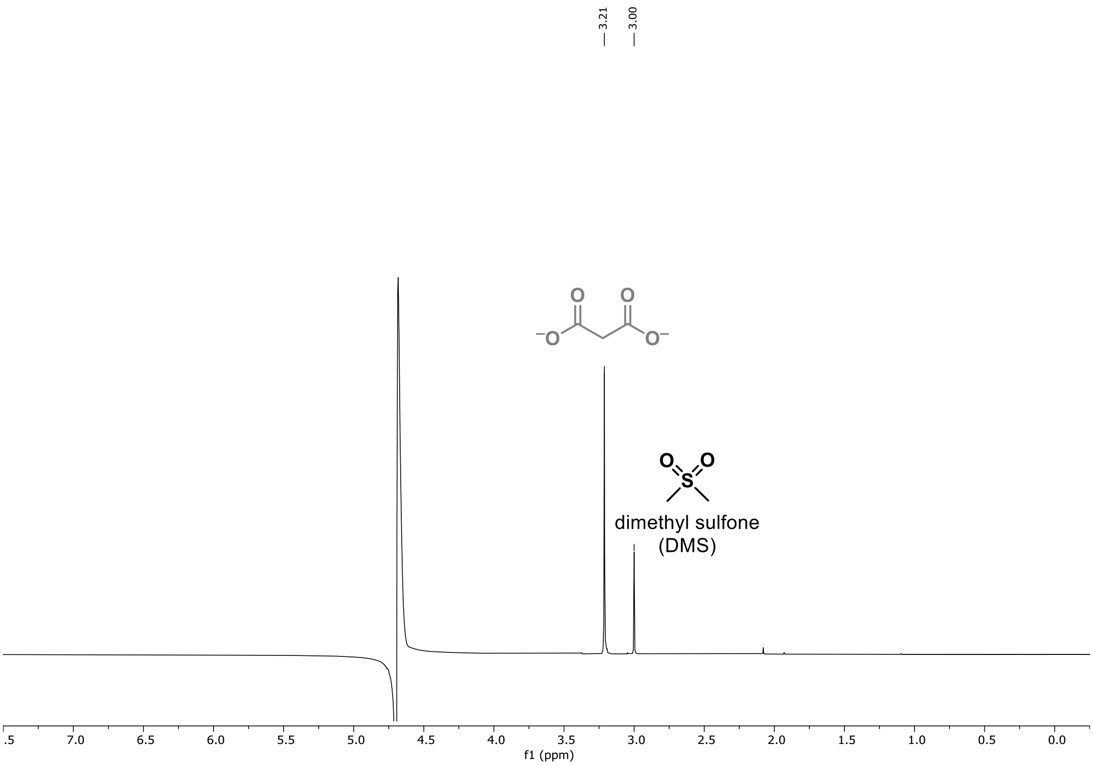

Figure S8. ^{1}H NMR of malonate with DMS as internal standard at pH 3.

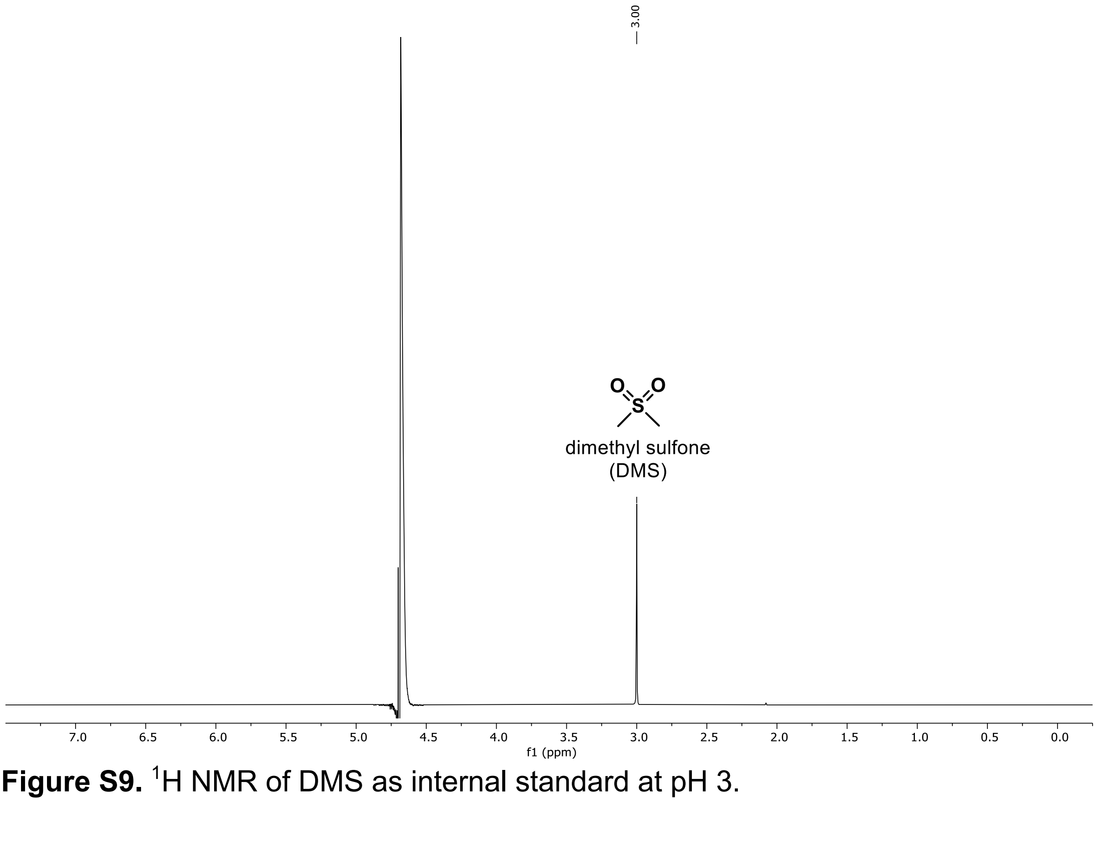

Figure S9. ^{1}H NMR of DMS as internal standard at pH 3.

1.4.2 NMR quantification

Reactions were analyzed by quantitative ^{1}H NMR (qNMR) using 200 µL of centrifuged reaction mixture, 350 µL of phosphate buffer (1 M, pH 3), and 50 µL of a DMS stock solution in D_2O (total volume of NMR sample: 600 µL). Reaction mixtures in the section 2.8 were subjected to Chelex® resin to remove metal ions prior to NMR analysis. Therefore, 400 µL of centrifuged reaction was added to 200 mg Chelex® resin and placed in a shaker for 20 min, and 200 µL of this solution were subsequently used for quantitative ^{1}H NMR analysis. Concentrations of DMS stock solutions used for quantification are given in the tables for each reaction in section 2.

Since some compounds exhibit a shift in the NMR signals with a change in pH, it was necessary to control the pH of the NMR samples. To achieve good peak separation and facilitate peak integration, pH 3 was chosen. Unless otherwise noted, peaks marked with a black asterisk in the Figure S10 were used for quantification.

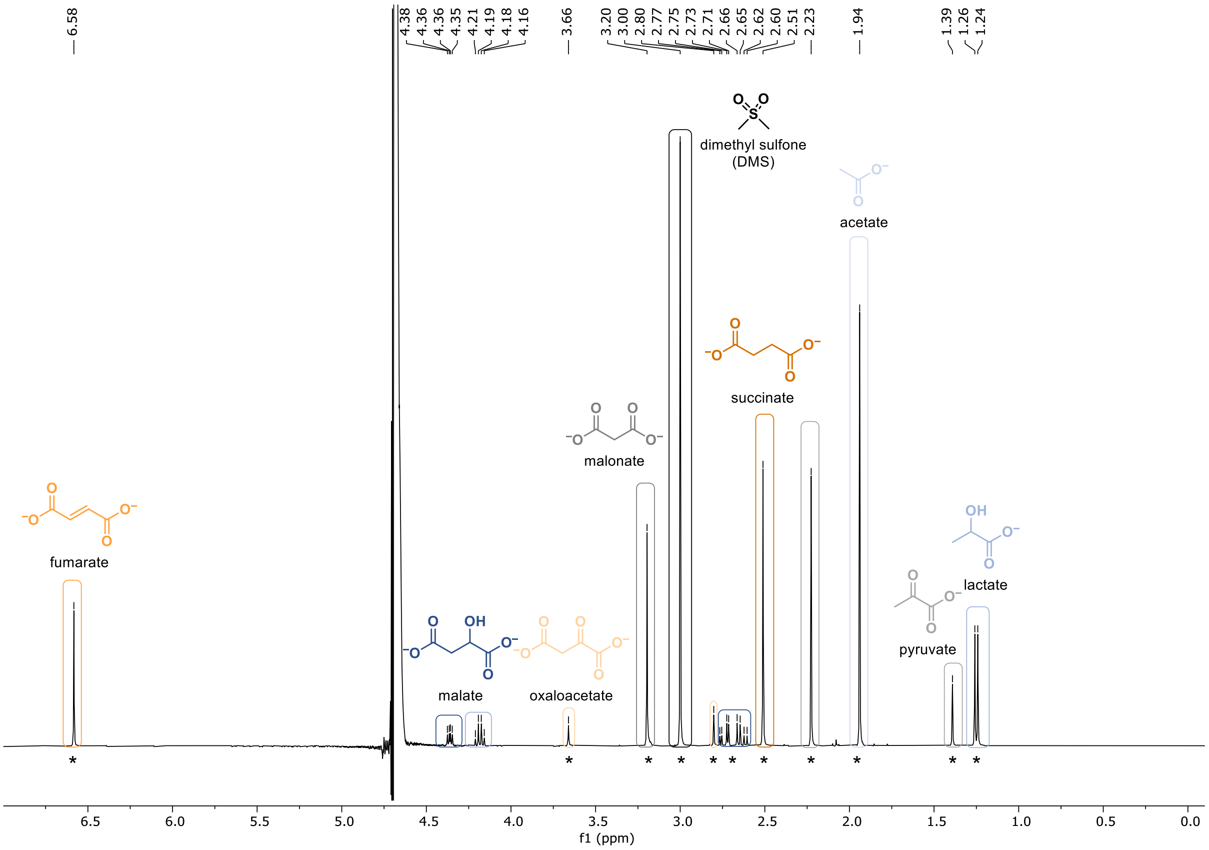

Figure S10. ^{1}H NMR of authentic: oxaloacetate, malate, fumarate, succinate, pyruvate, lactate, acetate, and malonate with dimethyl sulfone (DMS) as internal standard at 3.0000 ppm.

2 Synthetic procedures and quantification

2.1 Catalyst screen for the reduction of fumarate to succinate

A disposable glass vial with PTFE/silicone-lined septum (see section 1.2 for more details) was charged with the metal catalyst (0.01 equiv., 1 mol%). $RhCl_3$, $PtCl_2$, and $PdCl_2$ were added from a stock solution (100 µL, 3 mM) and heterogeneous catalyst were added directly to the reaction vial. Then, sodium fumarate dibasic (0.03 mmol, 4.80 mg, 1 equiv.) was added from an aqueous stock solution (0.06 M adjusted to pH 7−8 with NaOH) and water was added to a total volume of 1 mL solvent. After purging the vial for 3 min with H_2, the vial equipped with an H_2 balloon was stirred at 22 °C for 18 h. Experiments were done in triplicates. qNMRs were acquired according to section 1.4.2. Mean values of yields and standard errors were calculated for each set of reactions.

Table S1. Catalyst screen - Reduction reaction of sodium fumarate dibasic to succinate.

			integrals relative to DMS (6H) set to 1.0000 at 3.0000 ppm		yield [%]	
**#**	**catalyst loading (equiv., mol%, mg)**	**conc. DMS of stock solution [M]**	**fumarate (2H)**	**succinate (4H)**	**fumarate**	**succinate**
**1**	$RhCl_3$ (0.01 equiv., 1 mol%, 0.08 mg)	0.02221	0.0060	3.4655	0.3	96.2
**2**	$RhCl_3$ (0.01 equiv., 1 mol%, 0.08 mg)	0.02221	0	3.4992	0	97.1
**3**	$RhCl_3$ (0.01 equiv., 1 mol%, 0.08 mg)	0.02221	0	3.4785	0	96.6
					0.1 ± 0.1	96.6 ± 0.3
**4**	$PtCl_2$ (0.01 equiv., 1 mol%, 0.08 mg)	0.02221	0.4095	2.8742	22.7	79.8
**5**	$PtCl_2$ (0.01 equiv., 1 mol%, 0.08 mg)	0.02221	0.4042	2.7188	22.4	75.5
**6**	$PtCl_2$ (0.01 equiv., 1 mol%, 0.08 mg)	0.02221	0.4252	2.6593	23.6	73.8
					22.9 ± 0.4	76.4 ± 1.8
**7**	$PdCl_2$ (0.01 equiv., 1 mol%, 0.05 mg)	0.02221	0	3.3736	0	93.7
**8**	$PdCl_2$ (0.01 equiv., 1 mol%, 0.05 mg)	0.02221	0	3.5076	0	97.4
**9**	$PdCl_2$ (0.01 equiv., 1 mol%, 0.05 mg)	0.02221	0	3.4620	0	96.1
					0 ± 0	95.7 ± 1.1
**10**	Rh/Al_2O_3 (0.01 equiv., 1 mol%, 0.62 mg)	0.02027	0	3.9017	0	98.9
**11**	Rh/Al_2O_3 (0.01 equiv., 1 mol%, 0.62 mg)	0.02027	0	3.9042	0	98.9
**12**	Rh/Al_2O_3 (0.01 equiv., 1 mol%, 0.62 mg)	0.02027	0	3.9228	0	99.4
					0 ± 0	99.1 ± 0.2
**13**	Pd/C (0.01 equiv., 1 mol%, 0.32 mg)	0.02027	0	3.9504	0	100.1
**14**	Pd/C (0.01 equiv., 1 mol%, 0.32 mg)	0.02027	0	3.9703	0	100.6
**15**	Pd/C (0.01 equiv., 1 mol%, 0.32 mg)	0.02027	0	3.9045	0	98.9
					0 ± 0	99.9 ± 0.5
**16**	$Pd(OH)_2/C$ (0.01 equiv., 1 mol%, 0.16 mg)	0.02027	0	3.9755	0	100.7
**17**	$Pd(OH)_2/C$ (0.01 equiv., 1 mol%, 0.16 mg)	0.02027	0	3.8741	0	98.2
**18**	$Pd(OH)_2/C$ (0.01 equiv., 1 mol%, 0.16 mg)	0.02027	0.0069	3.8685	0.3	98.0
					0.1 ± 0.1	99.0 ± 0.9

			Integrals relative to DMS (6H) set to 1.0000 at 3.0000 ppm		yield [%]	
#	catalyst loading (equiv., mol%, mg)	conc. DMS [M]	fumarate (2H)	succinate (4H)	fumarate	succinate
19	Pd/$BaSO_4$ (0.01 equiv., 1 mol%, 0.64 mg)	0.02027	0	3.9093	0	99.1
20	Pd/$BaSO_4$ (0.01 equiv., 1 mol%, 0.64 mg)	0.02027	0	3.9765	0	100.8
21	Pd/$BaSO_4$ (0.01 equiv., 1 mol%, 0.64 mg)	0.02027	0	3.8993	0	98.8
					0 ± 0	99.5 ± 0.6
22	Pt/C (0.01 equiv., 1 mol%, 1.72 mg)	0.02027	0	3.9230	0	99.4
23	Pt/C (0.01 equiv., 1 mol%, 1.72 mg)	0.02027	0.0062	3.8798	0.3	98.3
24	Pt/C (0.01 equiv., 1 mol%, 1.72 mg)	0.02027	0	3.9135	0	99.2
					0.1 ± 0.1	99.0 ± 0.3
25	Pt/Al_2O_3 (0.01 equiv., 1 mol%, 5.85 mg)	0.02027	0.1875	3.5813	9.5	90.7
26	Pt/Al_2O_3 (0.01 equiv., 1 mol%, 5.85 mg)	0.02027	0.1664	3.6308	8.4	92.0
27	Pt/Al_2O_3 (0.01 equiv., 1 mol%, 5.85 mg)	0.02027	0.1967	3.5289	10.0	89.4
					9.3 ± 0.5	90.7 ± 0.7
28	Ni/SiO_2-Al_2O_3 (0.1 equiv., 10 mol%, 0.26 mg)	0.02027	0	3.9329	0	99.6
29	Ni/SiO_2-Al_2O_3 (0.1 equiv., 10 mol%, 0.26 mg)	0.02027	0	3.9510	0	100.1
30	Ni/SiO_2-Al_2O_3 (0.1 equiv., 10 mol%, 0.26 mg)	0.02027	0	3.7880	0	96.0
					0 ± 0	98.6 ± 1.3
31[a]	Ni/SiO_2-Al_2O_3 (0.01 equiv., 1 mol%, 0.13 mg)	0.02027	1.6518	1.0843	83.7	27.5
32[a]	Ni/SiO_2-Al_2O_3 (0.01 equiv., 1 mol%, 0.13 mg)	0.02027	1.4021	1.5467	71.1	39.2
33[a]	Ni/SiO_2-Al_2O_3 (0.01 equiv., 1 mol%, 0.13 mg)	0.02027	1.5459	1.2379	78.3	31.4
					77.7 ± 3.7	32.7 ± 3.4
34	none	0.01841	2.4155	0	98.7	0
35	none	0.01841	2.1685	0	99.8	0
36	none	0.01841	2.1677	0	99.8	0
					99.4 ± 0.3	0 ± 0

[a] Reaction conditions: sodium fumarate dibasic (0.15 mmol, 24.0 mg, 1 equiv.) in 5 mL H_2O (pH adjusted to pH 7−8 with NaOH), Ni/SiO_2-Al_2O_3 (0.01 equiv., 1 mol%, 0.13 mg), H_2 (1 atm), pH 7−8, 22 °C, 18 h.

Metals without reduction reactivity under the above-mentioned reaction conditions: Rh/C, Ru/Al_2O_3, $NiCl_2$, NiO, $CoCl_2$, Co-Mo/Al_2O_3 (cobalt oxide 3.4−4.5%, molybdenum oxide 11.5−14.5% on alumina), $CoAl_2O_4$ (39.69%), $FeCl_2$.

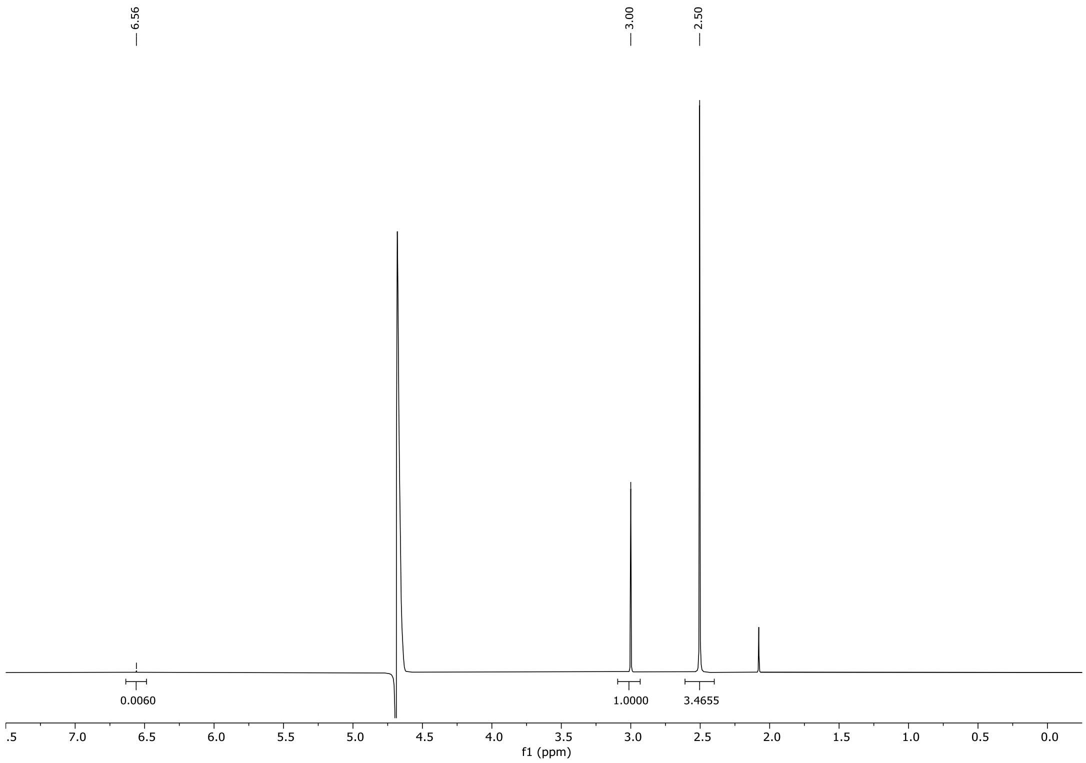

Figure S11. Representative ^{1}H qNMR using $RhCl_3$ as catalyst (Table S1 entry 1−3).

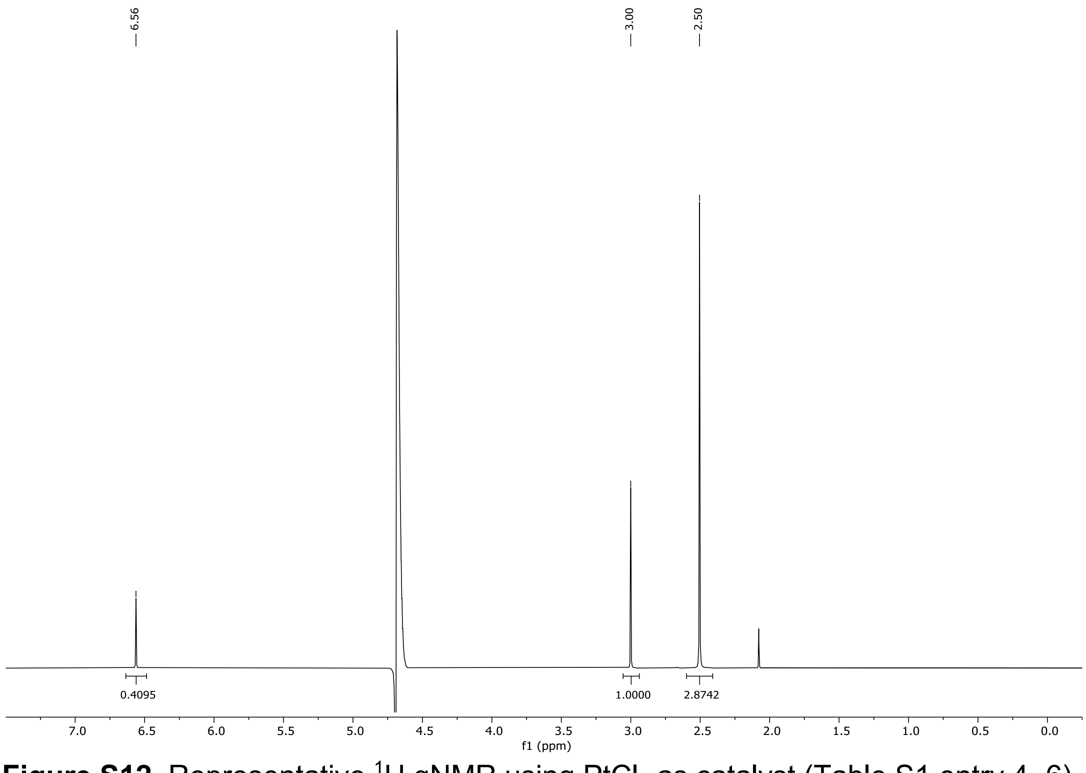

Figure S12. Representative ^{1}H qNMR using $PtCl_2$ as catalyst (Table S1 entry 4−6).

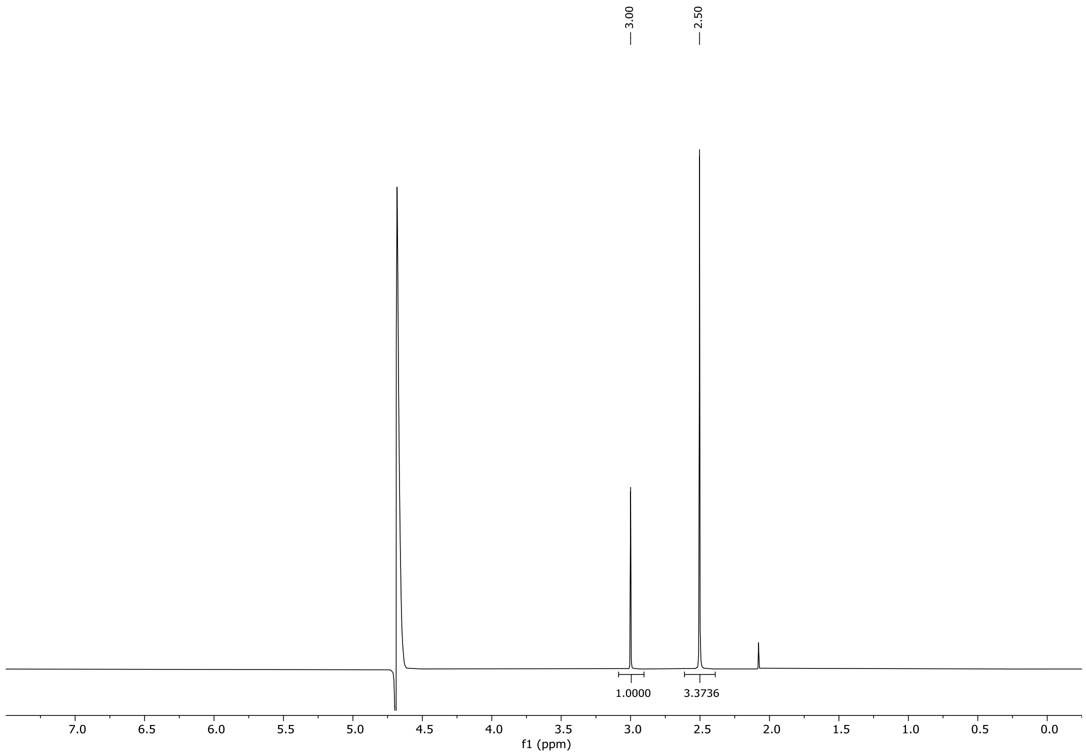

Figure S13. Representative ^{1}H qNMR using $PdCl_2$ as catalyst (Table S1 entry 7−9).

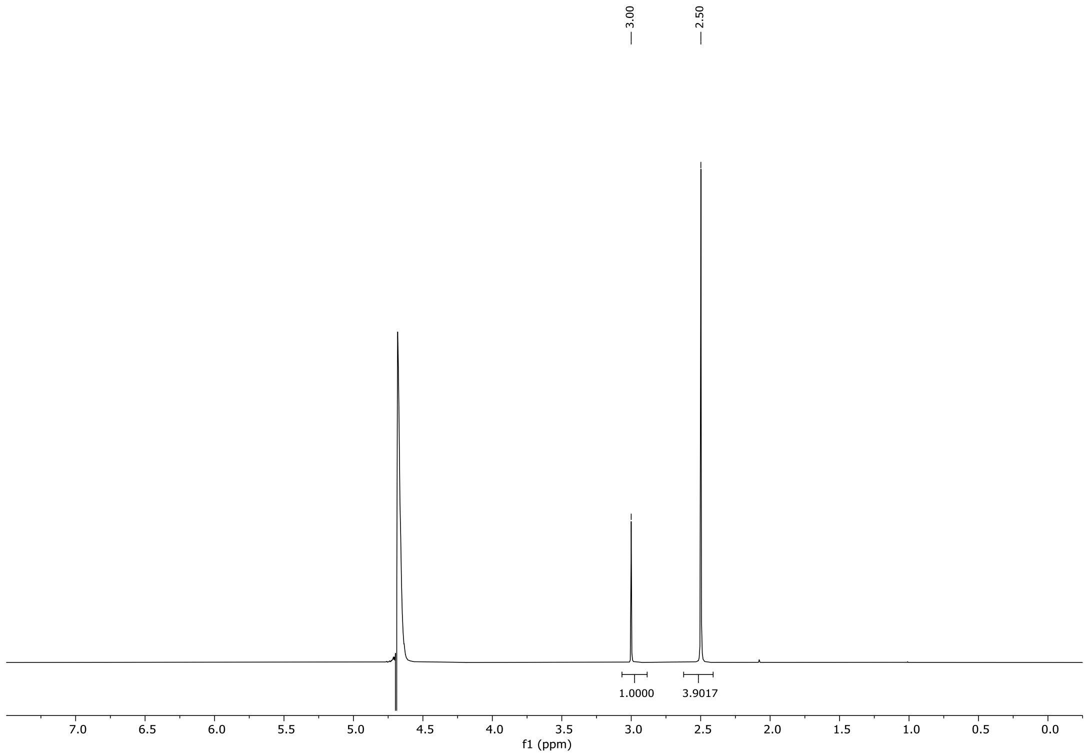

Figure S14. Representative ^{1}H qNMR using Rh/Al_2O_3 as catalyst (Table S1 entry 10−12).

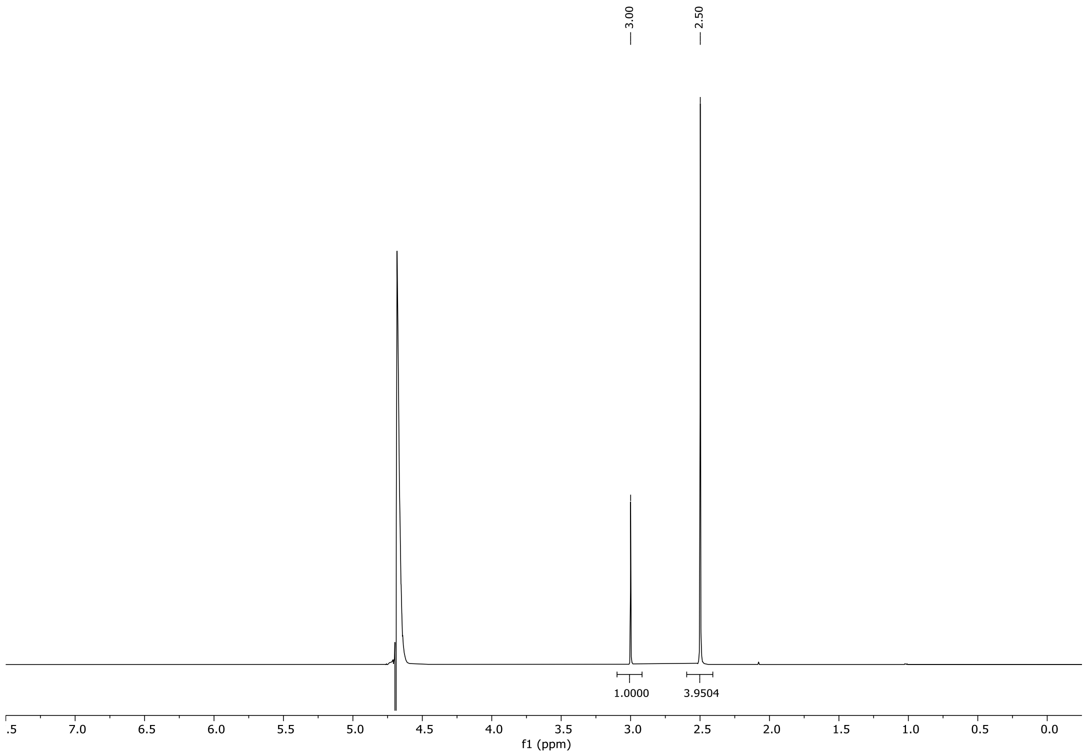

Figure S15. Representative ^{1}H qNMR using Pd/C as catalyst (Table S1 entry 13−15).

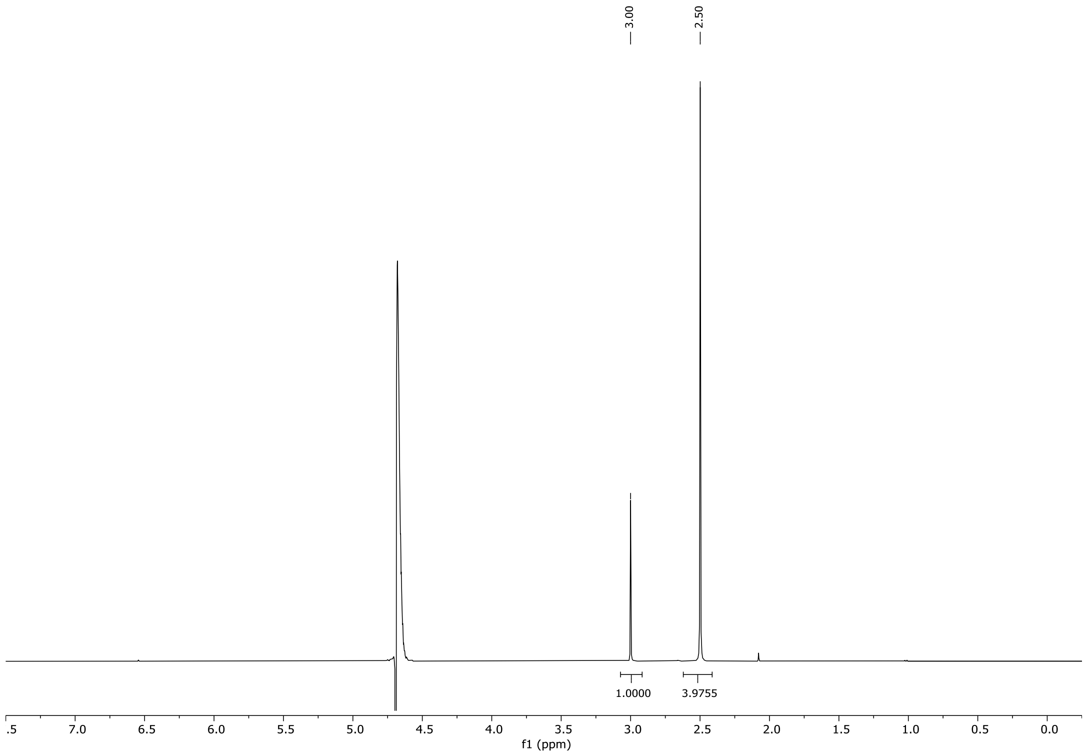

Figure S16. Representative ^{1}H qNMR using $Pd(OH)_2/C$ as catalyst (Table S1 entry 16−18).

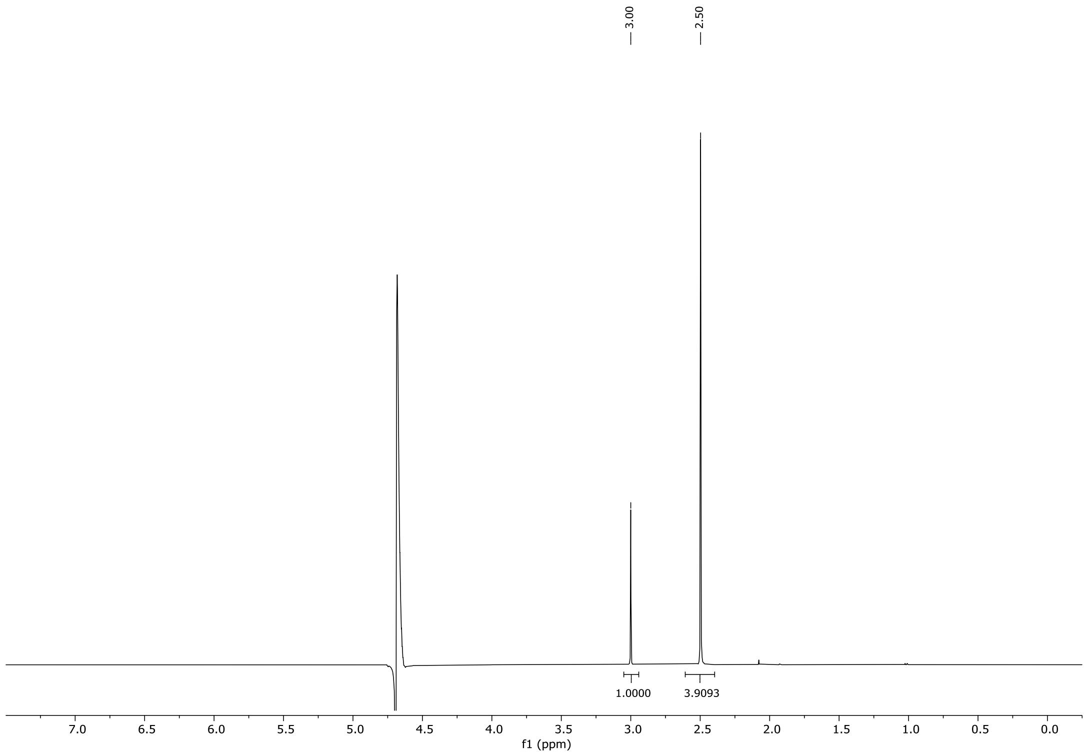

Figure S17. Representative ^{1}H qNMR using Pd/$BaSO_4$ as catalyst (Table S1 entry 19−21).

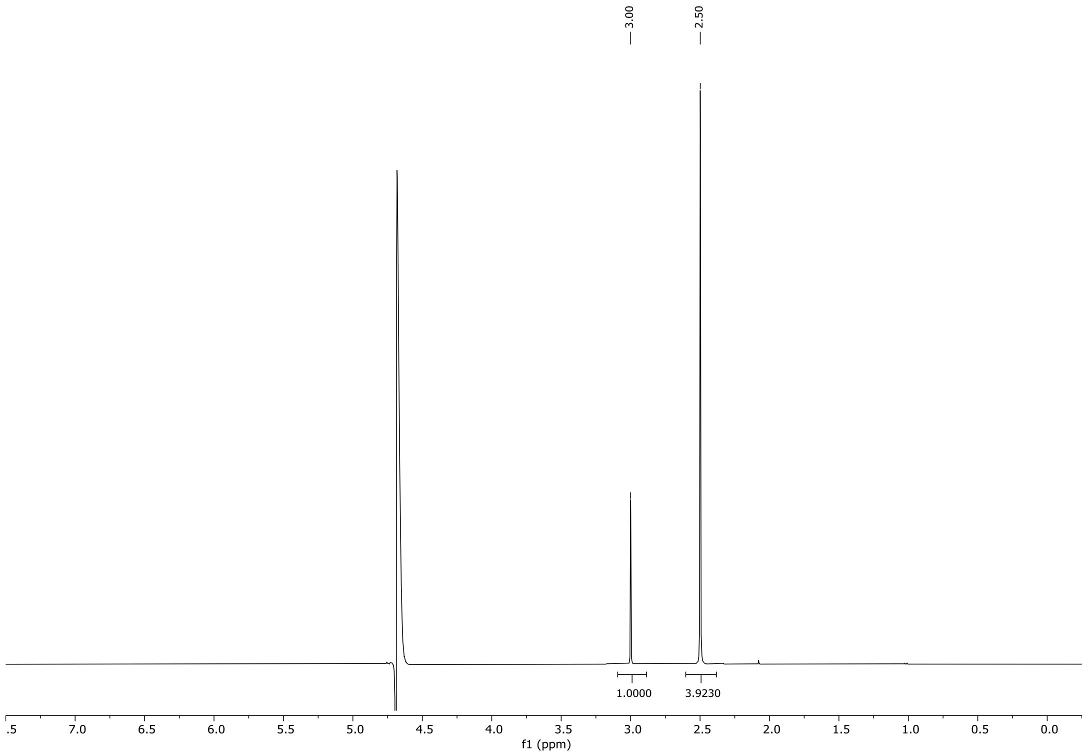

Figure S18. Representative ^{1}H qNMR using Pt/C as catalyst (Table S1 entry 22−24).

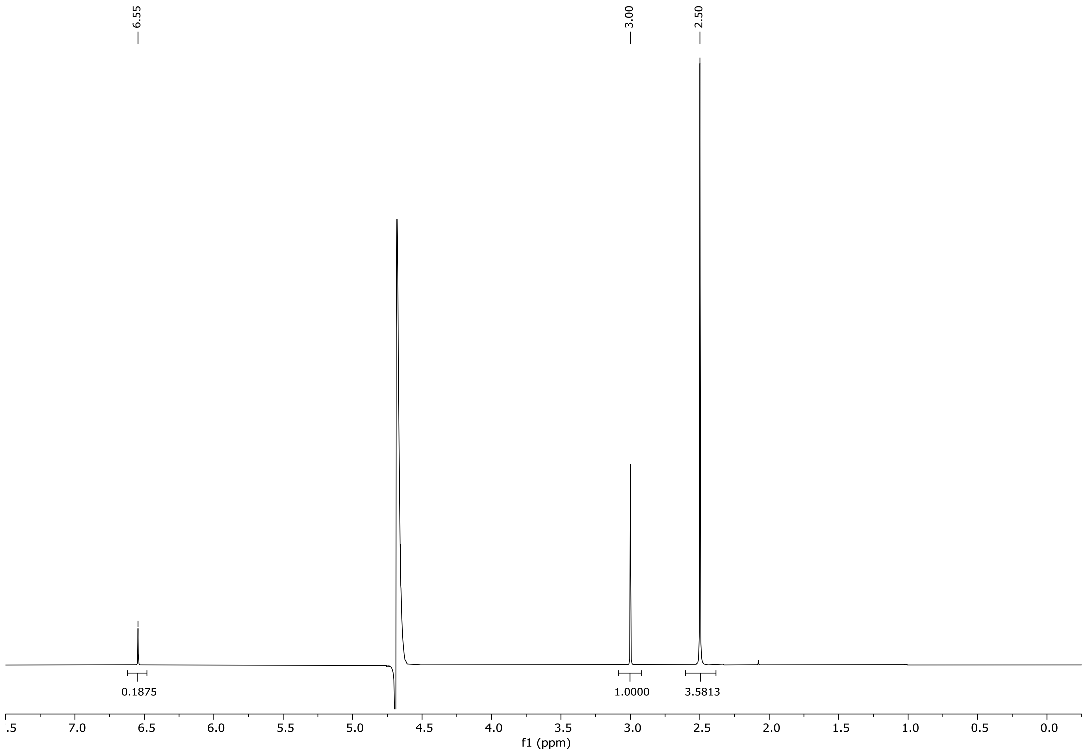

Figure S19. Representative ^{1}H qNMR using Pt/Al_2O_3 as catalyst (Table S1 entry 25−27).

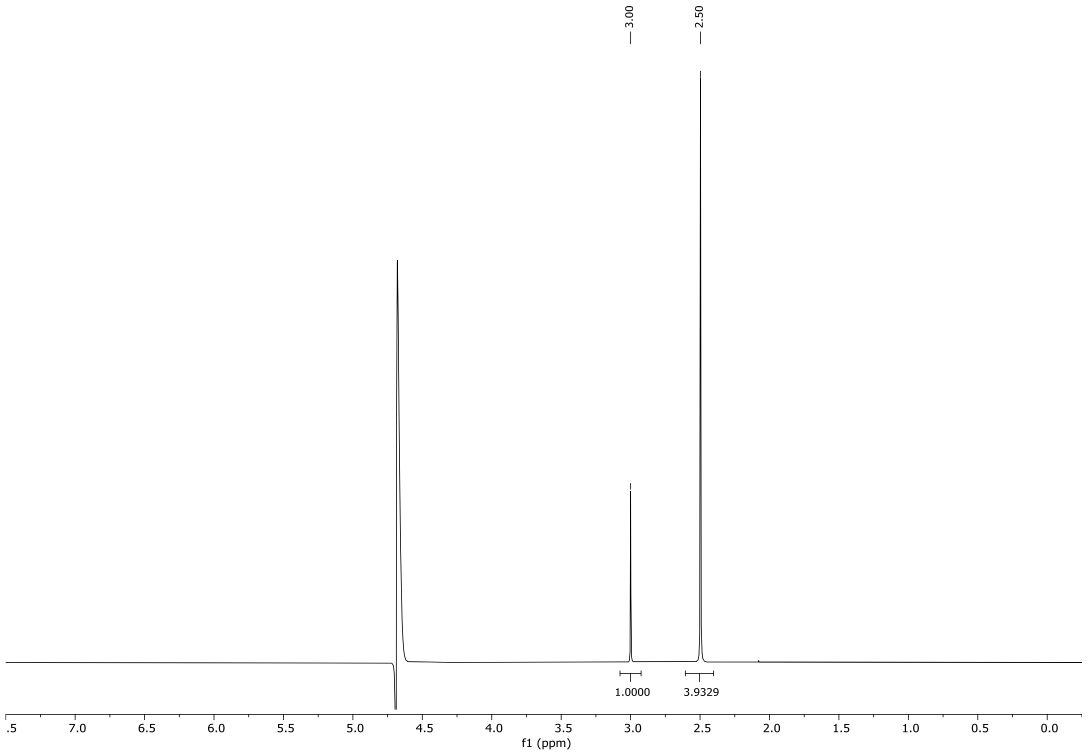

Figure S20. Representative ^{1}H qNMR using Ni/SiO_2-Al_2O_3 (10 mol%) as catalyst (Table S1 entry 28−30).

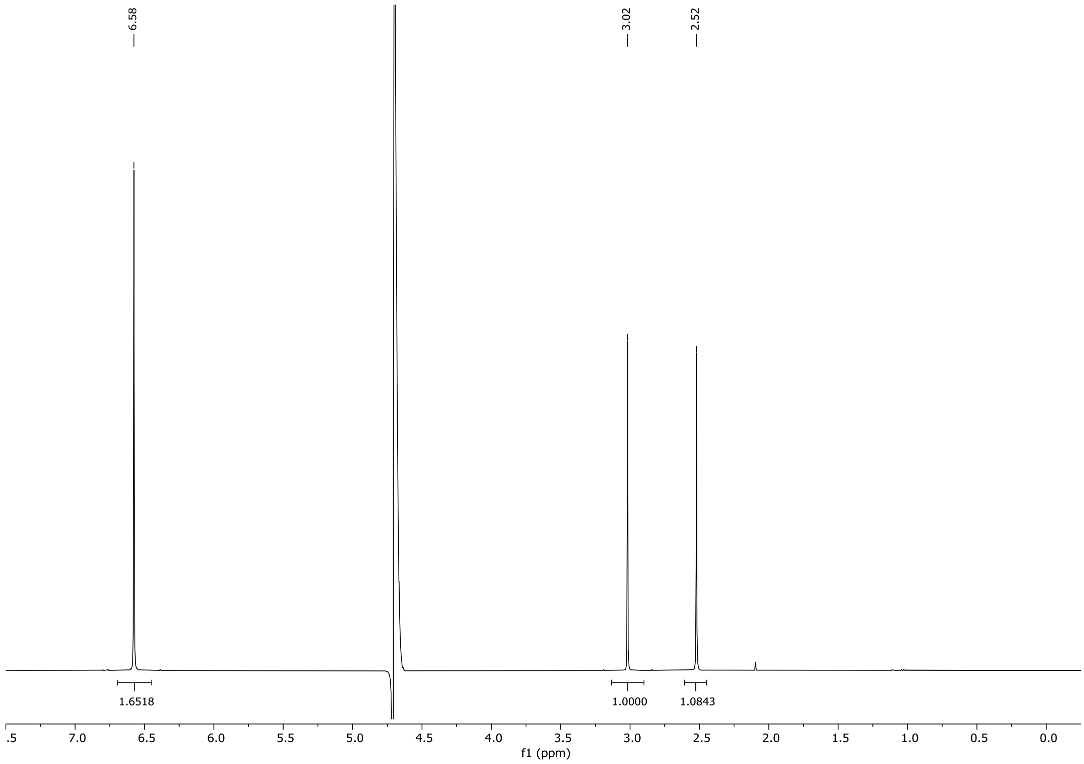

Figure S21. Representative ^{1}H qNMR using Ni/SiO_2-Al_2O_3 (1 mol%) as catalyst (Table S1 entry 31−33).

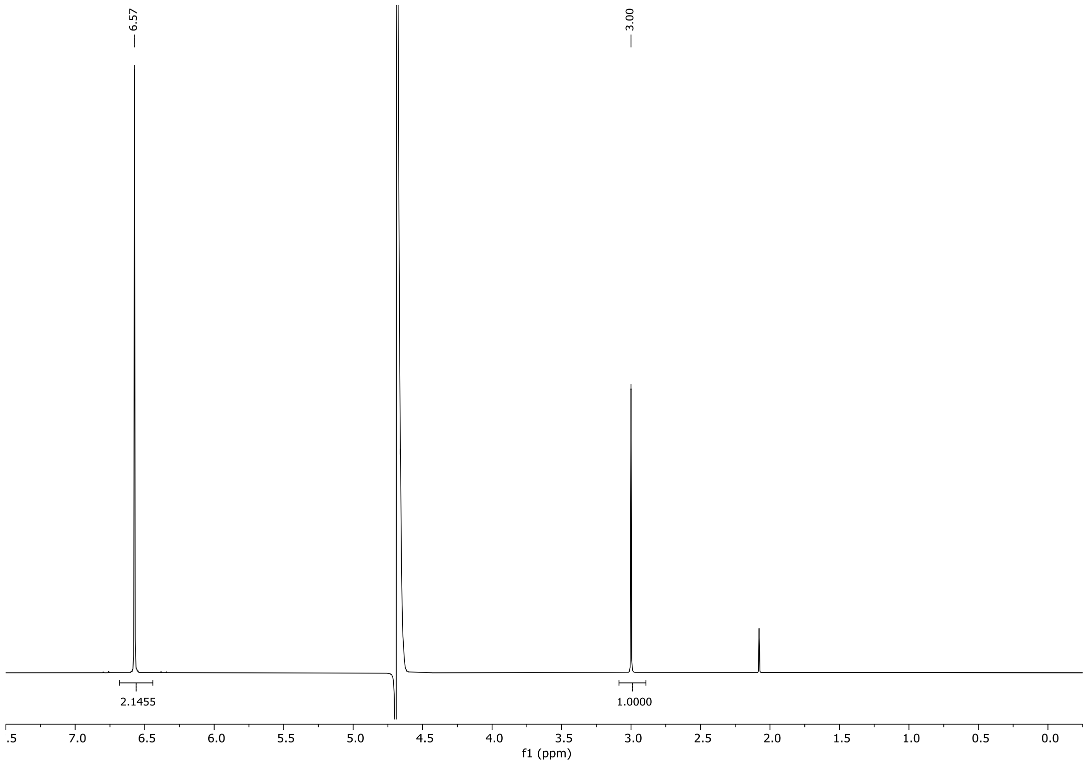

Figure S22. Representative ^{1}H qNMR without catalyst (Table S1 entry 34−36).

2.2 Catalyst screen for the reduction of oxaloacetic acid to malate

A disposable glass vial with PTFE/silicone-lined septum (see section 1.2 for more details) was charged with the metal catalyst (0.01 equiv., 1 mol%). $RhCl_3$, $PtCl_2$, and $PdCl_2$ were added from a stock solution (100 µL, 3 mM) and heterogeneous catalyst were added directly to the reaction vial. Then, oxaloacetic acid (0.03 mmol, 3.96 mg, 1 equiv.) was added from an aqueous stock solution (0.06 M adjusted to pH 7−8 with NaOH) and water was added to a total volume of 1 mL solvent. After purging the vial for 3 min with H_2, the vial equipped with an H_2 balloon was stirred at 22 °C for 18 h. Experiments were done in triplicates. qNMRs were acquired according to section 1.4.2. Mean values of yields and standard errors were calculated for each set of reactions.

Table S2. Catalyst screen - Reduction reaction of oxaloacetate to malate.

			integrals relative to DMS (6H) set to 1.0000 at 3.0000 ppm						yield [%]					
**#**	**catalyst loading (equiv., mol%, mg)**	**conc. DMS [M]**	**oxalo-acetate (2H)**	**malate (2H)**	**succi-nate (4H)**	**pyruvate (3H)**	**lactate (3H)**	**acetate (3H)**	**oxalo-acetate**	**malate**	**succi-nate**	**pyruvate**	**lactate**	**acetate**
**1**	$RhCl_3$ (0.01 equiv., 1 mol%, 0.08 mg)	0.02554	0.1443	0.0999	0.0042	1.3790	0.0490	0.0219	9.2	6.4	0.1	58.7	2.1	0.9
**2**	$RhCl_3$ (0.01 equiv., 1 mol%, 0.08 mg)	0.02554	0.2044	0.1259	0.0048	1.3000	0.0778	0.0240	13.1	8.0	0.2	55.3	3.3	1.0
**3**	$RhCl_3$ (0.01 equiv., 1 mol%, 0.08 mg)	0.02554	0.2130	0.0729	0.0016	1.3380	0.0532	0.0190	13.6	4.7	0.1	57.0	2.3	0.8
									12.0 ± 1.4	6.4 ± 1.0	0.1 ± 0	57.0 ± 1.0	2.6 ± 0.4	0.9 ± 0.1
**4**[a]	$PtCl_2$ (0.01 equiv., 1 mol%, 0.08 mg)	0.02221	0.2611	0.0293[b]	0	1.5854	0.0572	0.1254	29.0	3.3	0	58.7	2.1	4.6
**5**[a]	$PtCl_2$ (0.01 equiv., 1 mol%, 0.08 mg)	0.02221	0.1998	0.0190[b]	0	1.9013	0.0464	0.0927	22.2	2.1	0	70.4	1.7	3.4
**6**[a]	$PtCl_2$ (0.01 equiv., 1 mol%, 0.08 mg)	0.02221	0.2007	0.0210[b]	0	1.9764	0.0509	0.0327	22.3	2.3	0	73.2	1.9	1.2
									24.5 ± 2.3	2.6 ± 0.4	0 ± 0	67.4 ± 4.4	1.9 ± 0.1	3.1 ± 1.0
**7**[c]	$PdCl_2$ (0.01 equiv., 1 mol%, 0.05 mg)	0.02554	0.3043	0.0212[b]	0	1.4503	0.0095	0.0319	19.4	2.7	0	61.7	0.4	1.4
**8**[c]	$PdCl_2$ (0.01 equiv., 1 mol%, 0.05 mg)	0.02554	0.2682	0.0351[b]	0	1.4467	0.0088	0.0578	17.1	4.5	0	61.6	0.4	2.5
**9**[c]	$PdCl_2$ (0.01 equiv., 1 mol%, 0.05 mg)	0.02554	0.2722	0.0208[b]	0	1.3989	0.0070	0.0782	17.4	2.7	0	59.5	0.3	3.3
									18.0 ± 0.7	3.3 ± 0.6	0 ± 0	61.0 ± 0.7	0.4 ± 0	2.4 ± 0.6
**10**	Rh/Al_2O_3 (0.01 equiv., 1 mol%, 0.62 mg)	0.02221	0	1.7097	0	0.0128	0.2941	0.0228	0	94.9	0	0.5	10.9	0.8
**11**	Rh/Al_2O_3 (0.01 equiv., 1 mol%, 0.62 mg)	0.02221	0	1.6455	0	0.0388	0.2375	0.0222	0	91.4	0	1.4	8.8	0.8
**12**	Rh/Al_2O_3 (0.01 equiv., 1 mol%, 0.62 mg)	0.02221	0	1.6162	0	0.0104	0.2521	0.0204	0	89.7	0	0.4	9.3	0.8
									0 ± 0	92.0 ± 1.5	0 ± 0	0.8 ± 0.3	9.7 ± 0.6	0.8 ± 0

			integrals relative to DMS (6H) set to 1.0000 at 3.0000 ppm						yield [%]					
**#**	**catalyst loading (equiv., mol%, mg)**	**conc. DMS [M]**	**oxalo-acetate (2H)**	**malate (2H)**	**succi-nate (4H)**	**pyruvate (3H)**	**lactate (3H)**	**acetate (3H)**	**oxalo-acetate**	**malate**	**succi-nate**	**pyruvate**	**lactate**	**acetate**
**13**	Pd/C (0.01 equiv., 1 mol%, 0.32 mg)	0.02554	0.0236	0.0690	0	1.8097	0.0608	0.0205	1.5	4.4	0	77.0	2.6	0.9
**14**	Pd/C (0.01 equiv., 1 mol%, 0.32 mg)	0.02554	0.0539	0.0523	0	1.7674	0.0271	0.0180	3.4	3.3	0	75.2	1.2	0.8
**15**	Pd/C (0.01 equiv., 1 mol%, 0.32 mg)	0.02554	0.0529	0.0387	0.0020	1.7924	0.0188	0.0257	3.4	2.5	0.1	76.3	0.8	1.1
									2.8 ± 0.6	3.4 ± 0.6	0 ± 0	76.2 ± 0.5	1.5 ± 0.5	0.9 ± 0.1
**16**[d]	$Pd(OH)_2/C$ (0.01 equiv., 1 mol%, 0.16 mg)	0.02554	0.0317	0.1342	0	1.6258	0.0471	0.0529	2.0	8.6	0	69.2	2.0	2.3
**17**[d]	$Pd(OH)_2/C$ (0.01 equiv., 1 mol%, 0.16 mg)	0.02554	0.0766	0.0646	0	1.6585	0.0452	0.0153	4.9	4.1	0	70.6	1.9	0.7
**18**[d]	$Pd(OH)_2/C$ (0.01 equiv., 1 mol%, 0.16 mg)	0.02554	0.0988	0.0624	0	1.6268	0.0256	0.0205	6.3	4.0	0	69.2	1.1	0.9
									4.4 ± 1.3	5.6 ± 1.5	0 ± 0	69.7 ± 0.5	1.7 ± 0.3	1.3 ± 0.5
**19**[e]	$Pd/BaSO_4$ (0.01 equiv., 1 mol%, 0.64 mg)	0.02554	0.1526	0.0776	0	0.8835	0.0058	0.2056	9.7	5.0	0	37.6	0.2	8.8
**20**[e]	$Pd/BaSO_4$ (0.01 equiv., 1 mol%, 0.64 mg)	0.02554	0.1706	0.0577	0	0.8166	0.0067	0.2316	10.9	3.7	0	34.8	0.3	9.9
**21**[e]	$Pd/BaSO_4$ (0.01 equiv., 1 mol%, 0.64 mg)	0.02554	0.2103	0.1020	0	0.8991	0.0095	0.2202	13.4	6.5	0	38.3	0.4	9.4
									11.4 ± 1.1	5.1 ± 0.8	0 ± 0	36.9 ± 1.1	0.3 ± 0	9.3 ± 0.3
**22**	Pt/C (0.01 equiv., 1 mol%, 1.72 mg)	0.02554	0.0708	0.2304	0	1.0749	0.3643	0.0212	4.5	14.7	0	45.8	15.5	0.9
**23**	Pt/C (0.01 equiv., 1 mol%, 1.72 mg)	0.02554	0.1376	0.2297	0.0023	0.7024	0.3102	0.0614	8.8	14.7	0.1	29.9	13.2	2.6
**24**	Pt/C (0.01 equiv., 1 mol%, 1.72 mg)	0.02554	0.1436	0.2551	0.0027	0.7984	0.4023	0.0200	9.2	16.3	0.1	34.0	17.1	0.9
									7.5 ± 1.5	15.2 ± 0.5	0.1 ± 0	36.5 ± 4.8	15.3 ± 1.1	1.5 ± 0.6
**25**	Pt/Al_2O_3 (0.01 equiv., 1 mol%, 5.85 mg)	0.02554	0	0.4113	0.0017	1.2318	0.1343	0.0155	0	26.3	0.1	52.4	5.7	0.7
**26**	Pt/Al_2O_3 (0.01 equiv., 1 mol%, 5.85 mg)	0.02554	0	0.3601	0	1.3030	0.1102	0.0430	0	23.0	0	55.5	4.7	1.8
**27**	Pt/Al_2O_3 (0.01 equiv., 1 mol%, 5.85 mg)	0.02554	0	0.4182	0	1.1690	0.1239	0.0426	0	26.7	0	49.8	5.3	1.8
									0 ± 0	25.3 ± 1.2	0 ± 0	52.6 ±1.6	5.2 ± 0.3	1.4 ± 0.4

			integrals relative to DMS (6H) set to 1.0000 at 3.0000 ppm						yield [%]					
**#**	**catalyst loading (equiv., mol%, mg)**	**conc. DMS [M]**	**oxalo-acetate (2H)**	**malate (2H)**	**succi-nate (4H)**	**pyruvate (3H)**	**lactate (3H)**	**acetate (3H)**	**oxalo-acetate**	**malate**	**succi-nate**	**pyruvate**	**lactate**	**acetate**
**28**[f]	Ni/SiO_2-Al_2O_3 (0.1 equiv., 10 mol%, 0.26 mg)	0.02221	0	0.4662	0	1.4781	0.1894	0.0271	0	25.9	0	54.7	7.0	1.0
**29**[f]	Ni/SiO_2-Al_2O_3 (0.1 equiv., 10 mol%, 0.26 mg)	0.02221	0	0.3358	0	1.7368	0.0897	0.0274	0	18.6	0	64.3	3.3	1.0
**30**[f]	Ni/SiO_2-Al_2O_3 (0.1 equiv., 10 mol%, 0.26 mg)	0.02221	0	0.4206	0	1.5963	0.1456	0.0261	0	23.4	0	59.1	5.4	1.0
									0 ± 0	22.6 ± 2.1	0 ± 0	59.4 ± 2.8	5.2 ± 1.1	1.0 ± 0
**31**	none	0.02554	0.2702	0	0	1.5821	0	0.0132	17.3	0	0	67.3	0	0.6
**32**	none	0.02554	0.2553	0	0	1.6054	0	0.0165	16.3	0	0	68.3	0	0.7
**33**	none	0.02554	0.2119	0	0	1.7527	0	0.0121	13.5	0	0	74.6	0	0.5
									15.7 ± 1.1	0 ± 0	0 ± 0	70.1 ± 2.3	0 ± 0	0.6 ± 0.1

[a] malonate 3.7 ± 1.7% [b] quantification of malate by integration of one half of the qd from 2.60−2.66 ppm corresponding to one proton [c] malonate 2.2 ± 0.9% [d] malonate: 0.7 ± 0.7% [e] malonate: 11.5 ± 0.4%, [f] standard reaction conditions (see Table S3)

Metals without reduction reactivity under the above-mentioned reaction conditions: Rh/C, Ru/Al_2O_3, $NiCl_2$, NiO, $CoCl_2$, Co-Mo/Al_2O_3 (cobalt oxide 3.4−4.5%, molybdenum oxide 11.5−14.5% on alumina), $CoAl_2O_4$ (39.69%), $FeCl_2$.

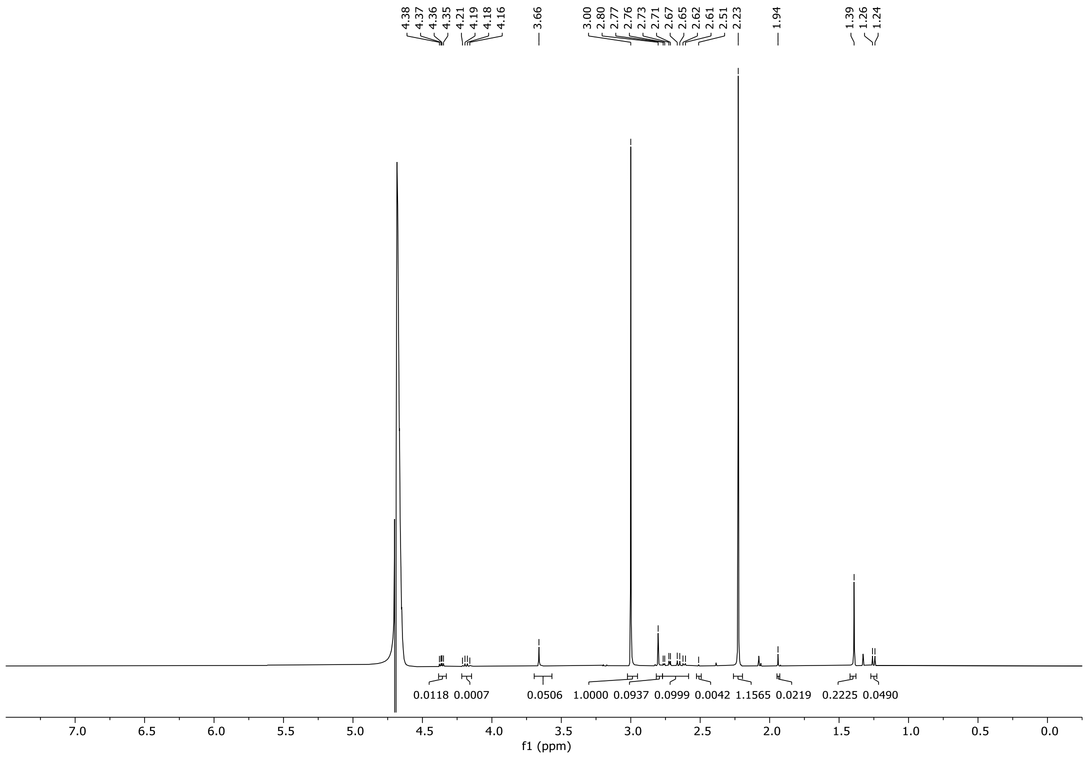

Figure S23. Representative ^{1}H qNMR using $RhCl_3$ as catalyst (Table S2 entry 1−3).

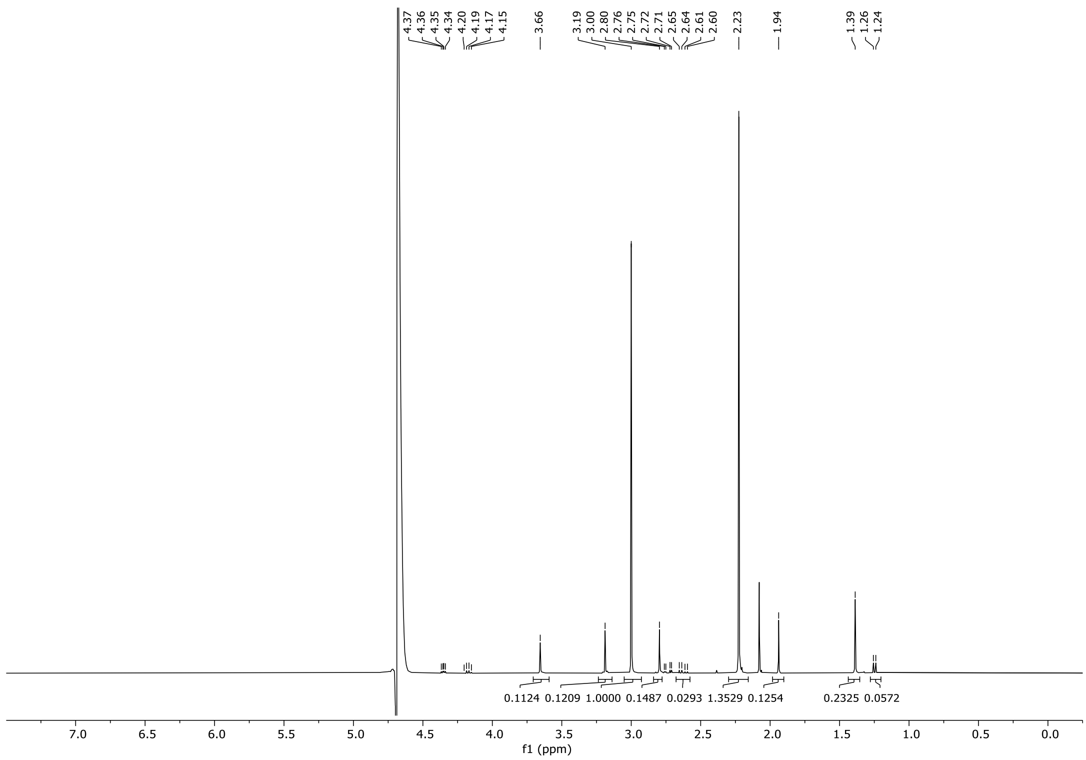

Figure S24. Representative ^{1}H qNMR using $PtCl_2$ as catalyst (Table S2 entry 4−6).

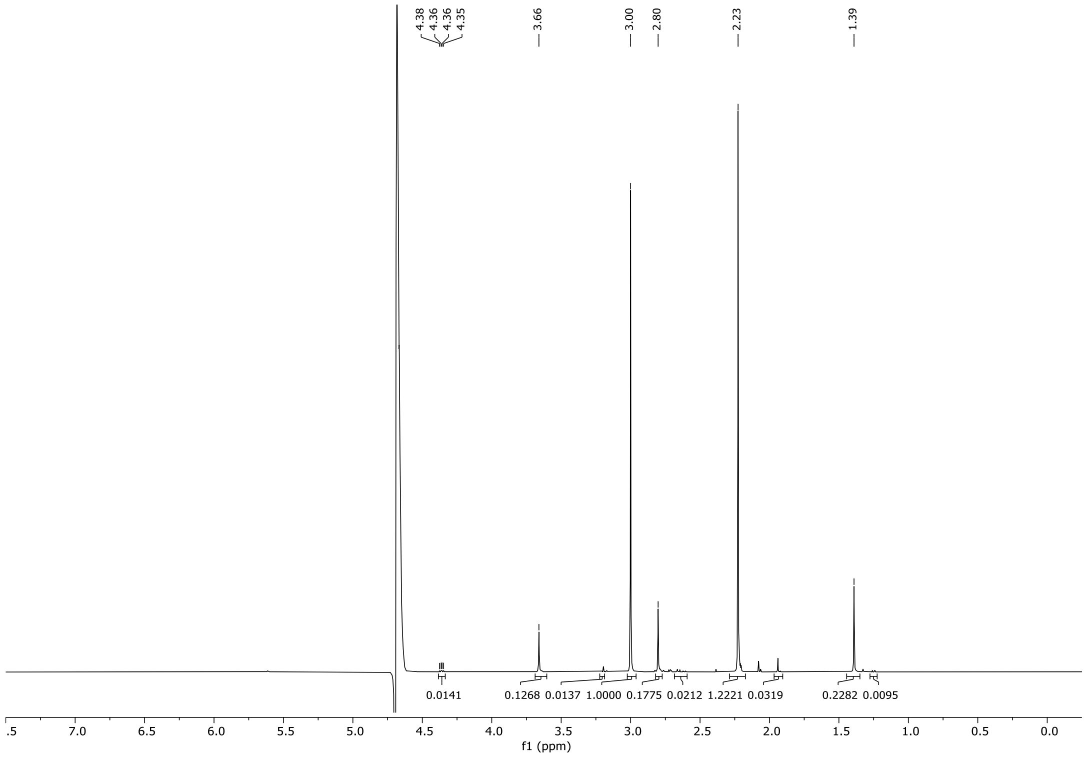

Figure S25. Representative ^{1}H qNMR using $PdCl_2$ as catalyst (Table S2 entry 7−9).

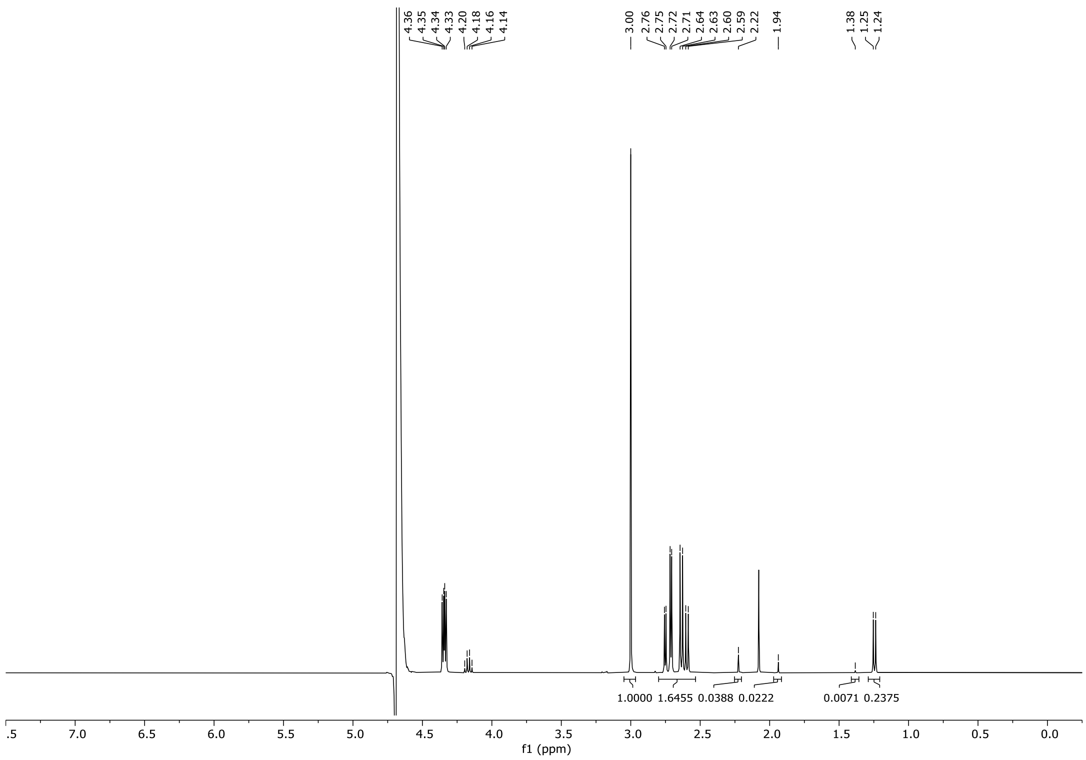

Figure S26. Representative ^{1}H qNMR using Rh/Al_2O_3 as catalyst (Table S2 entry 10−12).

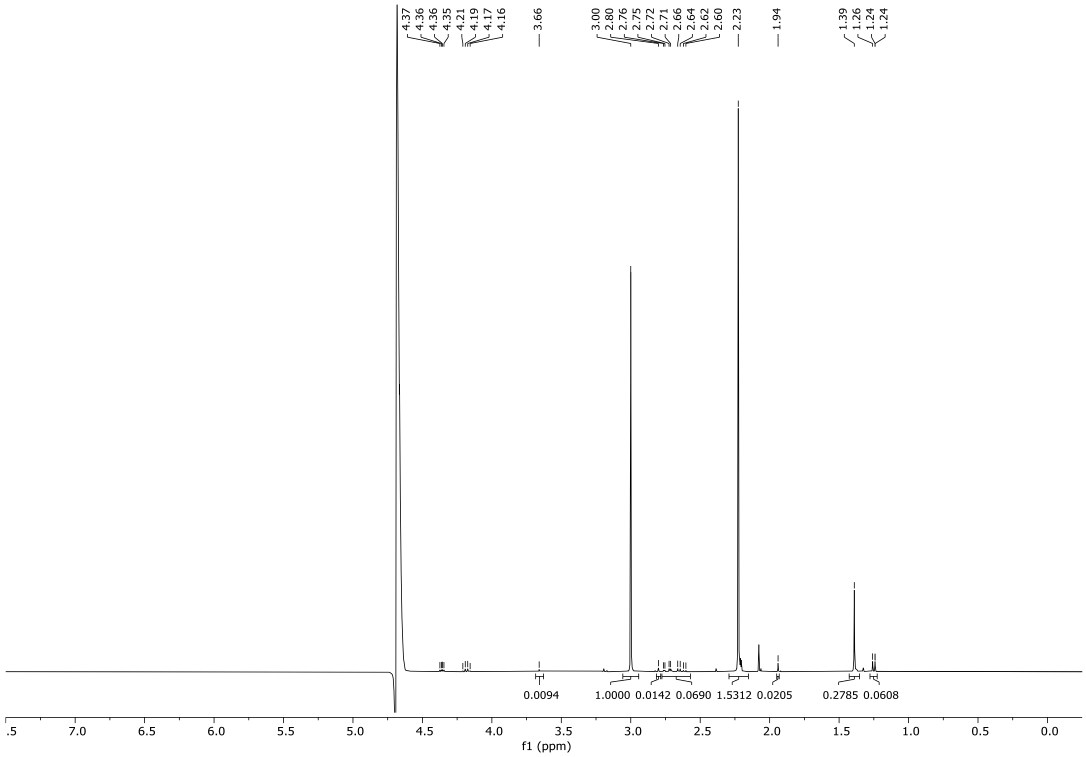

Figure S27. Representative ^{1}H qNMR using Pd/C as catalyst (Table S2 entry 13−15).

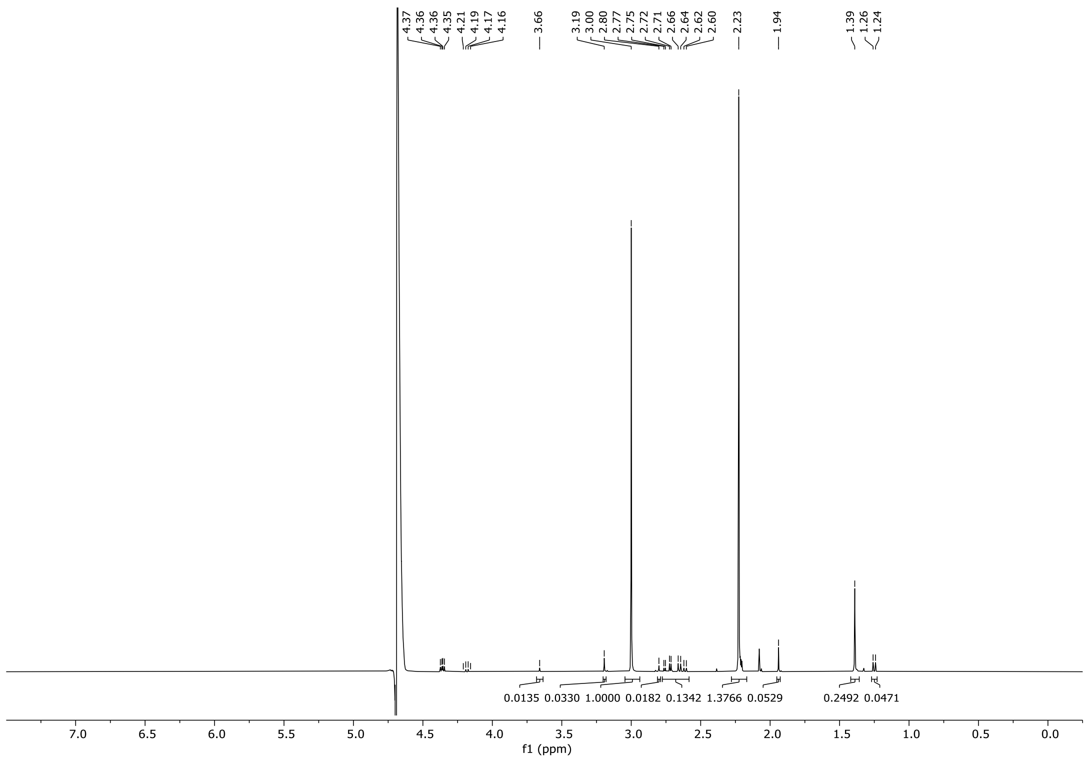

Figure S28. Representative ^{1}H qNMR using $Pd(OH)_2/C$ as catalyst (Table S2 entry 16−18).

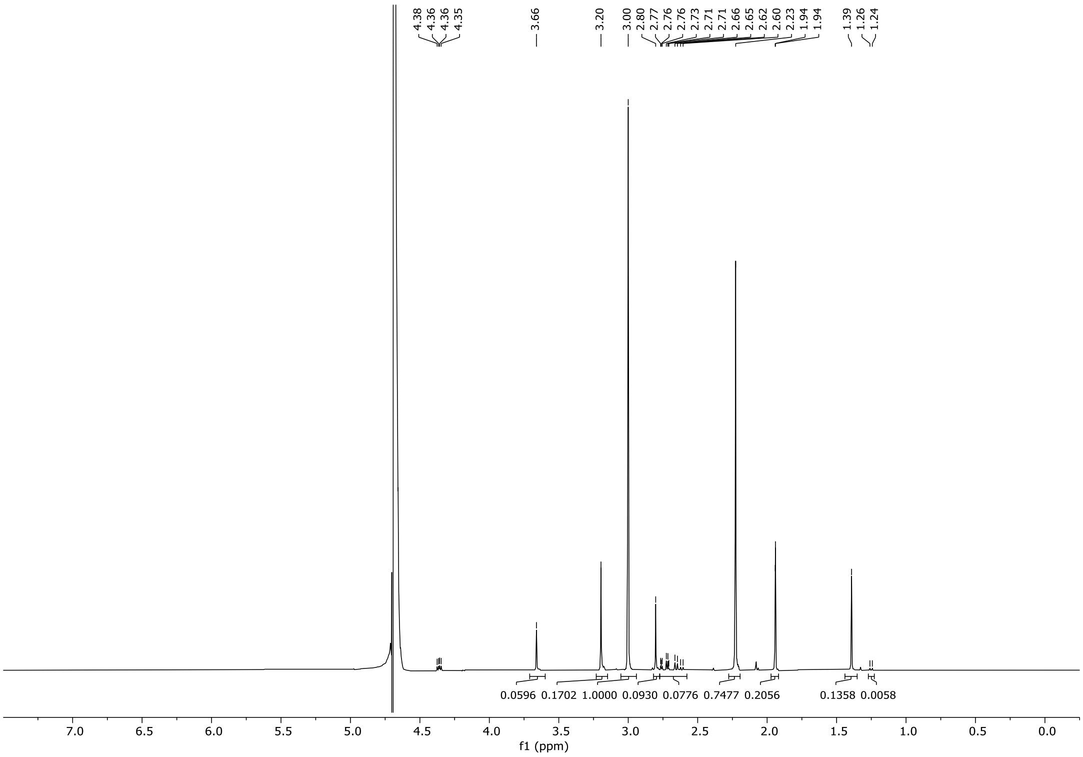

Figure S29. Representative ^{1}H qNMR using Pd/$BaSO_4$ as catalyst (Table S2 entry 19−21).

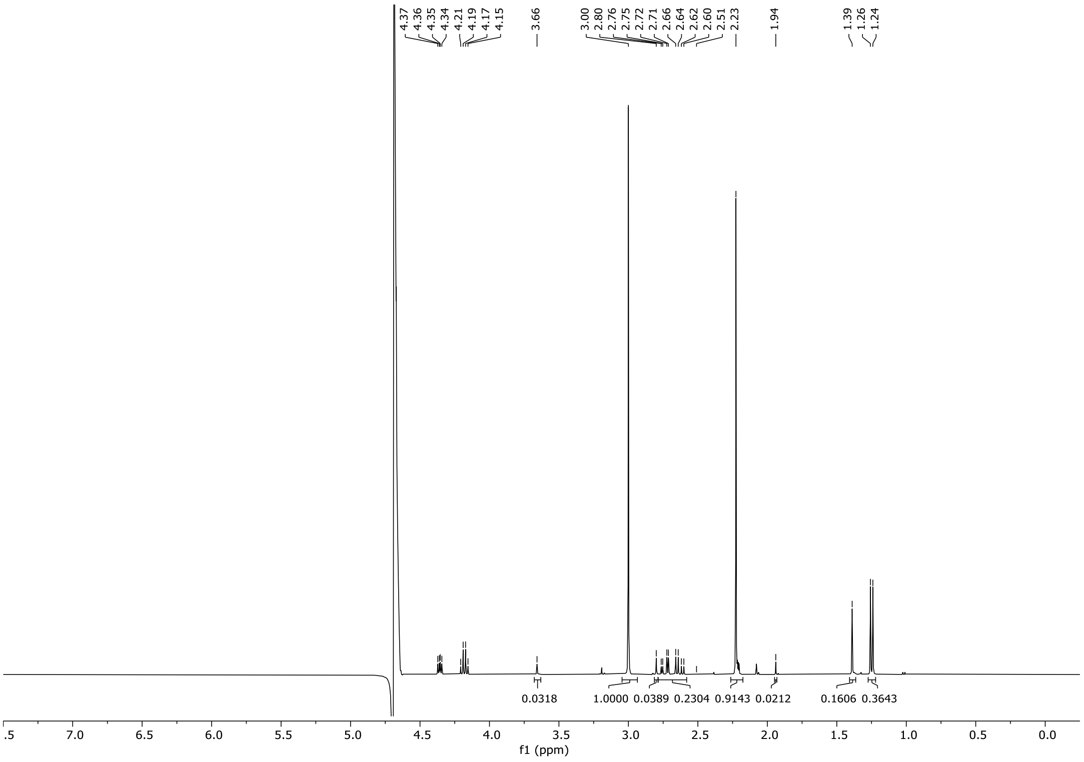

Figure S30. Representative ^{1}H qNMR using Pt/C as catalyst (Table S2 entry 22−24).

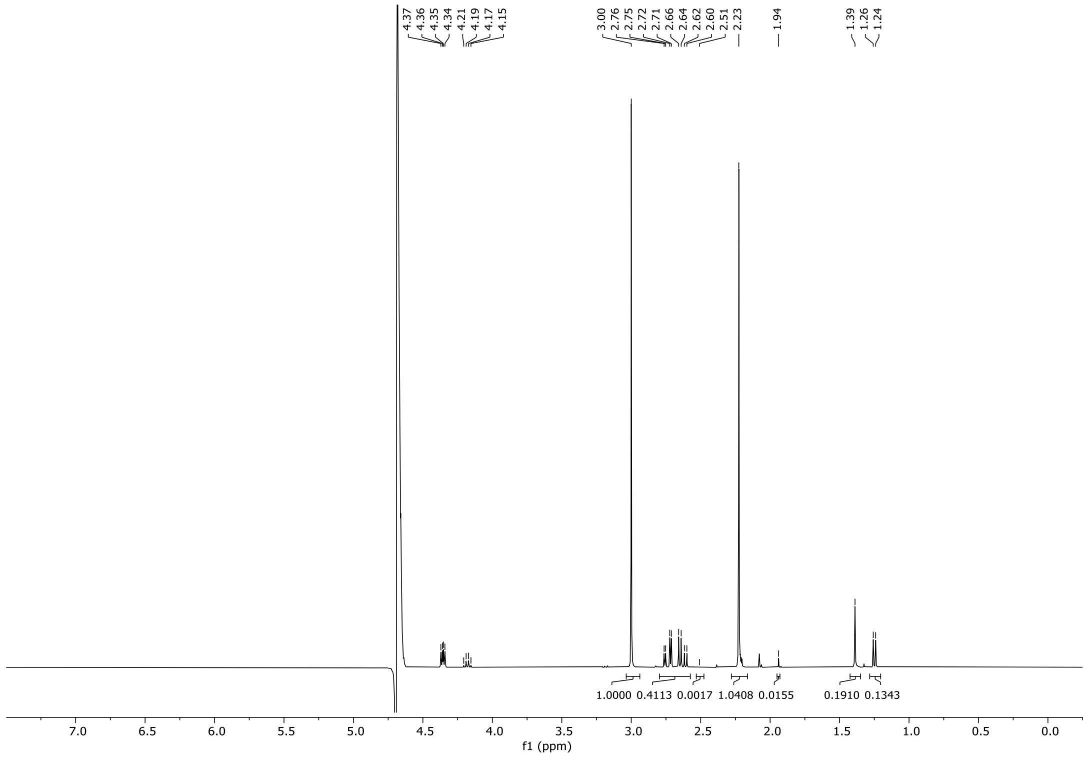

Figure S31. Representative ^{1}H qNMR using Pt/Al_2O_3 as catalyst (Table S2 entry 25−27).

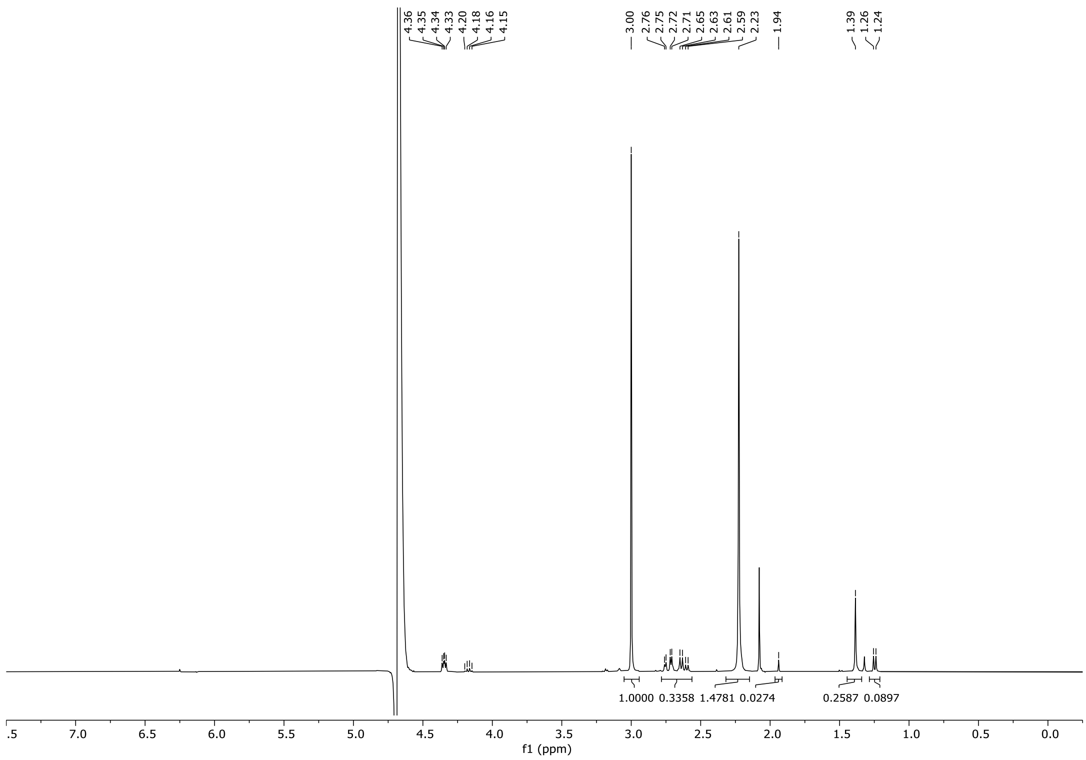

Figure S32. Representative ^{1}H qNMR using Ni/SiO_2-Al_2O_3 as catalyst (Table S2 entry 28−30).

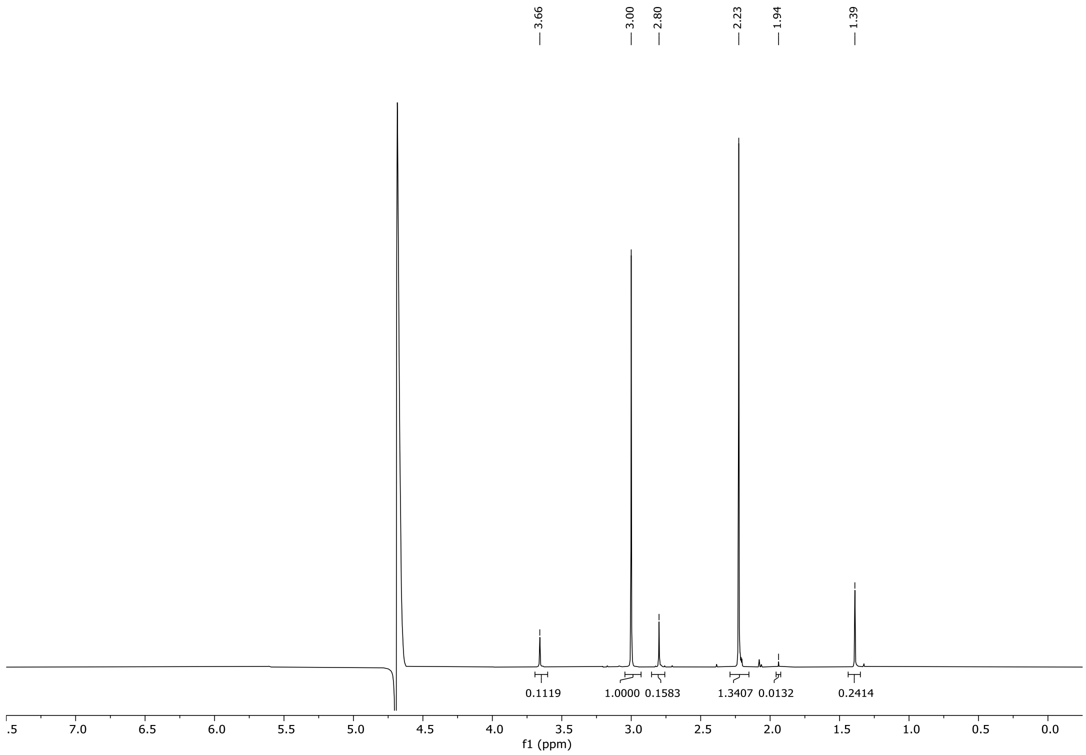

Figure S33. Representative ^{1}H qNMR without catalyst (Table S2 entry 31−33).

2.3 Competition experiment

A flask with septum and PTFE-stir bar was charged with metal catalyst (Rh/Al_2O_3, 0.01 equiv., 1 mol%, 3.1 mg **or** Ni/SiO_2-Al_2O_3, 0.1 equiv., 10 mol%, 1.3 mg). Then, an equimolar aqueous solution of oxaloacetic acid (0.03 mmol, 19.8 mg, 1 equiv.), sodium pyruvate (0.03 mmol, 16.5 mg, 1 equiv.), and sodium fumarate dibasic (0.03 mmol, 24.0 mg, 1 equiv.) were added from a stock solution whose pH was previously adjusted to pH 7−8 with NaOH. Water was added to a total volume of 5 mL. The flasks were purged for 5 min with H_2, equipped with an H_2 balloon and stirred at 22 °C. Aliquots of 300 μL were collected at t = 0, 15, 30, 60 min and NMR samples were prepared and acquired according to section 1.4.2.

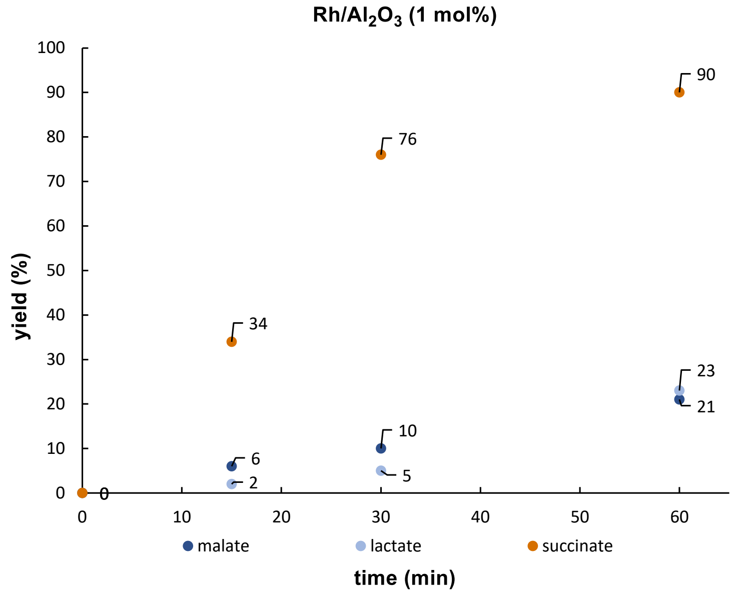

Figure S34. Competition experiment with Rh/Al_2O_3 (1 mol%).

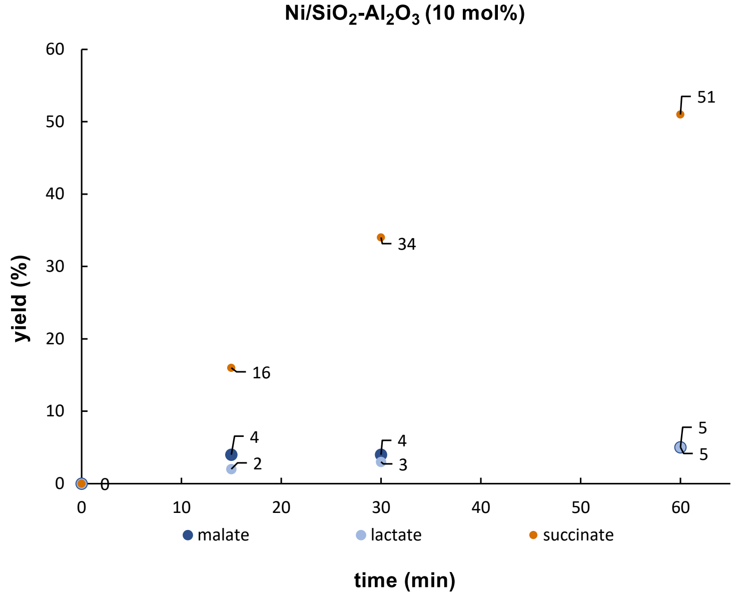

Figure S35. Competition experiment with Ni/SiO_2-Al_2O_3 (10 mol%).

Selectivity at t = 15 min for Rh/Al_2O_3: oxaloacetate vs pyruvate = 3:1
Selectivity at t = 15 min for Ni/SiO_2-Al_2O_3: oxaloacetate vs pyruvate = 2:1
For both catalysts the selectivity is in general: fumarate > oxaloacetate > pyruvate

2.4 Variation of reaction parameters for Rh/Al_2O_3 and Ni/SiO_2-Al_2O_3

A disposable glass vial with PTFE/silicone-lined septum (see section 1.2 for more details) was charged with the metal catalyst (Rh/Al_2O_3, 0.01 equiv., 1 mol%, 0.62 mg **or** Ni/SiO_2-Al_2O_3, 0.1 equiv., 10 mol%, 0.26 mg). Then, oxaloacetic acid (0.03 mmol, 3.96 mg, 1 equiv.) was added from an aqueous stock solution (0.06 M in water; adjusted to pH 7−8 with NaOH) and water was added to a total volume of 1 mL solvent. After purging the vial for 3 min with H_2, the vial was equipped with an H_2 balloon and was stirred at 22 °C for 18 h. Experiments were done in triplicates if not otherwise noted. qNMRs were acquired according to section 1.4.2. Mean values of yields and standard errors were calculated for each set of reactions.

Reaction parameters such as H_2 pressure (section 2.4.2), temperature (section 2.4.3), pH (section 2.4.4), and catalyst loading (section 2.4.5) were varied starting from standard conditions.

2.4.1 Standard reaction conditions

Table S3. Reaction conditions: oxaloacetic acid (30 mM), Rh/Al_2O_3 (1 mol%) or Ni/SiO_2-Al_2O_3 (10 mol%), H_2 (1 atm), pH 7−8, 22 °C, 18 h.

			integrals relative to DMS (6H) set to 1.0000 at 3.0000 ppm						yield [%]					
**#**	**catalyst loading (equiv., mol%, mg)**	**conc. DMS [M]**	**oxalo-acetate (2H)**	**malate (2H)**	**succi-nate (4H)**	**pyruvate (3H)**	**lactate (3H)**	**acetate (3H)**	**oxalo-acetate**	**malate**	**succi-nate**	**pyruvate**	**lactate**	**acetate**
**1**	Rh/Al_2O_3 (0.01 equiv., 1 mol%, 0.62 mg)	0.02221	0	1.7097	0	0.0128	0.2941	0.0228	0	94.9	0	0.5	10.9	0.8
**2**	Rh/Al_2O_3 (0.01 equiv., 1 mol%, 0.62 mg)	0.02221	0	1.6455	0	0.0388	0.2375	0.0222	0	91.4	0	1.4	8.8	0.8
**3**	Rh/Al_2O_3 (0.01 equiv., 1 mol%, 0.62 mg)	0.02221	0	1.6162	0	0.0104	0.2521	0.0204	0	89.7	0	0.4	9.3	0.8
									0 ± 0	92.0 ± 1.5	0 ± 0	0.8 ± 0.3	9.7 ± 0.6	0.8 ± 0
**4**	Ni/SiO_2-Al_2O_3 (0.1 equiv., 10 mol%, 0.26 mg)	0.02221	0	0.4662	0	1.4781	0.1894	0.0271	0	25.9	0	54.7	7.0	1.0
**5**	Ni/SiO_2-Al_2O_3 (0.1 equiv., 10 mol%, 0.26 mg)	0.02221	0	0.3358	0	1.7368	0.0897	0.0274	0	18.6	0	64.3	3.3	1.0
**6**	Ni/SiO_2-Al_2O_3 (0.1 equiv., 10 mol%, 0.26 mg)	0.02221	0	0.4206	0	1.5963	0.1456	0.0261	0	23.4	0	59.1	5.4	1.0
									0 ± 0	22.6 ± 2.1	0 ± 0	59.4 ± 2.8	5.2 ± 1.1	1.0 ± 0
**7**	none	0.02554	0.2702	0	0	1.5821	0	0.0132	17.3	0	0	67.3	0	0.6
**8**	none	0.02554	0.2553	0	0	1.6054	0	0.0165	16.3	0	0	68.3	0	0.7
**9**	none	0.02554	0.2119	0	0	1.7527	0	0.0121	13.5	0	0	74.6	0	0.5
									15.7 ± 1.1	0 ± 0	0 ± 0	70.1 ± 2.3	0 ± 0	0.6 ± 0.1

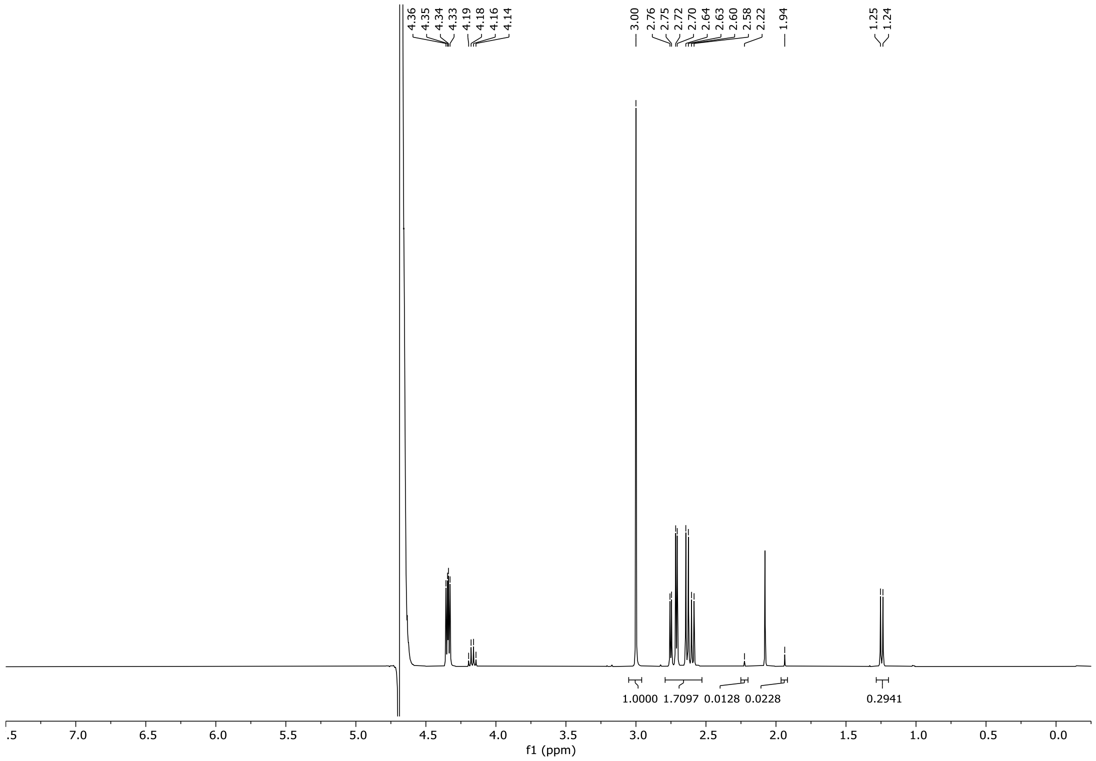

Figure S36. Representative ^{1}H qNMR using Rh/Al_2O_3 as catalyst (Table S3 entry 1−3).

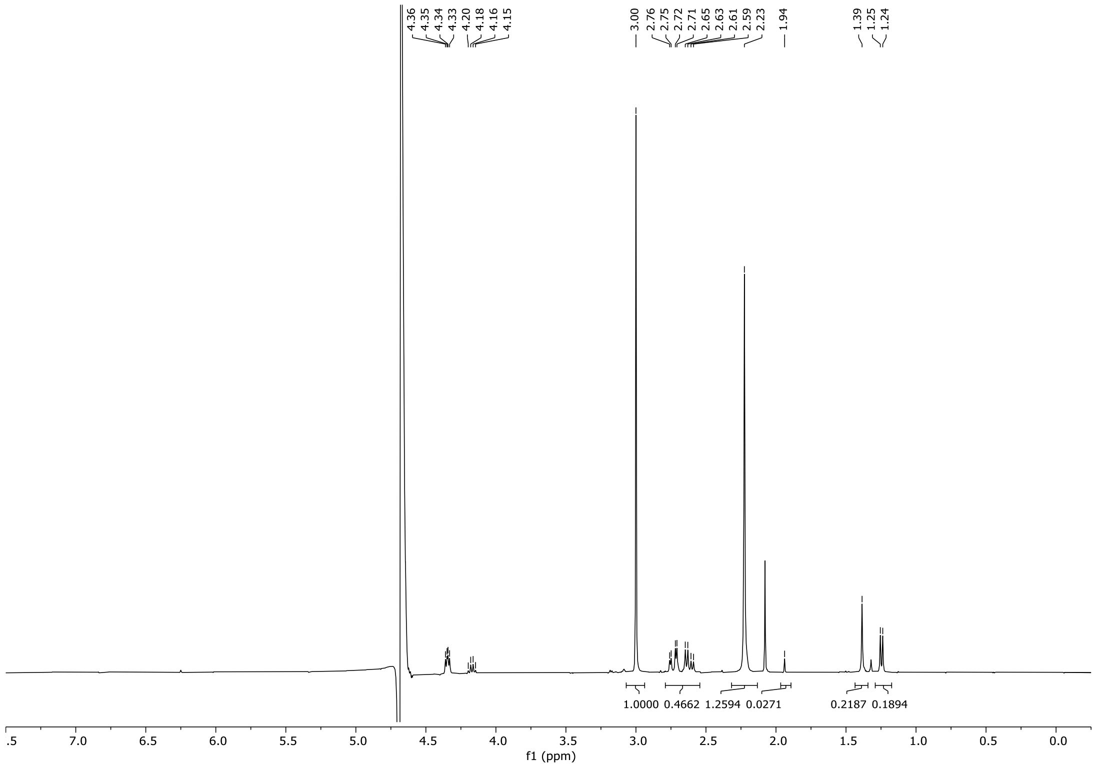

Figure S37. Representative ^{1}H qNMR using Ni/SiO_2-Al_2O_3 as catalyst (Table S3 entry 4−6).

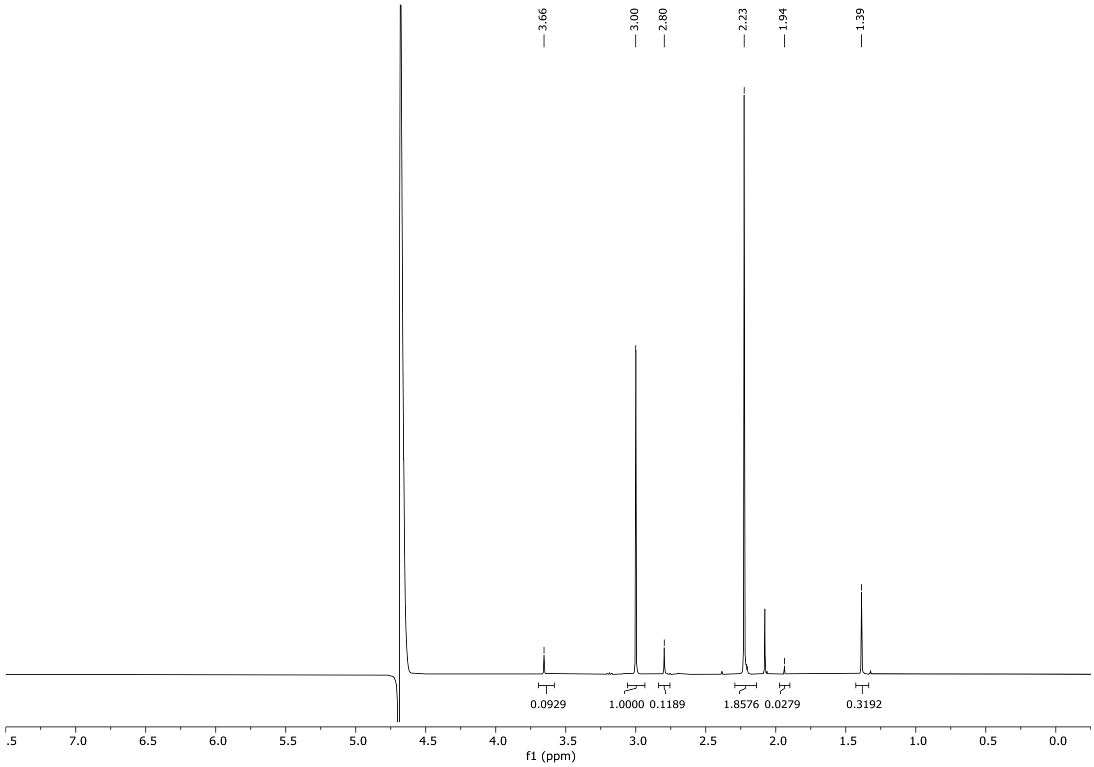

Figure S38. Representative ^{1}H qNMR without catalyst (Table S3 entry 7−9).

2.4.2 Variation of H_2 pressure

Table S4. Reaction conditions: oxaloacetic acid (30 mM), Rh/Al_2O_3 (1 mol%) or Ni/SiO_2-Al_2O_3 (10 mol%), H_2 (5 **or** 10 bar), pH 7−8, 22 °C, 18 h.

			integrals relative to DMS (6H) set to 1.0000 at 3.0000 ppm						yield [%]					
**#**	**catalyst loading (equiv., mol%, mg)**	**conc. DMS [M]**	**oxalo-acetate (2H)**	**malate (2H)**	**succi-nate (4H)**	**pyruvate (3H)**	**lactate (3H)**	**acetate (3H)**	**oxalo-acetate**	**malate**	**succi-nate**	**pyruvate**	**lactate**	**acetate**
**5 bar**														
**1**	Rh/Al_2O_3 (0.01 equiv., 1 mol%, 0.62 mg)	0.02221	0	1.6291	0	0.0678	0.1295	0.0173	0	90.5	0	2.5	4.8	0.6
**2**	Rh/Al_2O_3 (0.01 equiv., 1 mol%, 0.62 mg)	0.02221	0	1.6543	0	0.0525	0.1265	0.0179	0	91.9	0	1.9	4.7	0.7
**3**	Rh/Al_2O_3 (0.01 equiv., 1 mol%, 0.62 mg)	0.02221	0	1.6390	0	0.0762	0.1286	0.0176	0	91.0	0	2.8	4.8	0.7
									0 ± 0	91.1 ± 0.4	0 ± 0	2.4 ± 0.3	4.7 ± 0	0.7 ± 0
**4**	Ni/SiO_2-Al_2O_3 (0.1 equiv., 10 mol%, 0.26 mg)	0.02221	0	0.8517	0	0.4301	0.7576	0.0226	0	47.3	0	15.9	28.0	0.8
**5**	Ni/SiO_2-Al_2O_3 (0.1 equiv., 10 mol%, 0.26 mg)	0.02221	0	0.8486	0	0.4472	0.7393	0.0202	0	47.1	0	16.6	27.4	0.7
**6**	Ni/SiO_2-Al_2O_3 (0.1 equiv., 10 mol%, 0.26 mg)	0.02221	0	0.7249	0	0.6274	0.6712	0.0188	0	40.3	0	23.2	24.8	0.7
									0 ± 0	44.9 ± 2.3	0 ± 0	18.6 ± 2.3	26.8 ± 1.0	0.8 ± 0
**7**	none	0.02221	0.0965	0	0	2.3360	0	0.0327	10.7	0	0	86.5	0	1.2
**8**	none	0.02221	0.0928	0	0	2.3672	0	0.0329	10.3	0	0	87.6	0	1.2
**9**	none	0.02221	0.0732	0	0	2.4057	0	0.0219	8.1	0	0	89.1	0	0.8
									9.7 ± 0.8	0 ± 0	0 ± 0	87.7 ± 0.7	0 ± 0	1.1 ± 0.1
**10 bar**														
**10**	Rh/Al_2O_3 (0.01 equiv., 1 mol%, 0.62 mg)	0.02337	0	1.5051	0	0.0082	0.0998	0.0198	0	87.9	0	0.3	3.9	0.8
**11**	Rh/Al_2O_3 (0.01 equiv., 1 mol%, 0.62 mg)	0.02337	0	1.3730	0	0.0138	0.0963	0.0179	0	80.2	0	0.5	3.8	0.7
**12**	Rh/Al_2O_3 (0.01 equiv., 1 mol%, 0.62 mg)	0.02337	0	1.5076	0	0.0198	0.1101	0.0229	0	88.1	0	0.8	4.3	0.9
									0 ± 0	85.4 ± 2.6	0 ± 0	0.5 ± 0.1	4.0 ± 0.2	0.8 ± 0.1
**13**	Ni/SiO_2-Al_2O_3 (0.1 equiv., 10 mol%, 0.26 mg)	0.02337	0	0.7595	0	0.5797	0.4992	0.0241	0	44.4	0	22.6	19.4	0.9
**14**	Ni/SiO_2-Al_2O_3 (0.1 equiv., 10 mol%, 0.26 mg)	0.02337	0	0.8530	0	0.4537	0.5081	0.0205	0	49.8	0	17.7	19.8	0.8
**15**	Ni/SiO_2-Al_2O_3 (0.1 equiv., 10 mol%, 0.26 mg)	0.02337	0	0.8191	0	0.4531	0.5351	0.0240	0	47.9	0	17.6	20.8	0.9
									0 ± 0	47.4 ± 1.6	0 ± 0	19.3 ± 1.6	20.0 ± 0.4	0.9 ± 0
**16**	none	0.02337	0	0	0	2.3276	0	0.0226	0	0	0	90.7	0	0.9
**17**	none	0.02337	0	0	0	2.3863	0	0.0324	0	0	0	92.9	0	1.3
**18**	none	0.02337	0	0	0	2.3259	0	0.0196	0	0	0	90.6	0	0.8
									0 ± 0	0 ± 0	0 ± 0	91.4 ± 0.8	0 ± 0	1.0 ± 0.2

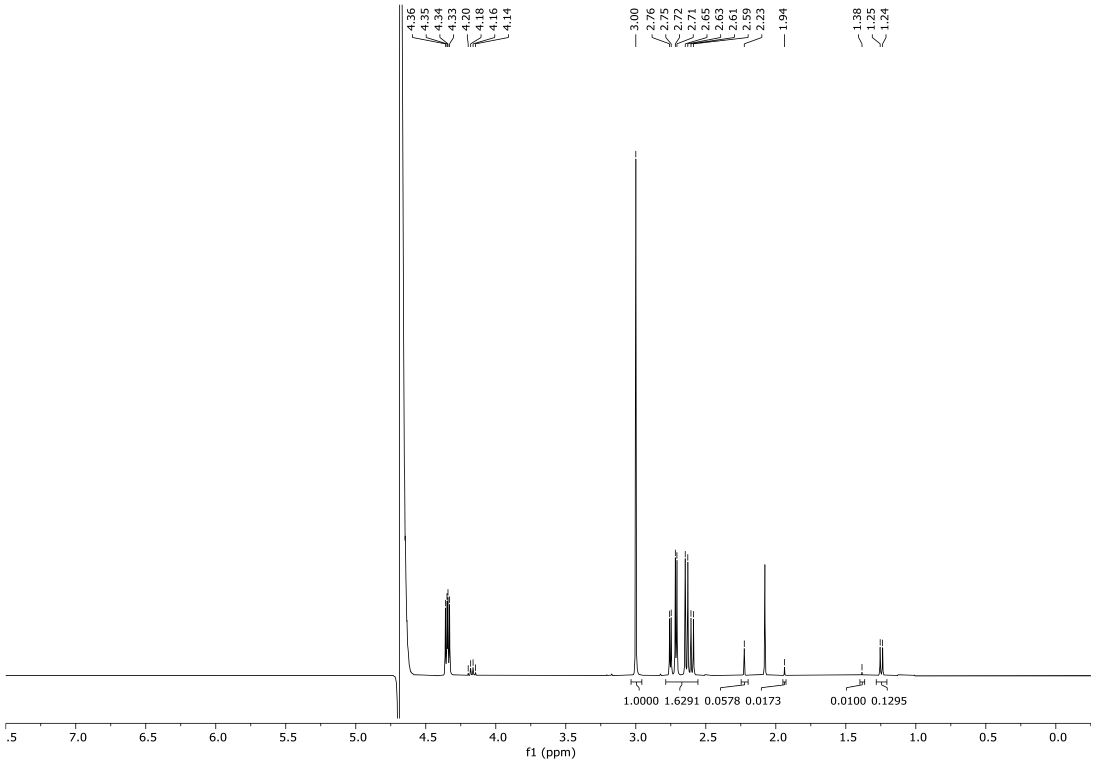

Figure S39. Representative ^{1}H qNMR using Rh/Al_2O_3 as catalyst at 5 bar H_2 (Table S4 entry 1−3).

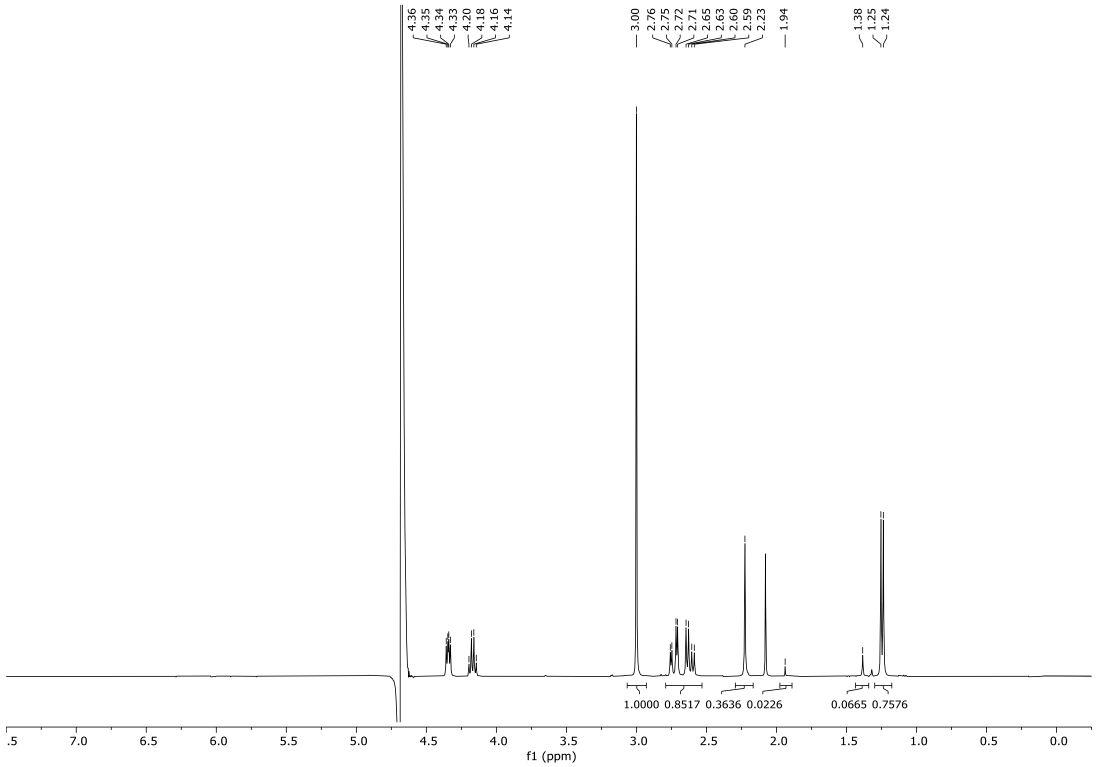

Figure S40. Representative ^{1}H qNMR using Ni/SiO_2-Al_2O_3 as catalyst at 5 bar H_2 (Table S4 entry 4−6).

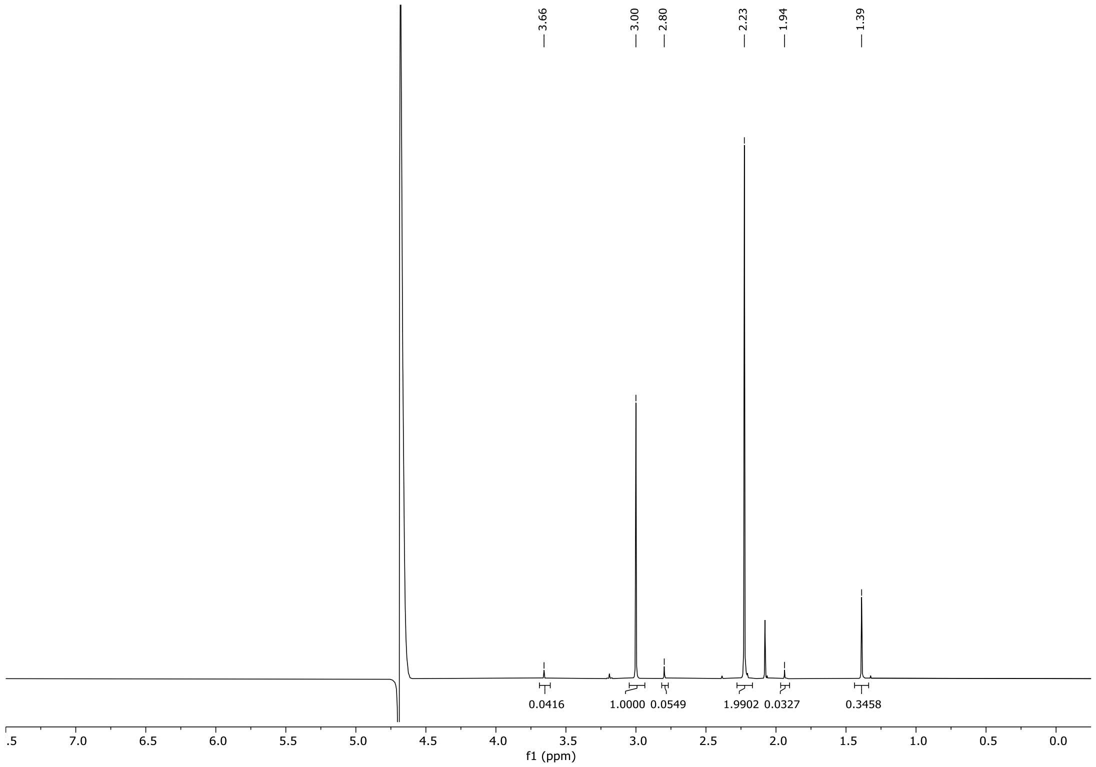

Figure S41. Representative ^{1}H qNMR without catalyst at 5 bar H_2 (Table S4 entry 7−9).

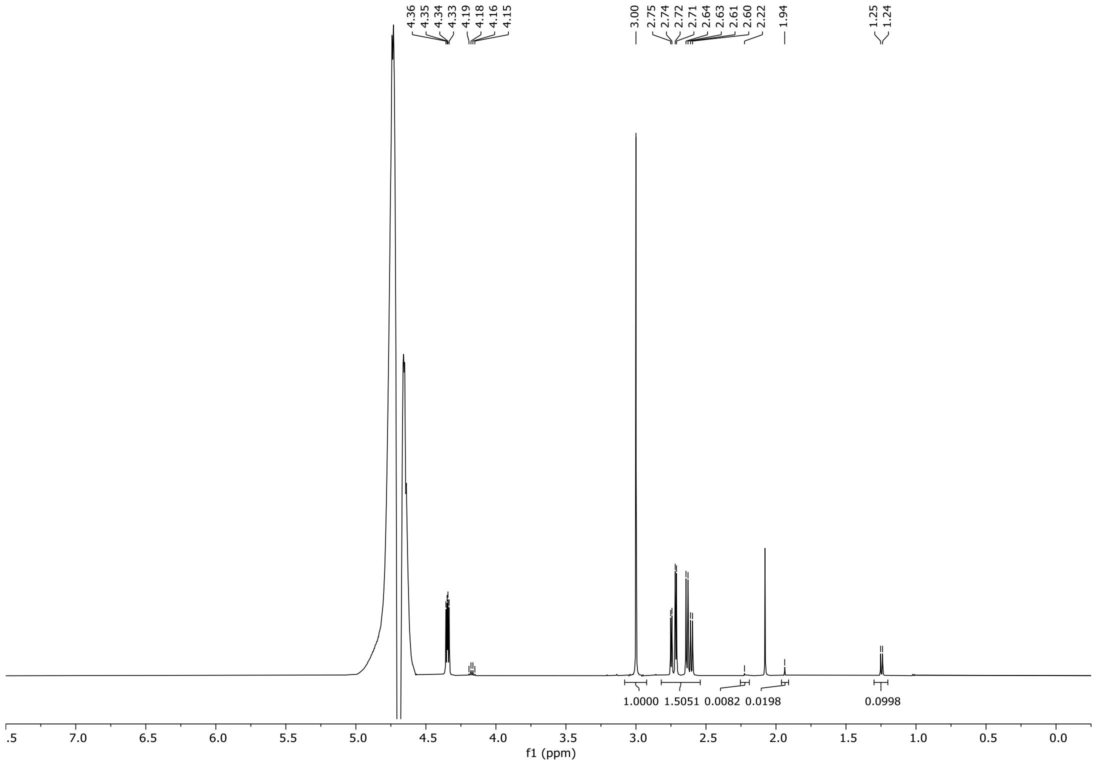

Figure S42. Representative ^{1}H qNMR using Rh/Al_2O_3 as catalyst at 10 bar H_2 (Table S4 entry 10−12).

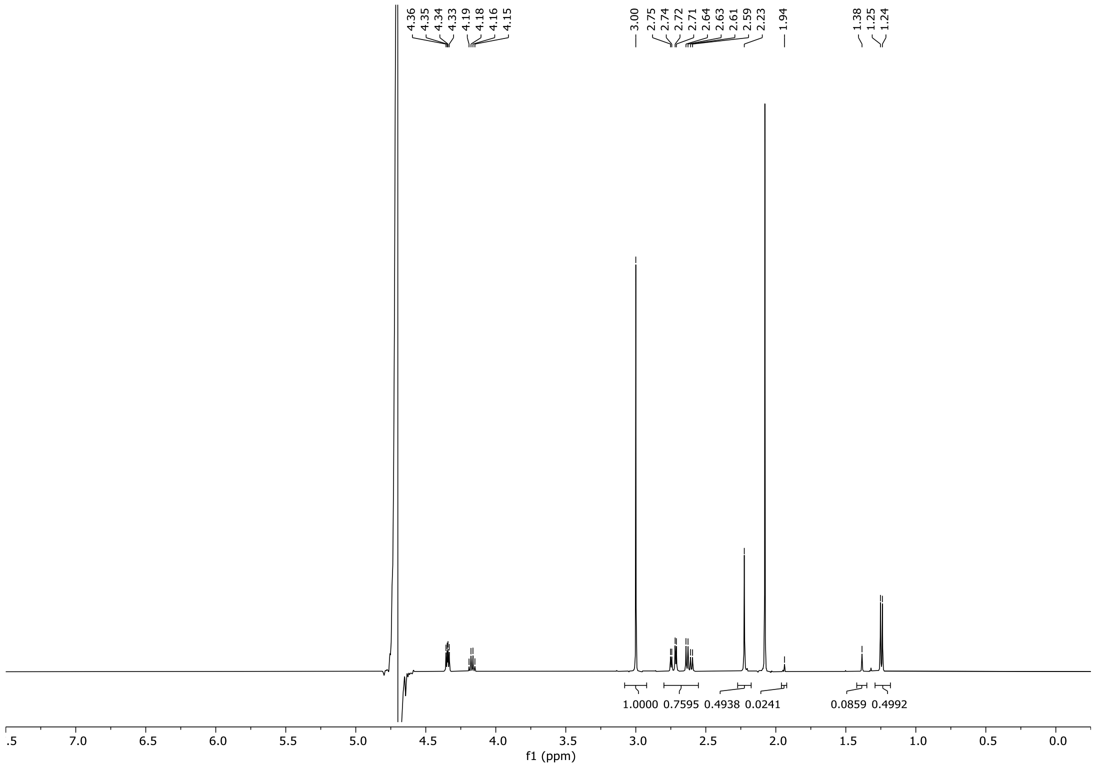

Figure S43. Representative ^{1}H qNMR using Ni/SiO_2-Al_2O_3 as catalyst at 10 bar H_2 (Table S4 entry 13−15).

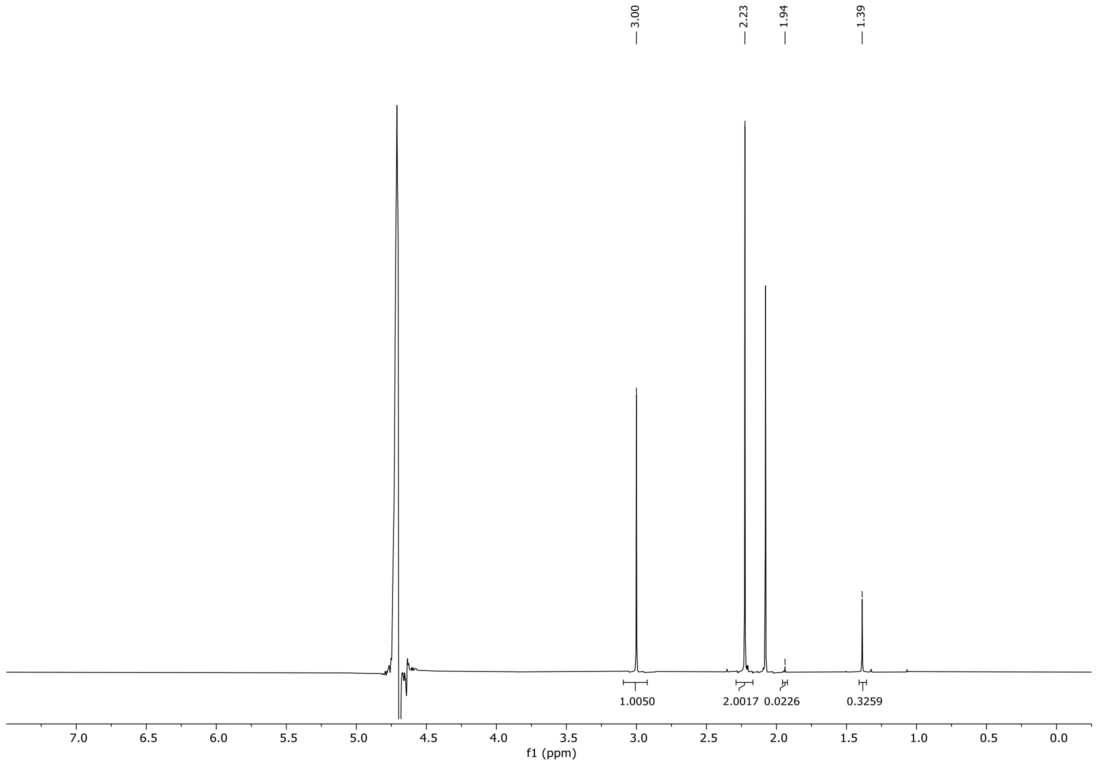

Figure S44. Representative ^{1}H qNMR without catalyst at 10 bar H_2 (Table S4 entry 16−18).

2.4.3 Variation of temperature

Table S5. Reaction conditions: oxaloacetic acid (30 mM), Rh/Al_2O_3 (1 mol%) or Ni/SiO_2-Al_2O_3 (10 mol%), H_2 (1 atm), pH 7−8, 5, 40 **or** 60 °C, 18 h.

			integrals relative to DMS (6H) set to 1.0000 at 3.0000 ppm						yield [%]					
#	catalyst loading (equiv., mol%, mg)	conc. DMS [M]	oxalo-acetate (2H)	malate (2H)	succi-nate (4H)	pyruvate (3H)	lactate (3H)	acetate (3H)	oxalo-acetate	malate	succi-nate	pyruvate	lactate	acetate
5 °C														
1	Rh/Al_2O_3 (0.01 equiv., 1 mol%, 0.62 mg)	0.02221	0	1.3077	0	0.0163	0.0880	0.0269	0	72.6	0	0.6	3.3	1.0
2	Rh/Al_2O_3 (0.01 equiv., 1 mol%, 0.62 mg)	0.02221	0	1.6987	0	0.0080	0.1054	0.0206	0	94.3	0	0.3	3.9	0.8
3	Rh/Al_2O_3 (0.01 equiv., 1 mol%, 0.62 mg)	0.02221	0	1.5423	0	0	0.0874	0.0205	0	85.6	0	0	3.2	0.8
									0 ± 0	84.2 ± 6.3	0 ± 0	0.3 ± 0.2	3.5 ± 0.2	0.8 ± 0.1
4	Ni/SiO_2-Al_2O_3 (0.1 equiv., 10 mol%, 0.26 mg)	0.02221	0.0606	0.2579	0	1.6466	0.0343	0.0229	6.7	14.3	0	61.0	1.3	0.8
5	Ni/SiO_2-Al_2O_3 (0.1 equiv., 10 mol%, 0.26 mg)	0.02221	0.0851	0.2783	0	1.6174	0.0289	0.0220	9.5	15.5	0	59.9	1.1	0.8
6	Ni/SiO_2-Al_2O_3 (0.1 equiv., 10 mol%, 0.26 mg)	0.02221	0.0589	0.3225	0	1.6526	0.0259	0.0153	6.5	17.9	0	61.2	1.0	0.6
									7.6 ± 0.9	15.9 ± 1.1	0 ± 0	60.7 ± 0.4	1.1 ± 0.1	0.7 ± 0.1
7	none	0.02221	0.2226	0	0	1.6054	0	0.0151	24.7	0	0	59.4	0	0.6
8	none	0.02221	0.2547	0	0	1.8708	0	0.0199	28.3	0	0	69.3	0	0.7
9	none	0.02221	0.2435	0	0	1.8849	0	0.0169	27.0	0	0	69.8	0	0.6
									26.7 ± 1.0	0 ± 0	0 ± 0	66.2 ± 3.4	0 ± 0	0.6 ± 0.1
40 °C														
10	Rh/Al_2O_3 (0.01 equiv., 1 mol%, 0.62 mg)	0.02337	0	0.6849	0.0010	0.7041	0.6081	0.0273	0	40.0	0	27.4	23.7	1.1
11	Rh/Al_2O_3 (0.01 equiv., 1 mol%, 0.62 mg)	0.02337	0	0.7175	0.0015	0.8459	0.4535	0.0273	0	41.9	0	32.9	17.7	1.1
12	Rh/Al_2O_3 (0.01 equiv., 1 mol%, 0.62 mg)	0.02337	0	0.6625	0	0.9326	0.4180	0.0264	0	38.7	0	36.3	16.3	1.0
									0 ± 0	40.2 ± 0.9	0 ± 0	32.2 ± 2.6	19.2 ± 2.3	1.1 ± 0
13	Ni/SiO_2-Al_2O_3 (0.1 equiv., 10 mol%, 0.26 mg)	0.02337	0	0.2560	0	1.0553	0.3967	0.1070	0	15.0	0	41.1	15.5	4.2
14	Ni/SiO_2-Al_2O_3 (0.1 equiv., 10 mol%, 0.26 mg)	0.02337	0	0.3032	0.0007	0.8461	0.6109	0.1847	0	17.7	0	33.0	23.8	7.2
15	Ni/SiO_2-Al_2O_3 (0.1 equiv., 10 mol%, 0.26 mg)	0.02337	0	0.2551	0	1.2197	0.3228	0.0622	0	14.9	0	40.7	12.6	2.4
									0 ± 0	15.9 ± 0.9	0 ± 0	40.5 ± 4.2	17.3 ± 3.4	4.6 ± 1.4

			integrals relative to DMS (6H) set to 1.0000 at 3.0000 ppm						yield [%]					
**#**	**catalyst loading (equiv., mol%, mg)**	**conc. DMS [M]**	**oxalo-acetate (2H)**	**malate (2H)**	**succi-nate (4H)**	**pyruvate (3H)**	**lactate (3H)**	**acetate (3H)**	**oxalo-acetate**	**malate**	**succi-nate**	**pyruvate**	**lactate**	**acetate**
**16**	none	0.02337	0	0	0	2.5208	0	0.0255	0	0	0	98.2	0	1.0
**17**	none	0.02337	0	0	0	2.5308	0	0.0276	0	0	0	98.6	0	1.1
**18**	none	0.02337	0	0	0	2.5454	0	0.0211	0	0	0	99.1	0	0.8
									0 ± 0	0 ± 0	0 ± 0	98.6 ± 0.3	0 ± 0	1.0 ± 0.1
**60 °C**														
**19**	Rh/Al_2O_3 (0.01 equiv., 1 mol%, 0.62 mg)	0.02337	0	0.2582	0	1.5519	0.4599	0.0541	0	15.1	0	60.4	17.9	2.1
**20**	Rh/Al_2O_3 (0.01 equiv., 1 mol%, 0.62 mg)	0.02337	0	0.2699	0	1.5261	0.5108	0.0564	0	15.8	0	59.4	19.9	2.2
**21**	Rh/Al_2O_3 (0.01 equiv., 1 mol%, 0.62 mg)	0.02337	0	0.2841	0	1.6097	0.3532	0.0673	0	16.6	0	62.7	13.8	2.6
									0 ± 0	15.8 ± 0.4	0 ± 0	60.9 ± 1.0	17.2 ± 1.8	2.3 ± 0.2
**22**	Ni/SiO_2-Al_2O_3 (0.1 equiv., 10 mol%, 0.26 mg)	0.02337	0	0.1643	0	0.1213	1.5684	0.0484	0	9.6	0	4.7	61.1	1.9
**23**	Ni/SiO_2-Al_2O_3 (0.1 equiv., 10 mol%, 0.26 mg)	0.02337	0	0.1716	0	0.3433	1.7585	0.0730	0	10.0	0	13.4	68.5	2.8
**24**	Ni/SiO_2-Al_2O_3 (0.1 equiv., 10 mol%, 0.26 mg)	0.02337	0	0.2260	0	0.0520	1.9981	0.0600	0	13.2	0	2.0	77.8	2.3
									0 ± 0	10.9 ± 1.1	0 ± 0	6.7 ± 3.4	69.1 ± 4.8	2.4 ± 0.3
**25**	none	0.02337	0	0	0	2.2696	0	0.0359	0	0	0	88.4	0	1.4
**26**	none	0.02337	0	0	0	2.1756	0	0.0225	0	0	0	84.7	0	0.9
**27**	none	0.02337	0	0	0	2.2980	0	0.0241	0	0	0	89.5	0	0.9
									0 ± 0	0 ± 0	0 ± 0	87.4 ± 1.4	0 ± 0	1.1 ± 0.2

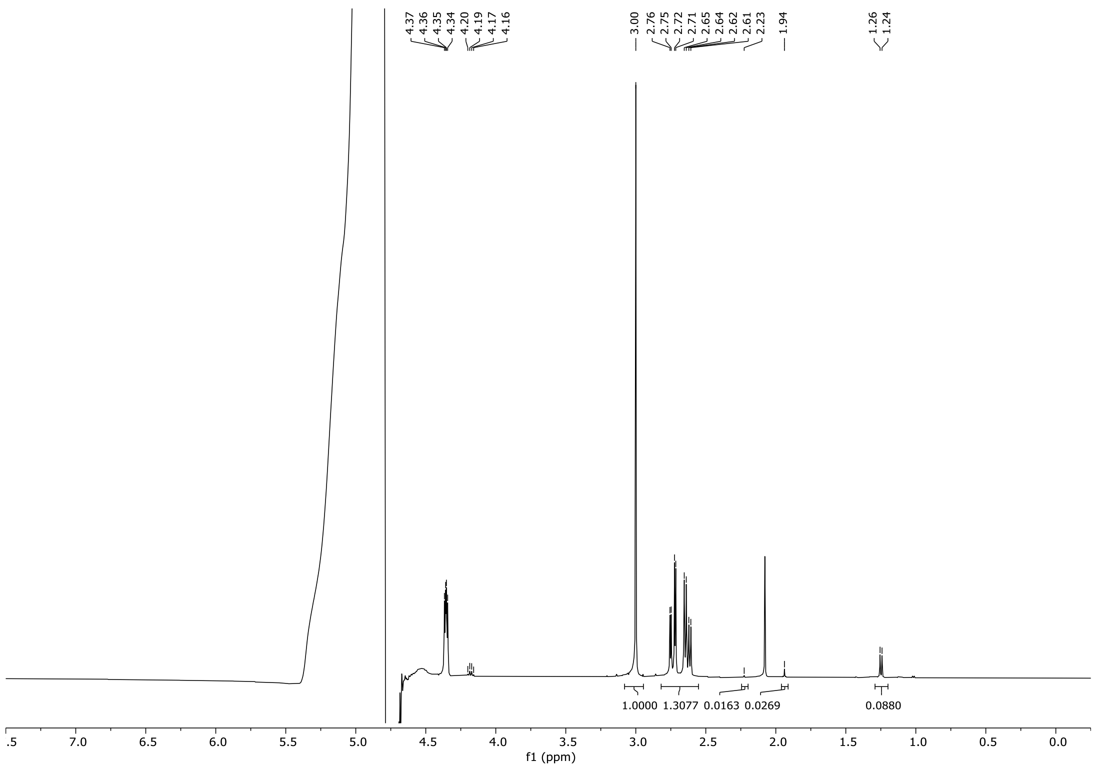

Figure S45. Representative ^{1}H qNMR using Rh/Al_2O_3 as catalyst at 5 °C (Table S5 entry 1−3).

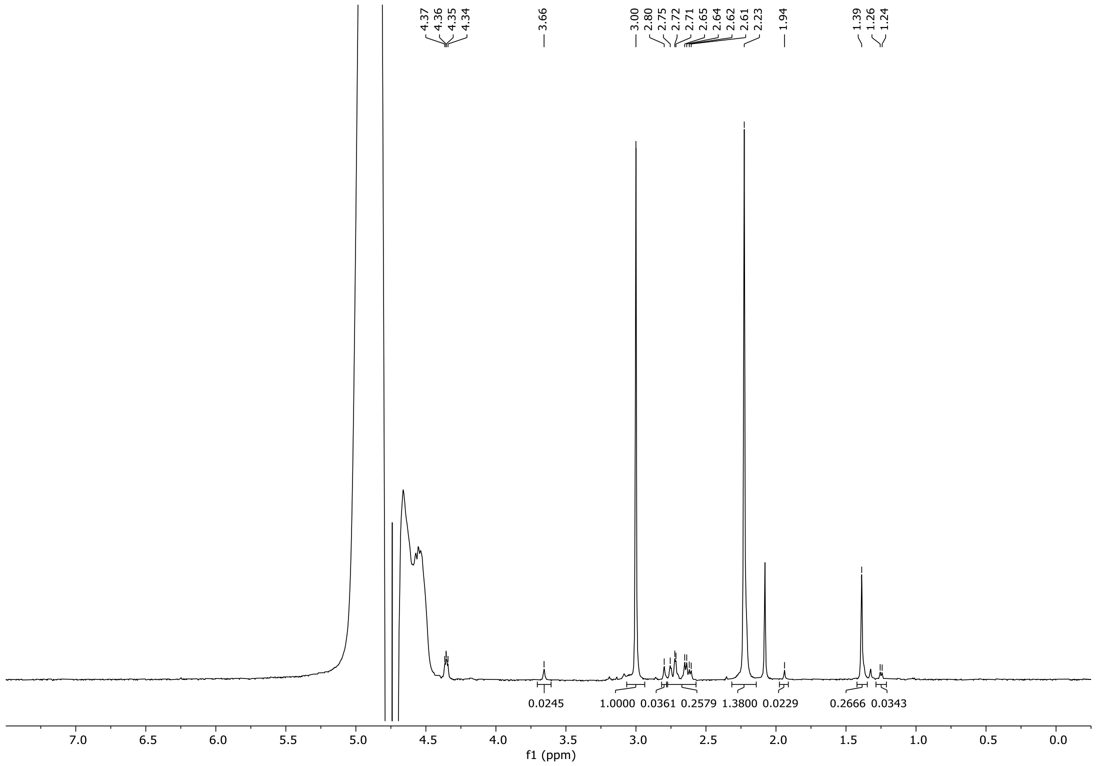

Figure S46. Representative ^{1}H qNMR using Ni/SiO_2-Al_2O_3 as catalyst at 5 °C (Table S5 entry 4−6).

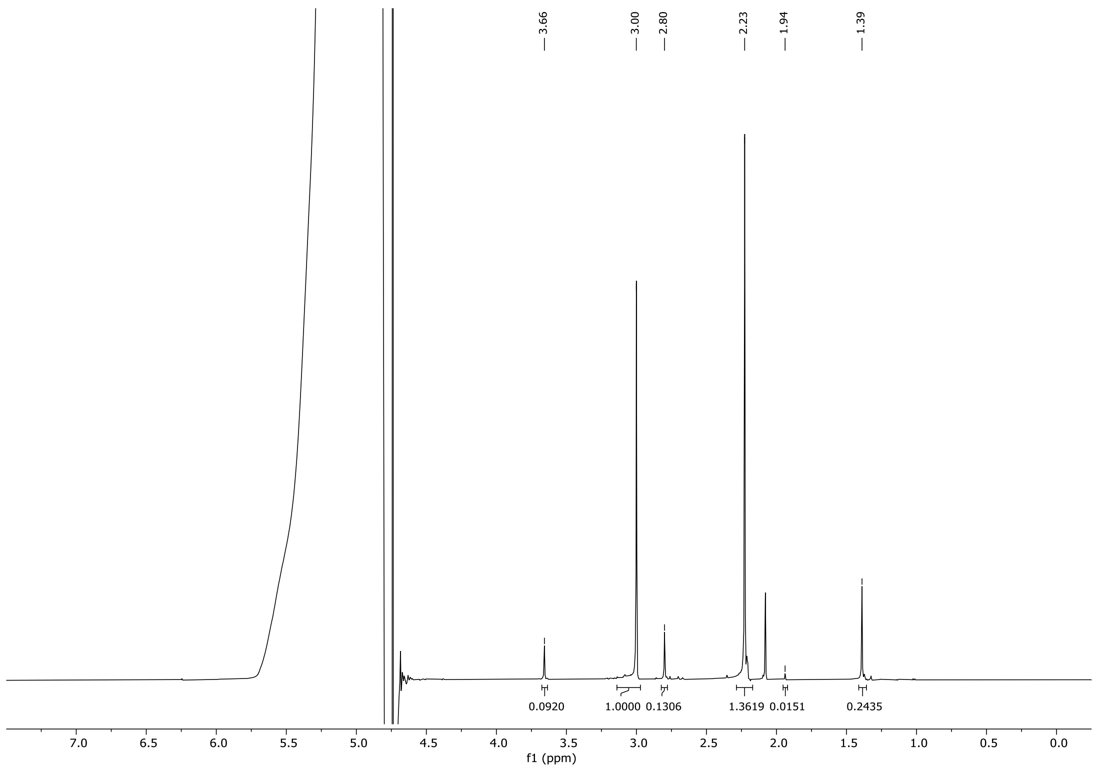

Figure S47. Representative ^{1}H qNMR without catalyst at 5 °C (Table S5 entry 7−9).

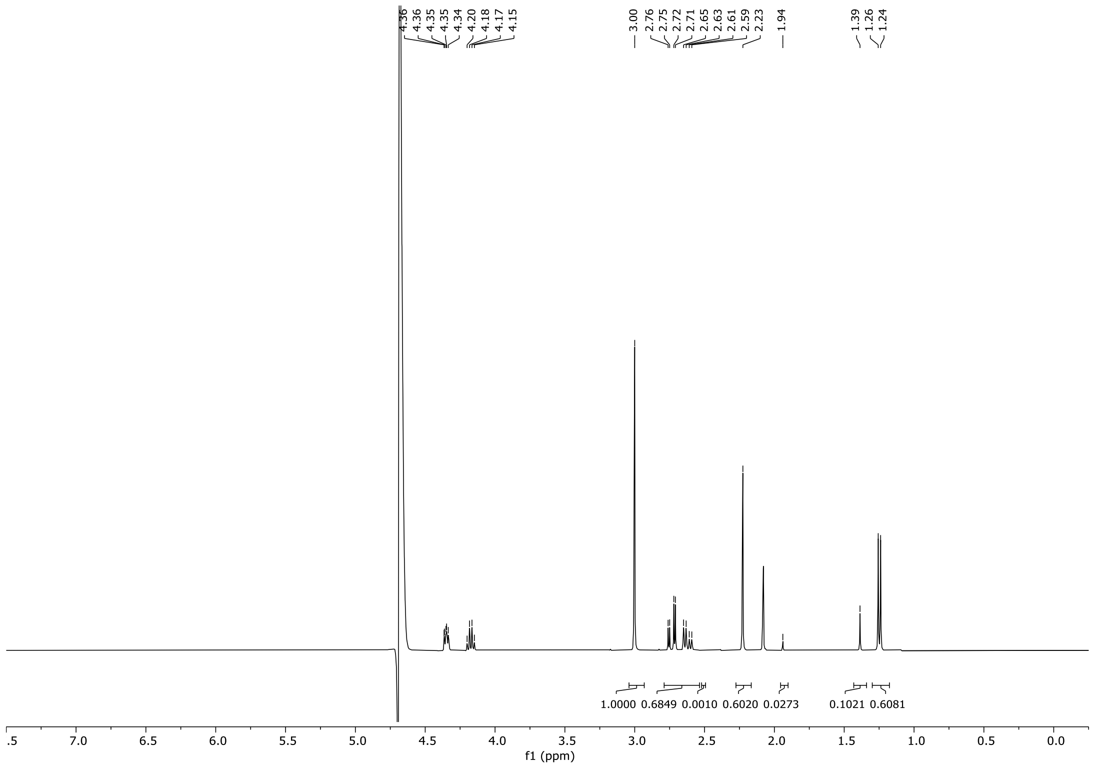

Figure S48. Representative ^{1}H qNMR using Rh/Al_2O_3 as catalyst at 40 °C (Table S5 entry 10−12).

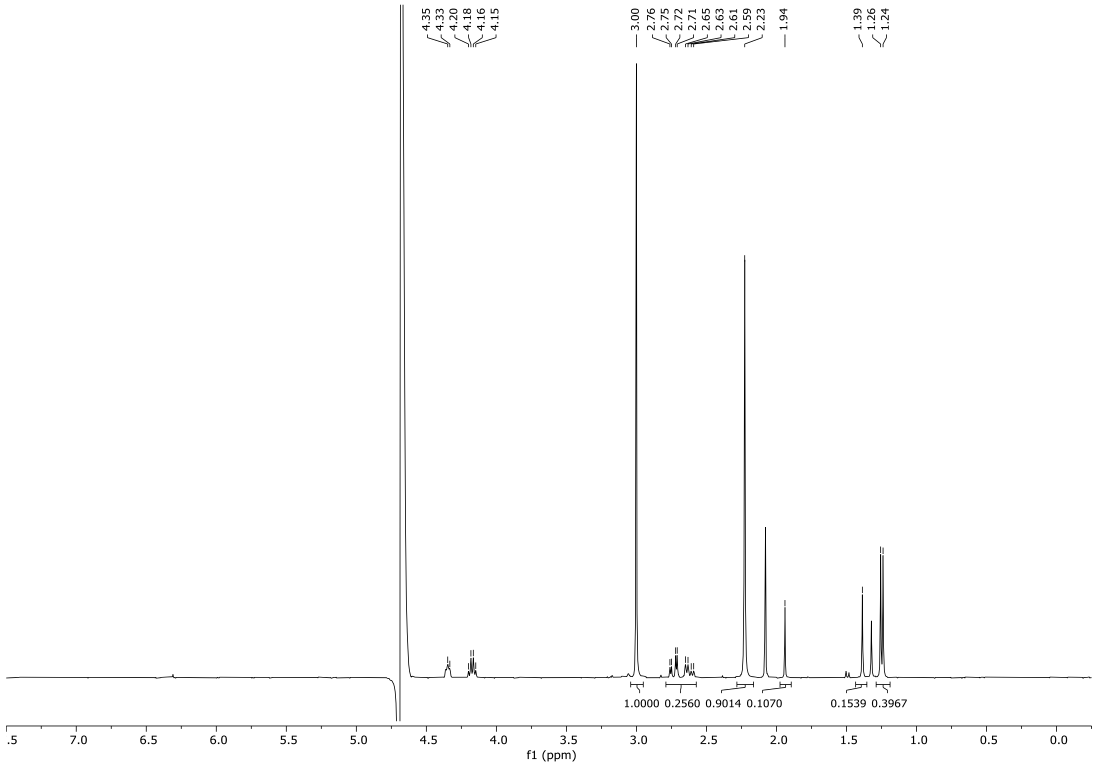

Figure S49. Representative ^{1}H qNMR using Ni/SiO_2-Al_2O_3 as catalyst at 40 °C (Table S5 entry 13−15).

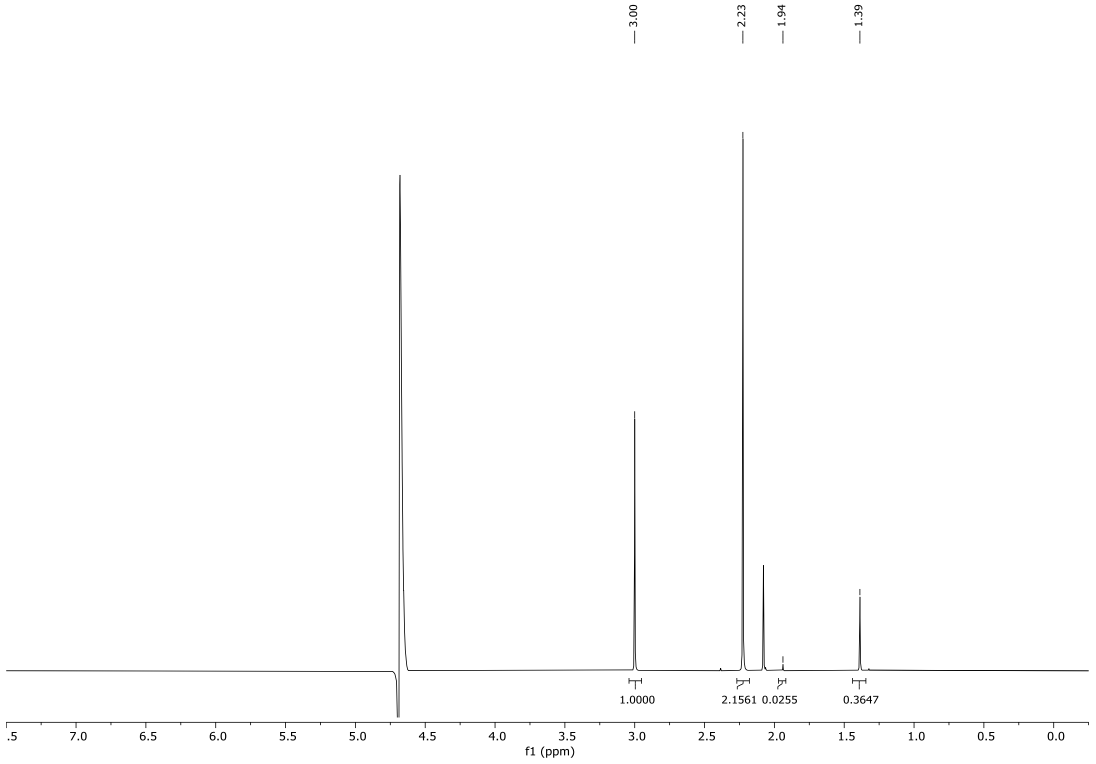

Figure S50. Representative ^{1}H qNMR without catalyst at 40 °C (Table S5 entry 16−18).

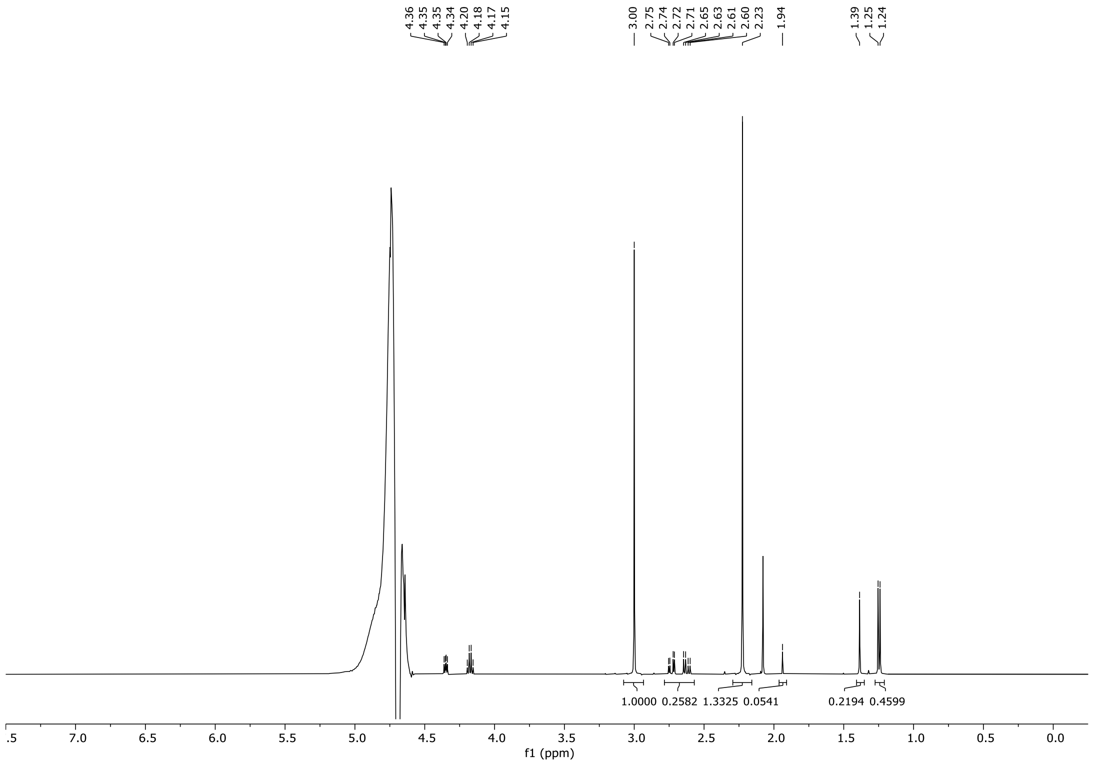

Figure S51. Representative ^{1}H qNMR using Rh/Al_2O_3 as catalyst at 60 °C (Table S5 entry 19−21).

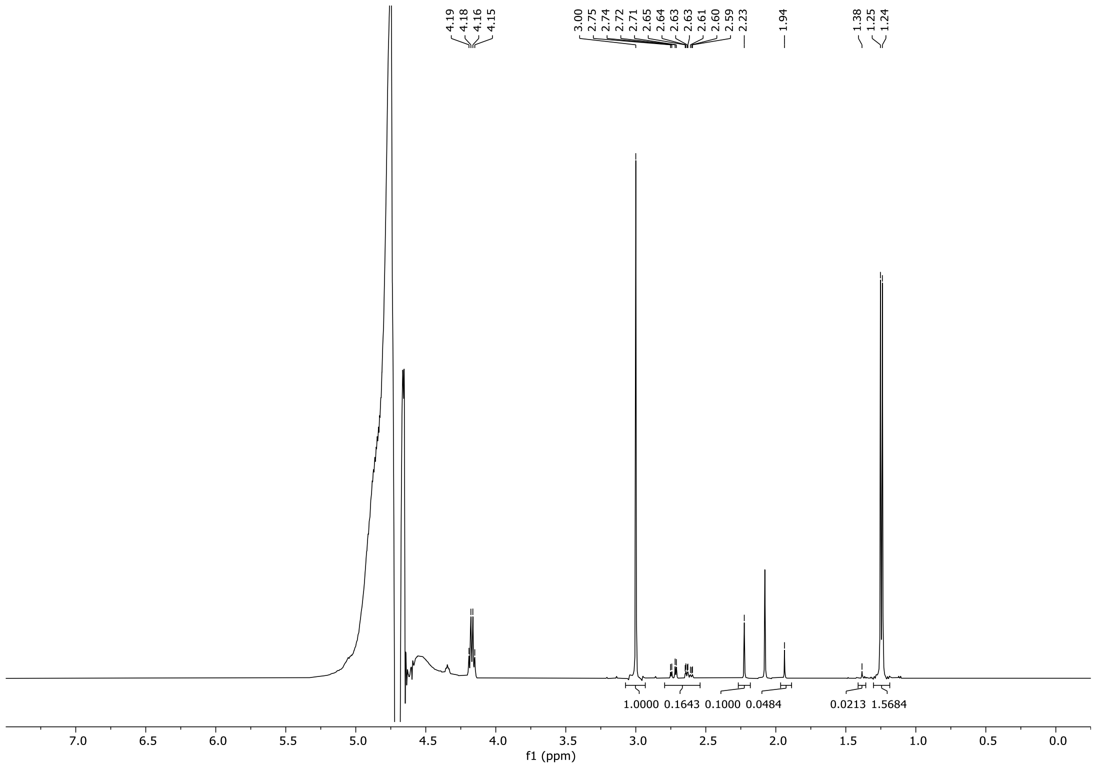

Figure S52. Representative ^{1}H qNMR using Ni/SiO_2-Al_2O_3 as catalyst at 60 °C (Table S5 entry 22−24).

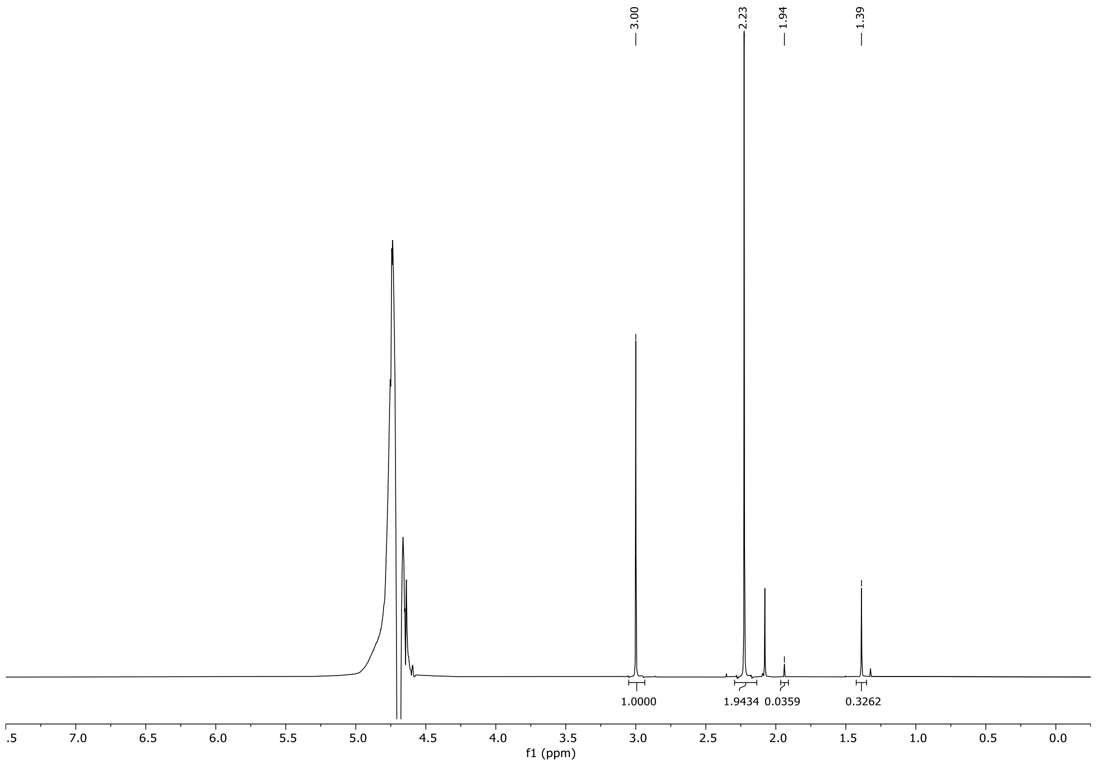

Figure S 53. Representative ^{1}H qNMR without catalyst at 60 °C (Table S5 entry 25−27).

2.4.4 Variation of pH

Table S6. Reaction conditions: oxaloacetic acid (30 mM), Rh/Al_2O_3 (1 mol%) or Ni/SiO_2-Al_2O_3 (10 mol%), H_2 (1 atm), pH 2 **or** 11, 22 °C, 18 h.

			integrals relative to DMS (6H) set to 1.0000 at 3.0000 ppm						yield [%]					
**#**	**catalyst loading (equiv., mol%, mg)**	**conc. DMS [M]**	**oxalo-acetate (2H)**	**malate (2H)**	**succi-nate (4H)**	**pyruvate (3H)**	**lactate (3H)**	**acetate (3H)**	**oxalo-acetate**	**malate**	**succi-nate**	**pyruvate**	**lactate**	**acetate**
						**pH 2 (unadjusted)**								
**1**	Rh/Al_2O_3 (0.01 equiv., 1 mol%, 0.62 mg)	0.02577	0	1.1725	0.1525	0.0082	0.4121	0.0212	0	75.5	4.9	0.4	17.7	0.9
**2**	Rh/Al_2O_3 (0.01 equiv., 1 mol%, 0.62 mg)	0.02577	0	1.3295	0.1699	0.0040	0.2134	0.0184	0	85.7	5.5	0.2	9.2	0.8
**3**	Rh/Al_2O_3 (0.01 equiv., 1 mol%, 0.62 mg)	0.02577	0	1.2041	0.1607	0.0082	0.4188	0.0221	0	77.6	5.2	0.4	18.0	0.9
									0 ± 0	79.6 ± 3.1	5.2 ± 0.2	0.3 ± 0.1	15.0 ± 2.9	0.9 ± 0
**4**	Ni/SiO_2-Al_2O_3 (0.1 equiv., 10 mol%, 0.26 mg)	0.02577	0	0.0494[a]	0	1.7572	0	0.0178	0	6.4	0	75.5	0	0.8
**5**	Ni/SiO_2-Al_2O_3 (0.1 equiv., 10 mol%, 0.26 mg)	0.02577	0	0.0545[a]	0	1.8085	0	0.0166	0	7.0	0	77.7	0	0.7
**6**	Ni/SiO_2-Al_2O_3 (0.1 equiv., 10 mol%, 0.26 mg)	0.02577	0	0.0455[a]	0	1.7353	0	0.0142	0	5.9	0	74.5	0	0.6
									0 ± 0	6.4 ± 0.3	0 ± 0	75.9 ± 0.9	0 ± 0	0.7 ± 0
						**pH 11**								
**7**	Rh/Al_2O_3 (0.01 equiv., 1 mol%, 0.62 mg)	0.02027	0	1.9107	0	0	0.1519	0.0226	0	96.8	0	0	5.1	0.8
**8**	Rh/Al_2O_3 (0.01 equiv., 1 mol%, 0.62 mg)	0.02027	0	1.8776	0	0	0.1514	0.0293	0	95.1	0	0	5.1	1.0
									0 ± 0	96.0 ± 0.8	0 ± 0	0 ± 0	5.1 ± 0	0.9 ± 0.1
**9**[b]	Ni/SiO_2-Al_2O_3 (0.1 equiv., 10 mol%, 0.26 mg)	0.02027	0	0.2640	0	0.9726	0.0907	0.0875	0	26.8	0	65.7	6.1	5.9
**10**	Ni/SiO_2-Al_2O_3 (0.1 equiv., 10 mol%, 0.26 mg)	0.02027	0	0.4147	0	1.7557	0.1529	0.0336	0	21.0	0	59.3	5.2	1.1
**11**	Ni/SiO_2-Al_2O_3 (0.1 equiv., 10 mol%, 0.26 mg)	0.02027	0	0.5369	0	1.4664	0.2073	0.0365	0	27.2	0	49.5	7.0	1.2
									0 ± 0	25.0 ± 2.0	0 ± 0	58.2 ± 4.7	6.1 ± 0.5	2.8 ± 1.6

[a] Quantification of malate by integration of one half of the qd from 2.60−2.66 ppm corresponding to one proton [b] NMR preparation: 50 µL DMS + 100 µL centrifuged reaction mixture, 600 µL phosphate buffer (1 M, pH 3)

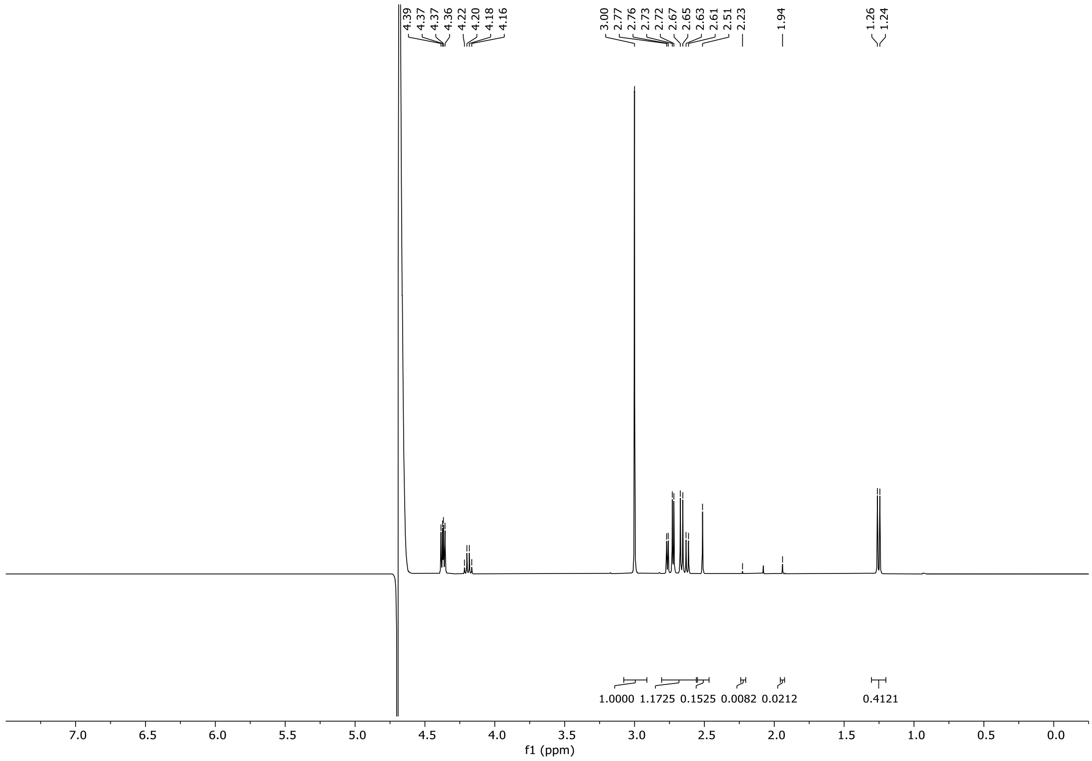

Figure S54. Representative ^{1}H qNMR using Rh/Al_2O_3 as catalyst at pH 2 (Table S6 entry 1−3).

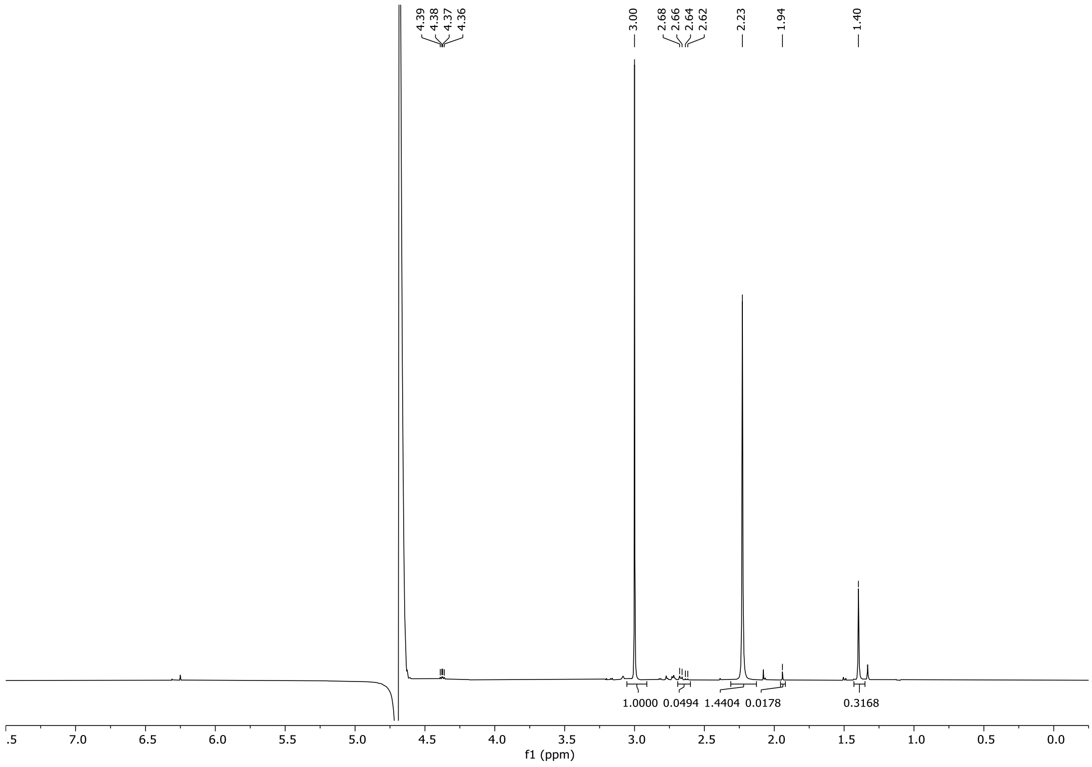

Figure S55. Representative ^{1}H qNMR using Ni/SiO_2-Al_2O_3 as catalyst at pH 2 (Table S6 entry 4−6).

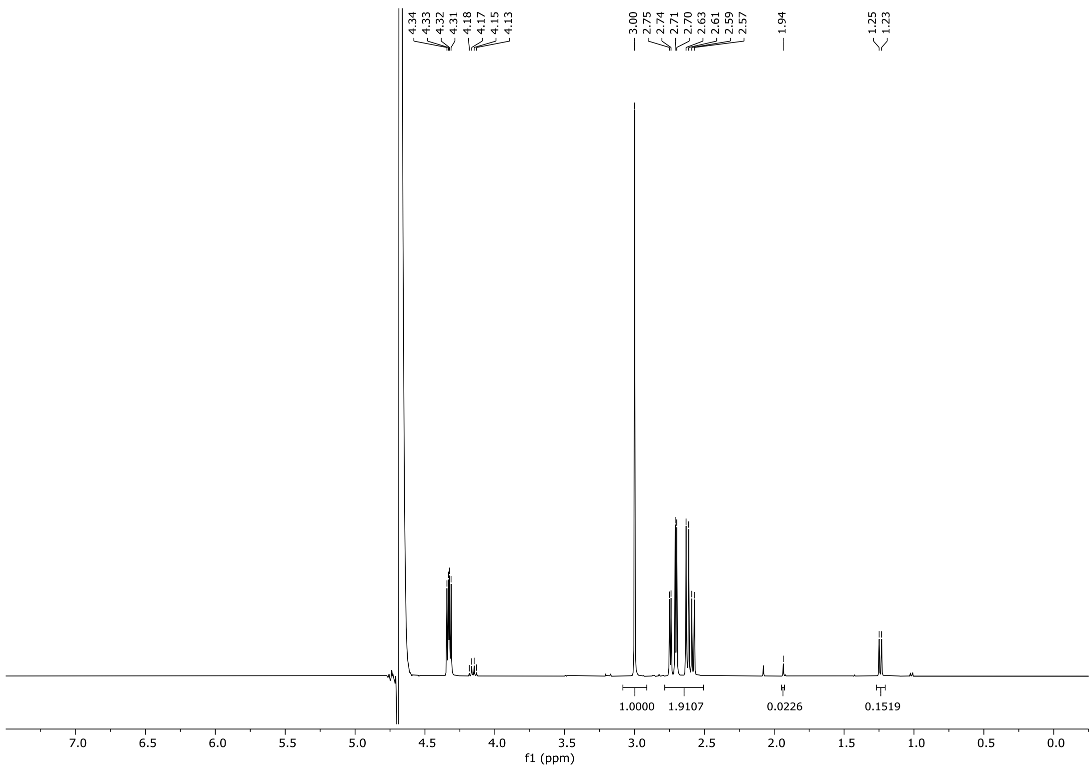

Figure S56. Representative ^{1}H qNMR using Rh/Al_2O_3 as catalyst at pH 11 (Table S6 entry 7+8).

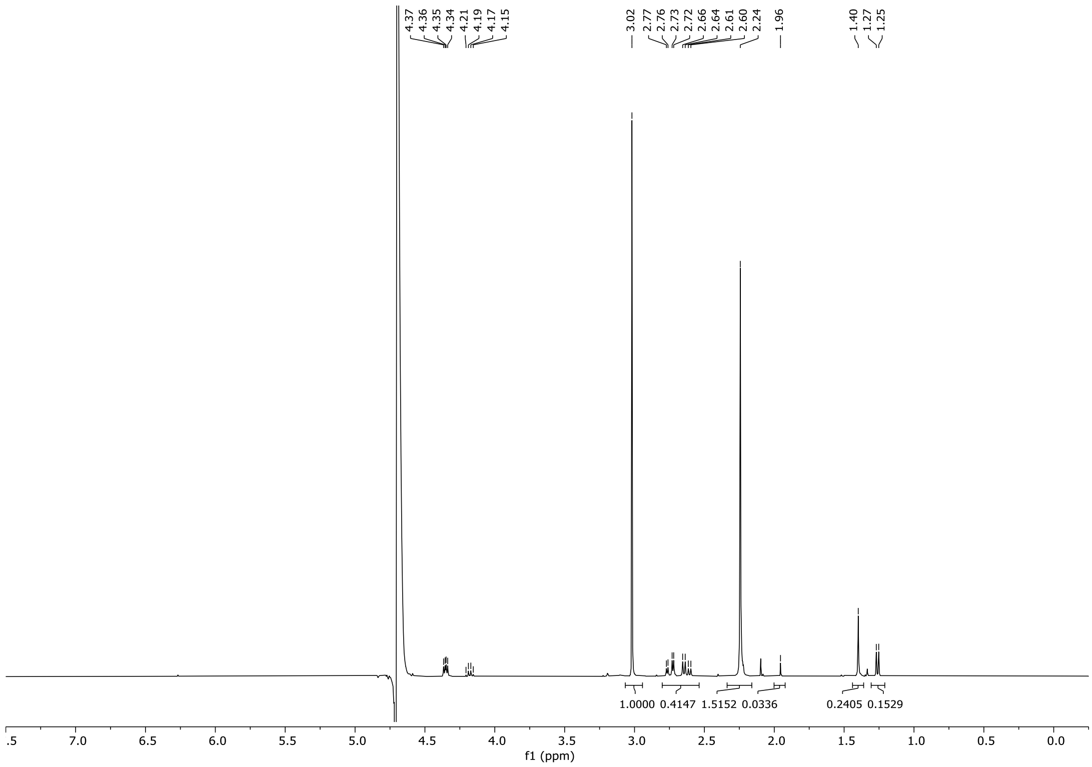

Figure S57. Representative ^{1}H qNMR using Ni/SiO_2-Al_2O_3 as catalyst at pH 11 (Table S6 entry 9−11).

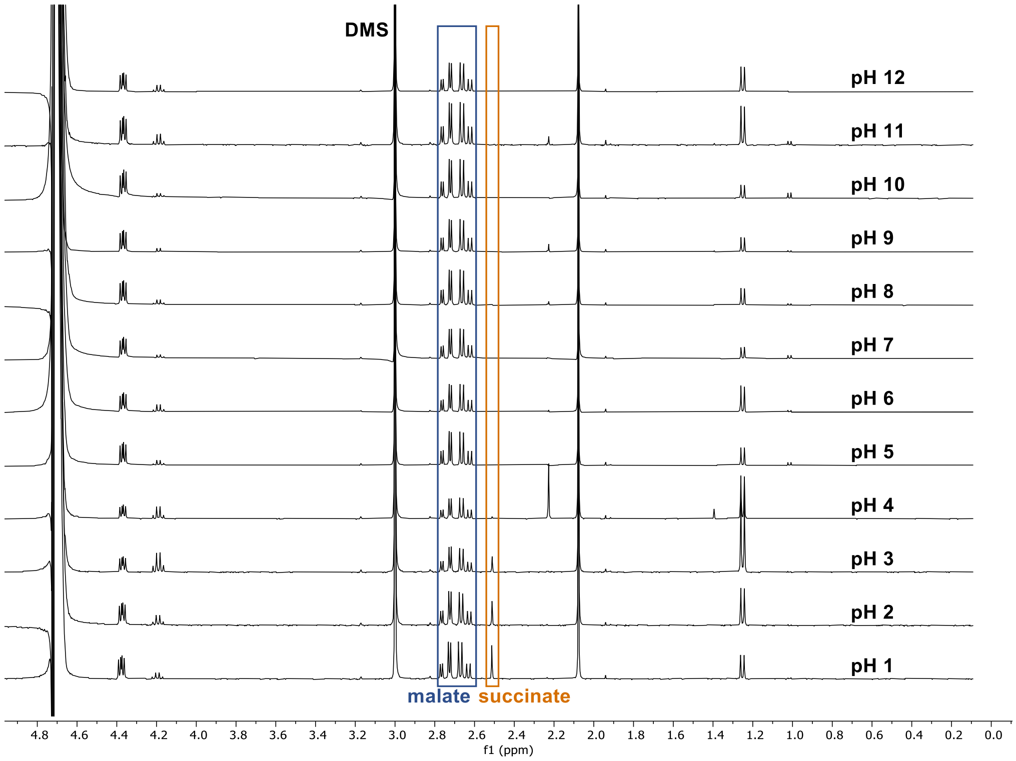

Figure S58. pH screen for Rh/Al_2O_3 (1 mol%).

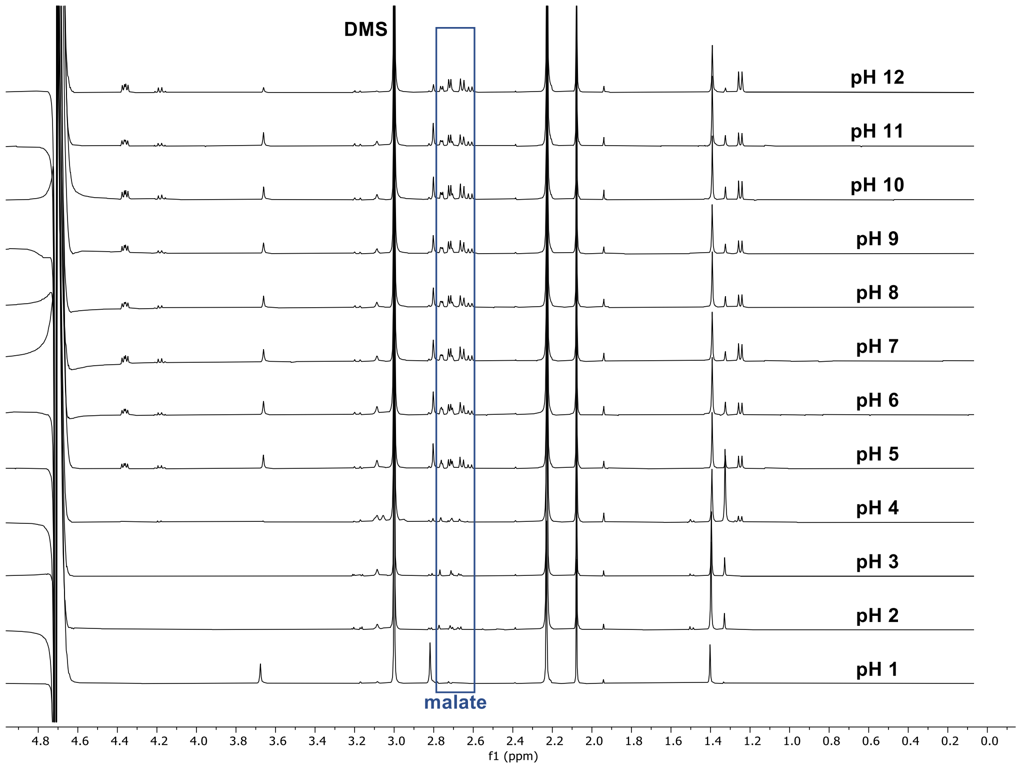

Figure S59. pH screen for Ni/SiO_2-Al_2O_3 (10 mol%).

2.4.5 Variation of catalyst loading

Rh/Al_2O_3 catalyst. A disposable glass vial with septum and PTFE stir bar (see section 1.2 for more details) was charged with Rh/Al_2O_3 (0.001 equiv., 0.1 mol%, 0.06 mg). Then, oxaloacetic acid (0.03 mmol, 3.96 mg, 1 equiv.) was added from an aqueous stock solution (0.06 M in water and adjusted to pH 7−8 with NaOH) and water was added to a total volume of 1 mL solvent. After purging the vial for 3 min with H_2, the vial was equipped with an H_2 balloon and was stirred at 22 °C for 18 h. Experiments were done in duplicates. qNMRs were acquired according to section 1.4.2. Mean values of yields and standard errors were calculated for each set of reactions.

Ni/SiO_2-Al_2O_3 catalyst. A flask vial with septum and PTFE stir bar was charged with Ni/SiO_2-Al_2O_3, 0.1 equiv., 1 mol%, 0.26 mg). Then, oxaloacetic acid (0.3 mmol, 39.6 mg, 1 equiv.) was added from an aqueous stock solution (0.06 M in water; adjusted to pH 7−8 with NaOH) and water was added to a total volume of 10 mL solvent. After purging the flask for 3 min with H_2, the flask was equipped with an H_2 balloon and was stirred at 22 °C for 18 h. Experiments were done in duplicates. qNMRs were take according to section 1.4.2. Mean values of yields and standard errors were calculated for each set of reactions.

Table S7. Reaction conditions: oxaloacetic acid (30 mM), Rh/Al_2O_3 (0.1 mol%) **or** Ni/SiO_2-Al_2O_3 (1 mol%), H_2 (1 atm), pH 7−8, 22 °C, 18 h.

			integrals relative to DMS (6H) set to 1.0000 at 3.0000 ppm						yield [%]					
#	catalyst loading (equiv., mol%, mg)	conc. DMS [M]	oxalo-acetate (2H)	malate (2H)	succi-nate (4H)	pyruvate (3H)	lactate (3H)	acetate (3H)	oxalo-acetate	malate	succi-nate	pyruvate	lactate	acetate
1	Rh/Al_2O_3 (0.001 equiv., 0.1 mol%, 0.06 mg)	0.02337	0	0.7702	0	1.0619	0.2201	0.0237	0	45.0	0	41.4	8.6	0.9
2	Rh/Al_2O_3 (0.001 equiv., 0.1 mol%, 0.06 mg)	0.02337	0	0.7550	0	1.0302	0.2787	0.0429	0	44.1	0	40.1	10.9	1.7
									0 ± 0	44.6 ± 0.4	0 ± 0	40.7 ± 0.6	9.7 ± 1.1	1.3 ± 0.4
3	Ni/SiO_2-Al_2O_3 (0.01 equiv., 1 mol%, 0.26 mg)	0.02337	0.0345	0.0069[a]	0	1.8947	0.0056	0.0175	2.0	0.8	0	73.8	0.2	0.7
4	Ni/SiO_2-Al_2O_3 (0.01 equiv., 1 mol%, 0.26 mg)	0.02337	0.0291	0.0107[a]	0	1.8387	0.0125	0.0217	1.7	1.3	0	71.6	0.5	0.8
									1.9 ± 0.2	1.0 ± 0.2	0 ± 0	72.7 ± 1.1	0.4 ± 0.1	0.8 ± 0.1

[a] quantification of malate by integration of one half of the qd from 2.60−2.66 ppm corresponding to one proton

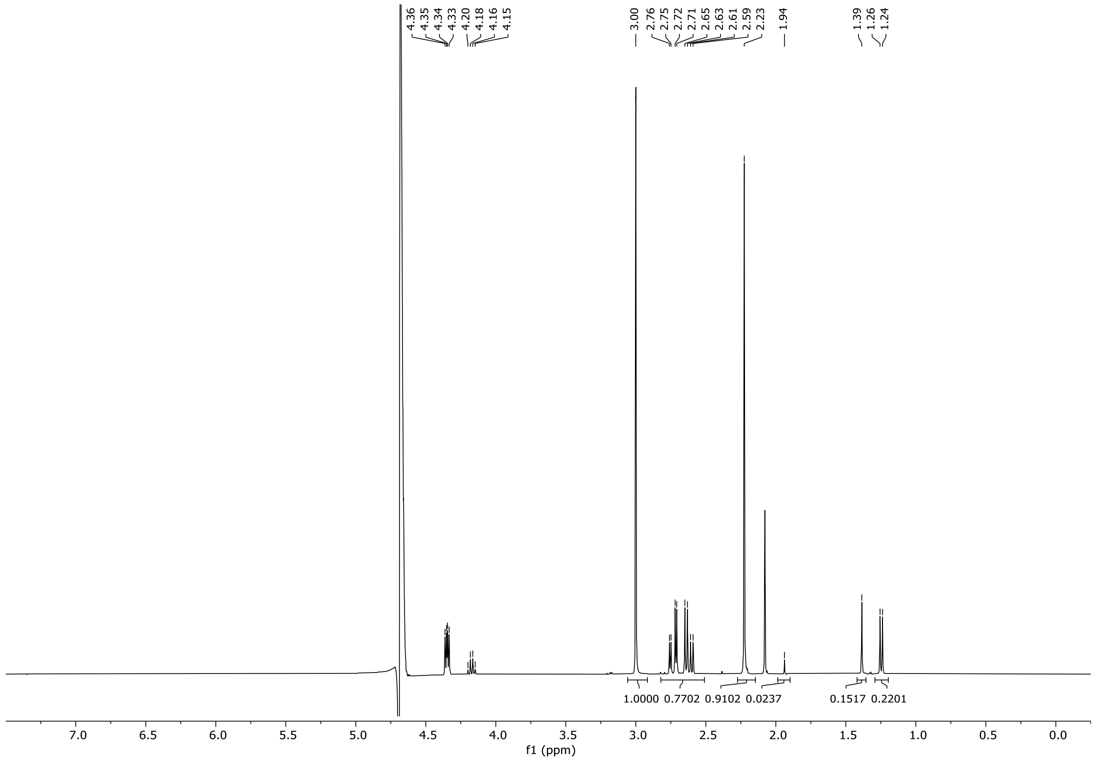

Figure S60. Representative ^{1}H qNMR using Rh/Al_2O_3 (0.1 mol%) as catalyst (Table S7 entry 1+2).

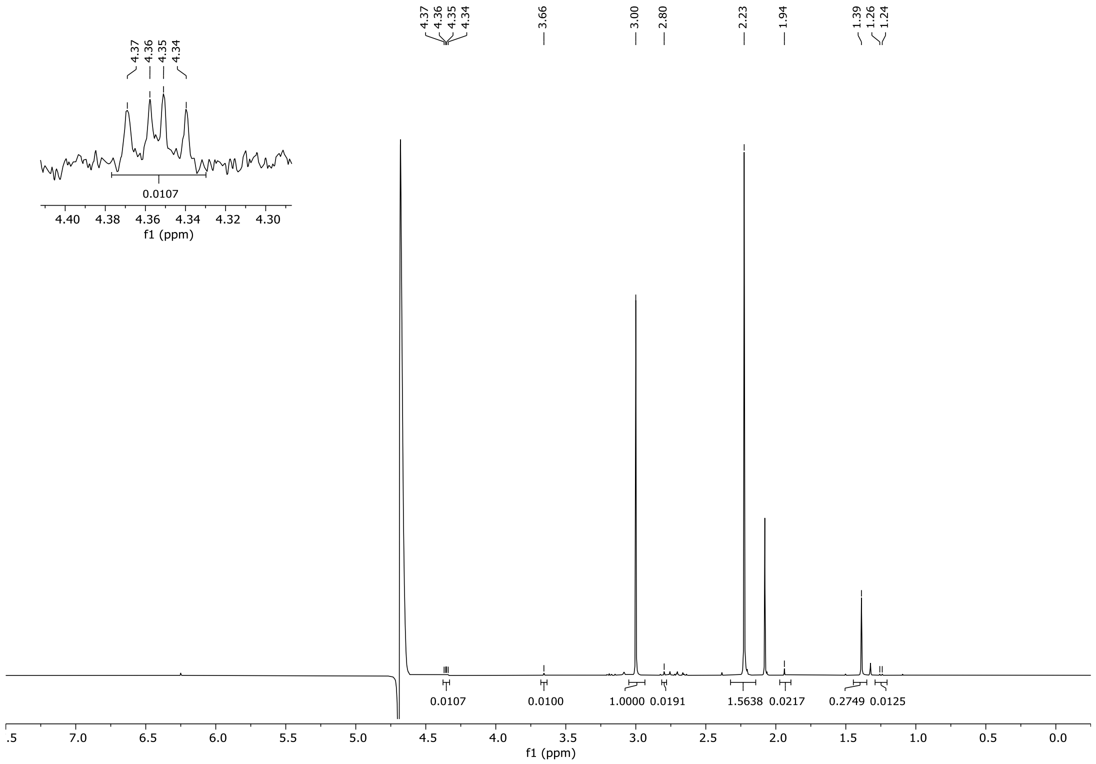

Figure S61. Representative ^{1}H qNMR using Ni/SiO_2-Al_2O_3 (1 mol%) as catalyst (Table S7 entry 3+4).

2.5 Malate under standard reaction conditions

A disposable glass vial with PTFE/silicone-lined septum (see section 1.2 for more details) was charged with the metal catalyst (Rh/Al_2O_3, 0.01 equiv., 1 mol%, 0.62 mg **or** Ni/SiO_2-Al_2O_3, 0.1 equiv., 10 mol%, 0.26 mg). Then, malic acid (0.03 mmol, 4.02 mg, 1 equiv.) was added from an aqueous stock solution (adjusted to pH 2−3 or 7−8; adjusted with NaOH or HCl) and water was added to a total volume of 1 mL solvent. After purging the vials for 3 min with H_2, the vials were equipped with an H_2 balloon and were stirred at 22 °C for 18 h. Experiments were done in triplicates if not otherwise noted. qNMRs were acquired according to section 1.4.2. Mean values of yields and standard errors were calculated for each set of reactions.

Table S8. Reaction conditions: malic acid (30 mM), Rh/Al_2O_3 (1 mol%) **or** Ni/SiO_2-Al_2O_3 (10 mol%), H_2 (1 atm), pH 2 **or** 7, 22 °C, 18 h.

			integrals relative to DMS (6H) set to 1.0000 at 3.0000 ppm						yield [%]					
#	catalyst loading (equiv., mol%, mg)	conc. DMS [M]	malate (2H)	fumarate (2H)	succi-nate (4H)	pyruvate (3H)	lactate (3H)	acetate (3H)	malate	fumarate	succi-nate	pyruvate	lactate	acetate
pH 2														
1	Rh/Al_2O_3 (0.01 equiv., 1 mol%, 0.62 mg)	0.02577	1.4788	0	0.0214	0	0	0	95.3	0	0.7	0	0	0
2	Rh/Al_2O_3 (0.01 equiv., 1 mol%, 0.62 mg)	0.02577	1.5702	0	0.0219	0	0	0	101.2	0	0.7	0	0	0
3	Rh/Al_2O_3 (0.01 equiv., 1 mol%, 0.62 mg)	0.02577	1.4277	0	0.0206	0	0	0	92.0	0	0.7	0	0	0
									96.1 ± 2.7	0 ± 0	0.7 ± 0	0 ± 0	0 ± 0	0 ± 0
4	Ni/SiO_2-Al_2O_3 (0.1 equiv., 10 mol%, 0.26 mg)	0.02577	1.5477	0	0.0161	0	0	0	99.7	0	0.5	0	0	0
5	Ni/SiO_2-Al_2O_3 (0.1 equiv., 10 mol%, 0.26 mg)	0.02577	1.5416	0.0075	0.0054	0	0	0	99.3	0.5	0.2	0	0	0
6	Ni/SiO_2-Al_2O_3 (0.1 equiv., 10 mol%, 0.26 mg)	0.02577	1.5573	0.0095	0.0027	0	0	0	100.3	0.6	0.1	0	0	0
									99.8 ± 0.3	0.4 ± 0.2	0.3 ± 0.1	0 ± 0	0 ± 0	0 ± 0
7	none	0.02577	1.4437	0.0116	0	0	0	0	93.0	0.7	0	0	0	0
8	none	0.02577	1.4719	0.0119	0	0	0	0	94.8	0.8	0	0	0	0
9	none	0.02577	1.4473	0.0122	0	0	0	0	93.2	0.8	0	0	0	0
									93.7 ± 0.6	0.8 ± 0	0 ± 0	0 ± 0	0 ± 0	0 ± 0
pH 7														
10	Rh/Al_2O_3 (0.01 equiv., 1 mol%, 0.62 mg)	0.01988	1.9439	0	0.0313	0	0	0	96.6	0	0.8	0	0	0
11	Rh/Al_2O_3 (0.01 equiv., 1 mol%, 0.62 mg)	0.01988	1.9246	0	0.0301	0	0	0	95.7	0	0.7	0	0	0
12	Rh/Al_2O_3 (0.01 equiv., 1 mol%, 0.62 mg)	0.01988	1.9693	0	0.0300	0	0	0	97.9	0	0.7	0	0	0
									96.7 ± 0.6	0 ± 0	0.8 ± 0	0 ± 0	0 ± 0	0 ± 0

			integrals relative to DMS (6H) set to 1.0000 at 3.0000 ppm						yield [%]					
#	catalyst loading (equiv., mol%, mg)	conc. DMS [M]	malate (2H)	fumarate (2H)	succi-nate (4H)	pyruvate (3H)	lactate (3H)	acetate (3H)	malate	fumarate	succi-nate	pyruvate	lactate	acetate
**13**	Ni/SiO_2-Al_2O_3 (0.1 equiv., 10 mol%, 0.26 mg)	0.01988	1.6268	0	0.0268	0	0	0	80.9	0	0.7	0	0	0
**14**	Ni/SiO_2-Al_2O_3 (0.1 equiv., 10 mol%, 0.26 mg)	0.01988	1.9991	0	0.0358	0	0	0	99.4	0	0.9	0	0	0
**15**	Ni/SiO_2-Al_2O_3 (0.1 equiv., 10 mol%, 0.26 mg)	0.01988	1.8979	0	0.0318	0	0	0	94.3	0	0.8	0	0	0
									91.5 ± 5.5	0 ± 0	0.8 ± 0.1	0 ± 0	0 ± 0	0 ± 0
**16**	none	19.88	1.9515	0.0141	0	0	0	0	97.0	0.7	0	0	0	0
**17**	none	19.88	1.9404	0.0170	0	0	0	0	96.4	0.8	0	0	0	0
**18**	none	19.88	1.9371	0.0173	0	0	0	0	96.3	0.9	0	0	0	0
									96.6 ± 0.2	0.8 ± 0.1	0 ± 0	0 ± 0	0 ± 0	0 ± 0

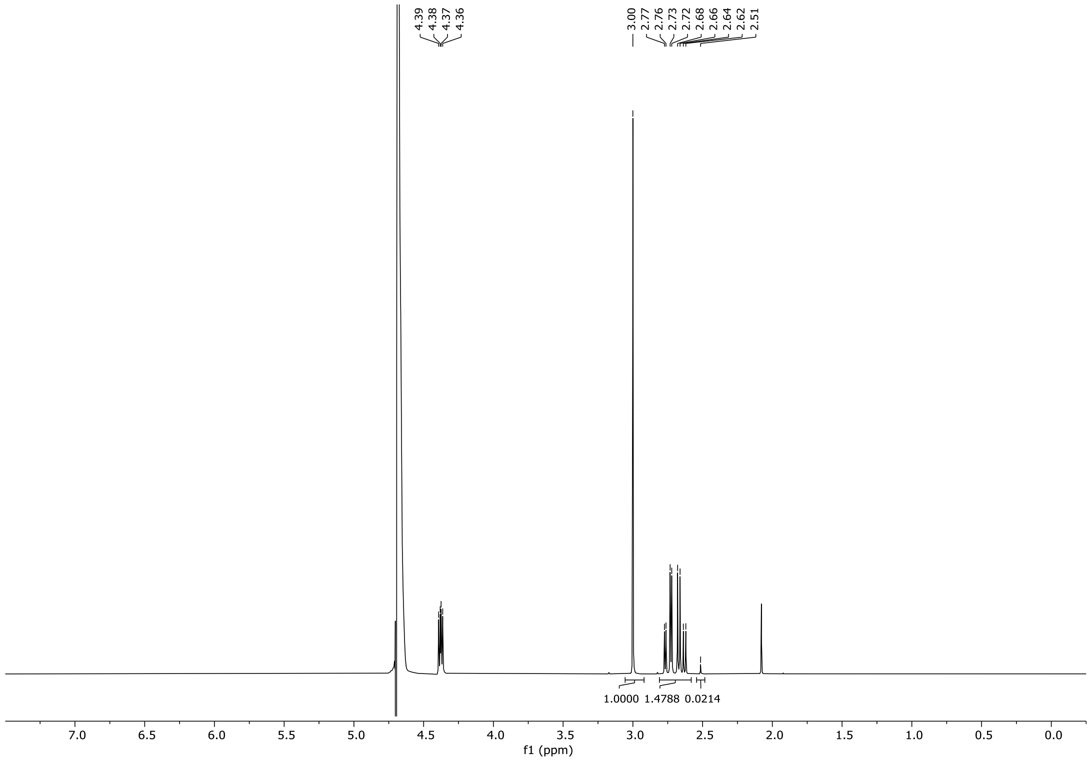

Figure S62. Representative ^{1}H qNMR using Rh/Al_2O_3 (1 mol%) as catalyst at pH 2 (Table S8 entry 1−3).

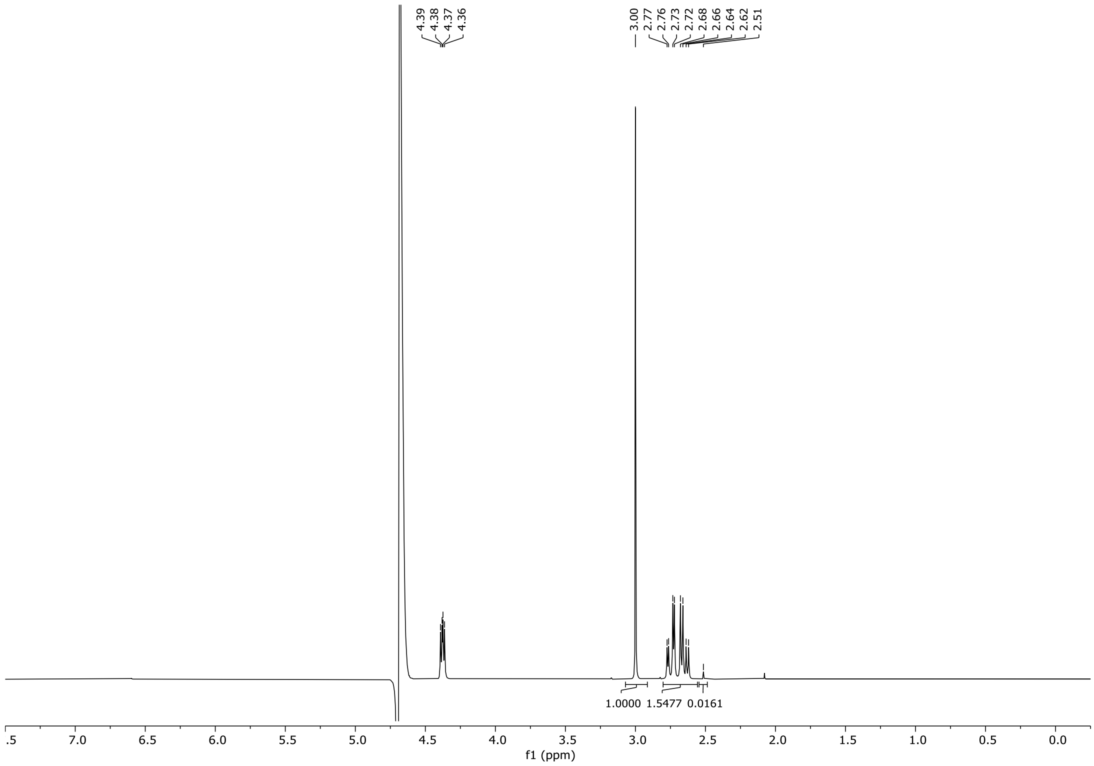

Figure S63. Representative ^{1}H qNMR using Ni/SiO_2-Al_2O_3 (10 mol%) as catalyst at pH 2 (Table S8 entry 4−6).

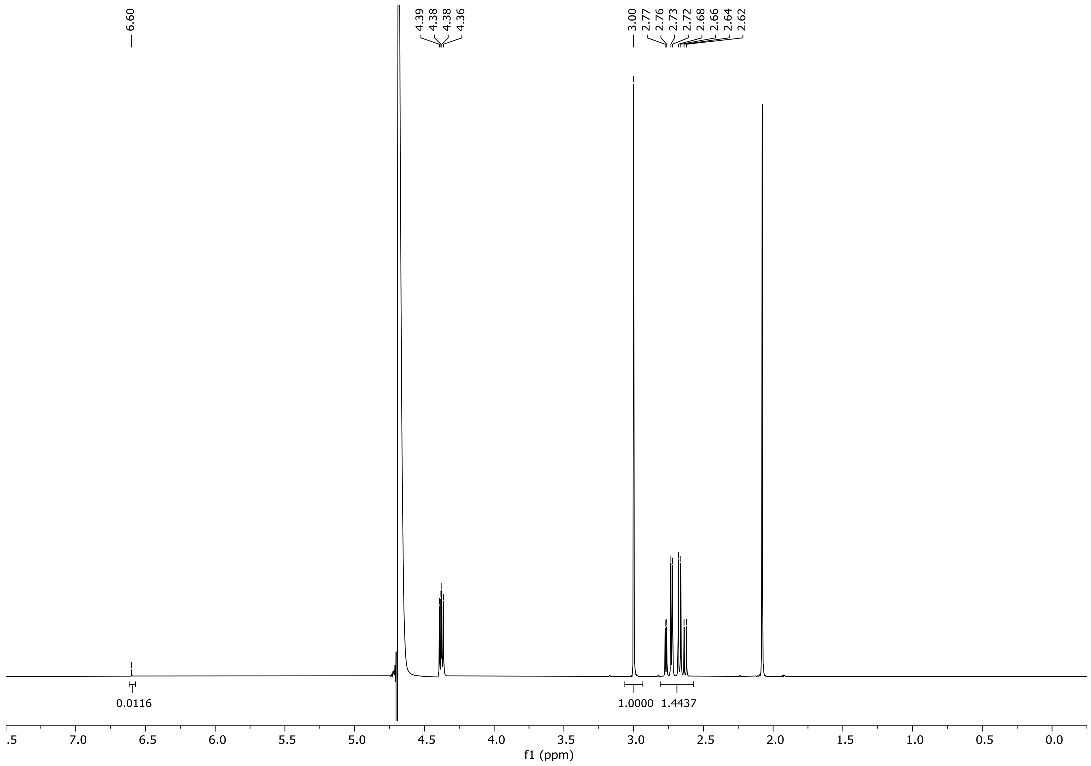

Figure S64. Representative ^{1}H qNMR without catalyst at pH 2 (Table S8 entry 7−9).

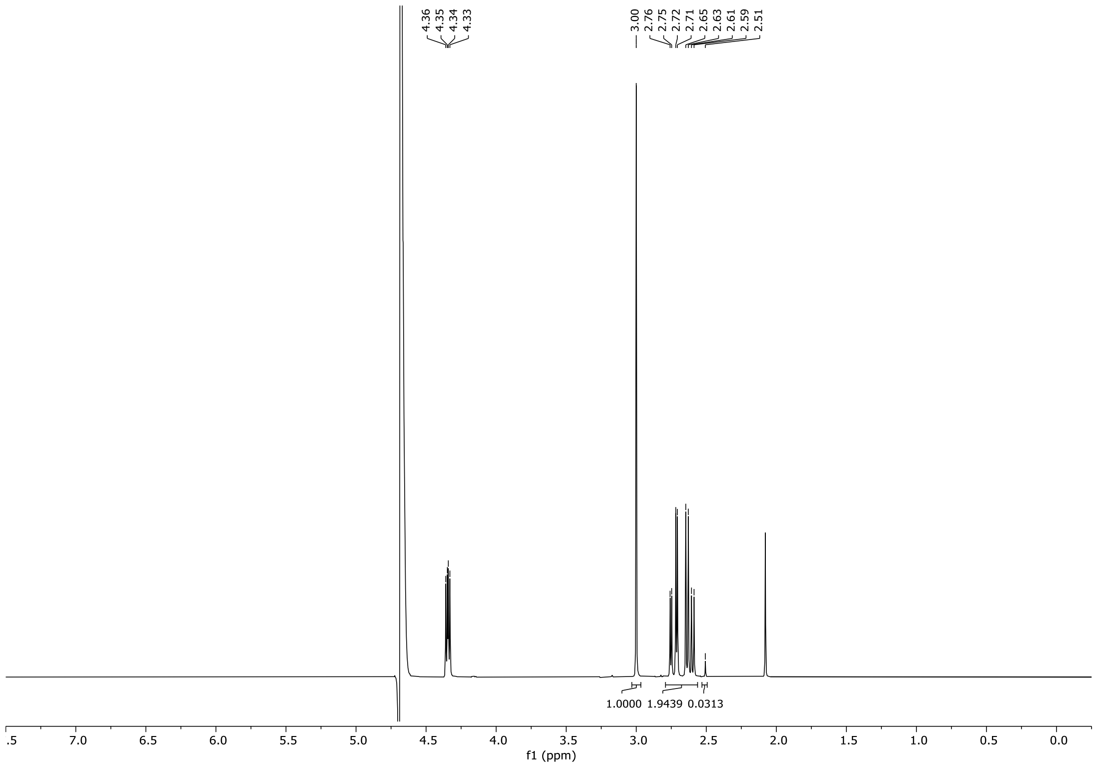

Figure S65. Representative ^{1}H qNMR using Rh/Al_2O_3 (1 mol%) as catalyst at pH 7 (Table S8 entry 10−12).

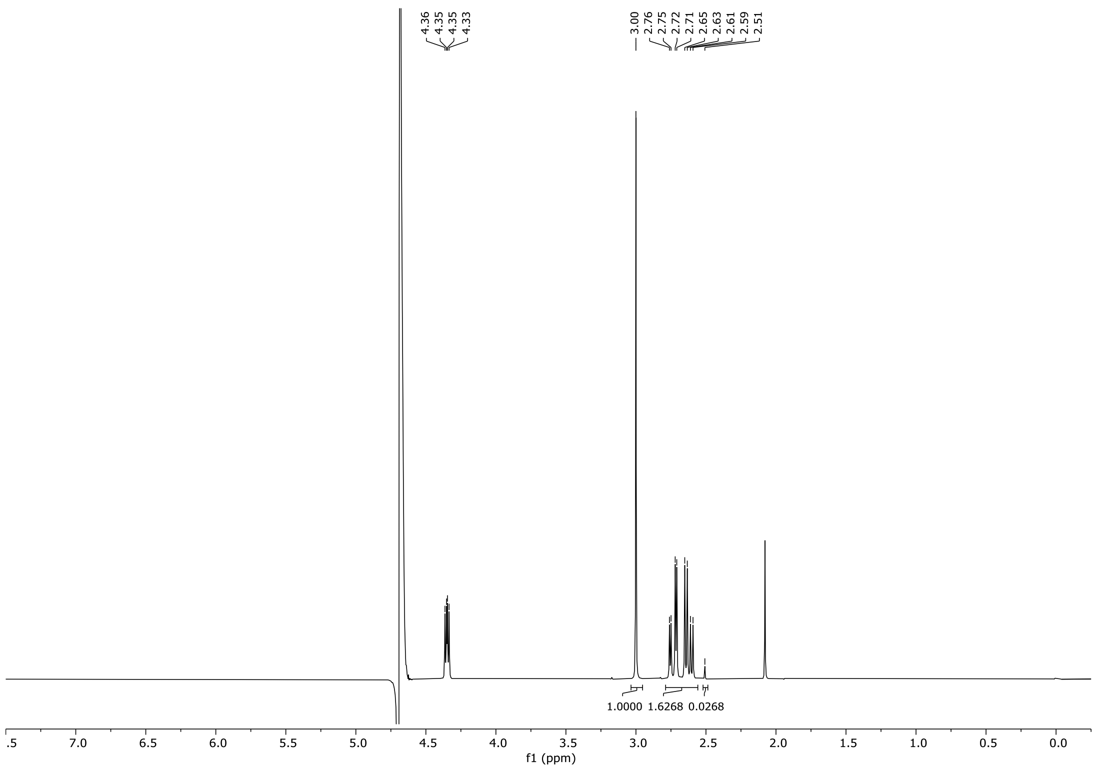

Figure S66. Representative ^{1}H qNMR using Ni/SiO_2-Al_2O_3 (10 mol%) as catalyst at pH 7 (Table S8 entry 13−15).

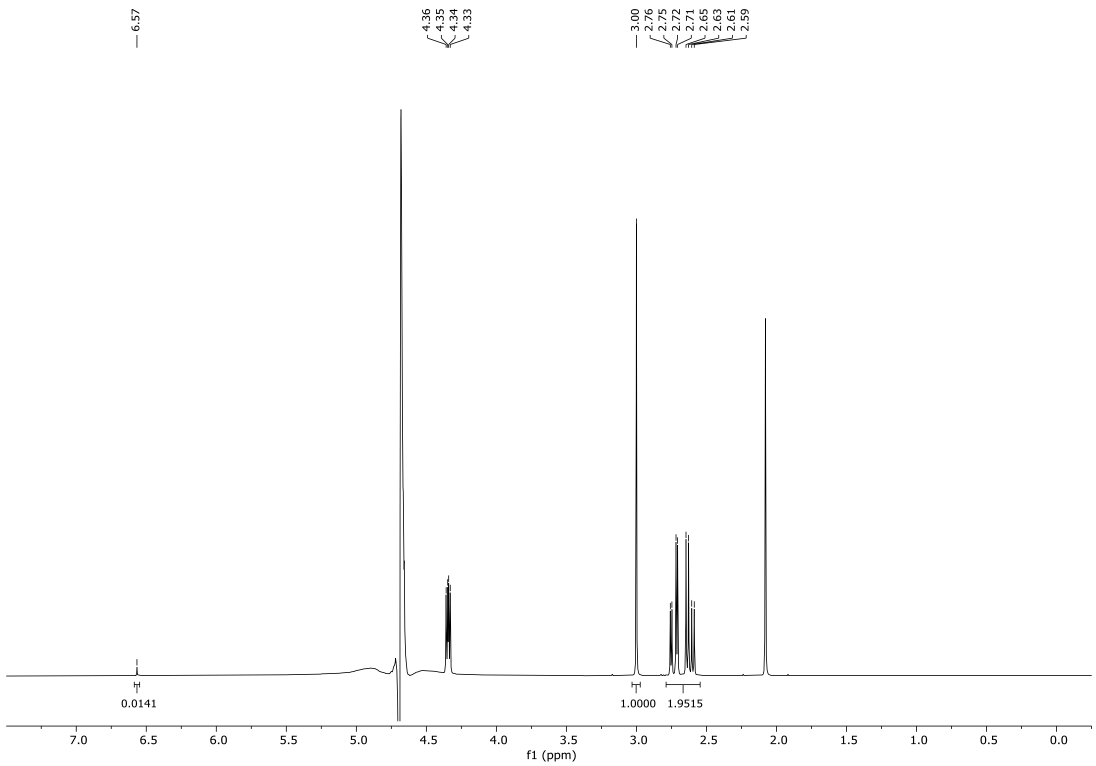

Figure S67. Representative ^{1}H qNMR without catalyst at pH 7 (Table S8 entry 16−18).

2.6 Reaction progress over time at 5 °C

A 25 mL flask with septum and PTFE stir bar was charged with catalyst (Rh/Al_2O_3 (0.01 equiv., 1 mol%, 9.26 mg) **or** Ni/SiO_2-Al_2O_3 (0.1 equiv., 10 mol%, 7.80 mg)), and oxaloacetic acid (0.45 mmol, 59.4 mg, 1 equiv.) dissolved in 15 mL degassed water (pH 2, unadjusted). After purging the flask for 5 min with H_2, the flask was equipped with an H_2 balloon and was stirred at 5 °C. Aliquots were collected at different timepoints and qNMRs were acquired according to section 1.4.2.

Table S9. Reaction conditions: oxaloacetic acid (30 mM), Rh/Al_2O_3 (0.01 equiv., 1 mol%, 9.26 mg), H_2 (1 atm), pH 7−8, 5 °C.

			integrals relative to DMS (6H) set to 1.0000 at 3.0000 ppm						yield [%]					
**#**	**time point (h)**	**conc. DMS [M]**	**oxalo-acetate (2H)**	**malate (2H)**	**succinate (4H)**	**pyruvate (3H)**	**lactate (3H)**	**acetate (3H)**	**oxalo-acetate**	**malate**	**succinate**	**pyruvate**	**lactate**	**acetate**
**1**	0	0.02027	0.8406	0[a]	0	0.4459	0	0.0169	85.2	0	0	15.1	0	0.6
**2**	1	0.02027	0.5492	0.2991[a]	0.0638	0.4883	0.0105	0.0204	55.7	30.3	1.6	16.5	0.4	0.7
**3**	2	0.02027	0.2447	0.4807	0.1098	0.6230	0.0216	0.0208	24.8	48.7	2.8	21.0	0.7	0.7
**4**	3.5	0.02027	0	1.4912	0.1916	0.2064	0.1325	0.0245	0	75.6	4.9	7.0	4.5	0.8
**5**	6	0.02027	0	1.5409	0.1957	0	0.3187	0.0267	0	78.1	5.0	0	10.8	0.9
**6**	9	0.02027	0	1.4907	0.1901	0	0.3083	0.0238	0	75.5	4.8	0	10.4	0.8
**7**	24	0.02027	0	1.5373	0.1953	0	0.3179	0.0225	0	77.9	4.9	0	10.7	0.8

[a] Quantification of malate by integration of one half of the dq from 2.60−2.66 ppm corresponding to one proton.

Table S10. Reaction conditions: oxaloacetic acid (30 mM), Ni/SiO_2-Al_2O_3 (0.1 equiv., 20 mol%, 7.80 mg), H_2 (1 atm), pH 7−8, 5 °C.

			integrals relative to DMS (6H) set to 1.0000 at 3.0000 ppm						yield [%]					
**#**	**time point (h)**	**conc. DMS [M]**	**oxalo-acetate (2H)**	**malate (2H)**	**succinate (4H)**	**pyruvate (3H)**	**lactate (3H)**	**acetate (3H)**	**oxalo-acetate**	**malate**	**succinate**	**pyruvate**	**lactate**	**acetate**
**1**	0	0.02027	0.6584	0[a]	0	1.0315	0	0.0165	66.7	0	0	34.8	0	0.6
**2**	1	0.02027	0.5114	0.0480[a]	0	0.9980	0	0.0133	51.8	4.9	0	33.7	0	0.4
**3**	9	0.02027	0.3000	0.1145[a]	0.0044	1.3375	0.0180	0.0208	30.4	11.6	0.1	45.2	0.6	0.7
**4**	24	0.02027	0	0.1154[a]	0.0045	1.7726	0.0185	0.0246	0	11.7	0.1	59.9	0.6	0.8
**5**	48	0.02027	0	0.1093[a]	0.0054	1.7629	0.0140	0.0191	0	11.1	0.1	59.6	0.5	0.6

[a] Quantification of malate by integration of one half of the dq from 2.60−2.66 ppm corresponding to one proton.

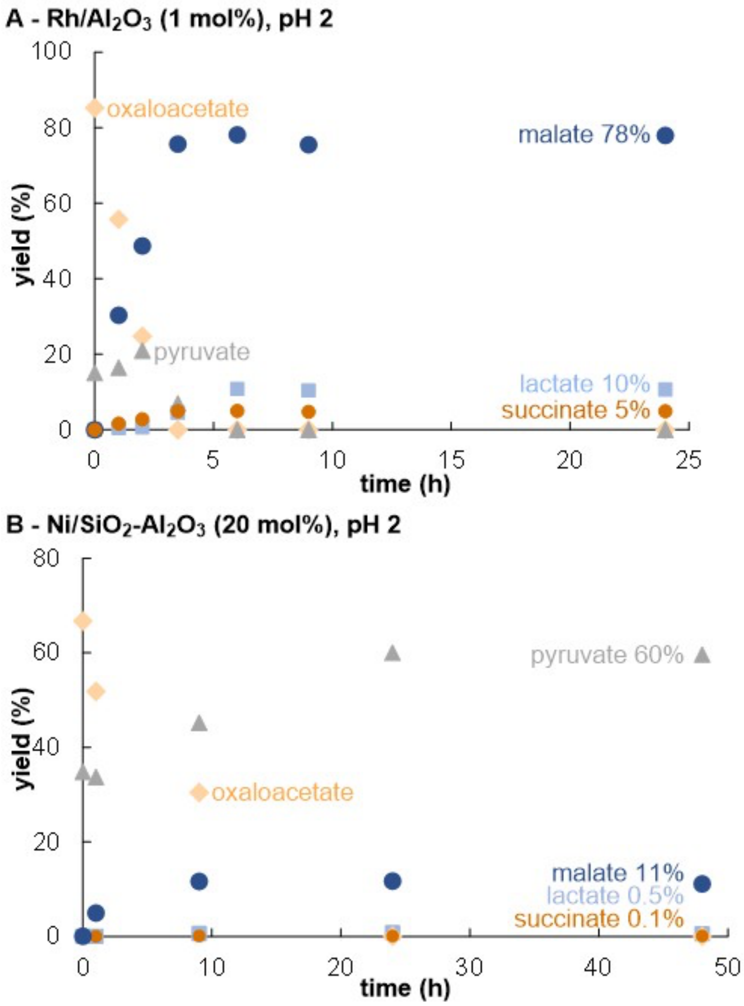

Figure S68. Reaction progress of oxaloacetate hydrogenation at 5 °C. Reaction conditions: 30 mM oxaloacetate, 15 mL H_2O (pH 2), 5 °C, H_2 (balloon) with **A** Rh/Al_2O_3 (1 mol%) and **B** $Ni/SiO_2{-}Al_2O_3$ (20 mol%), Yields were determined by quantitative ^1H NMR spectroscopy with dimethyl sulfone as an internal standard. In both cases, oxaloacetate is completely consumed after 48 h.

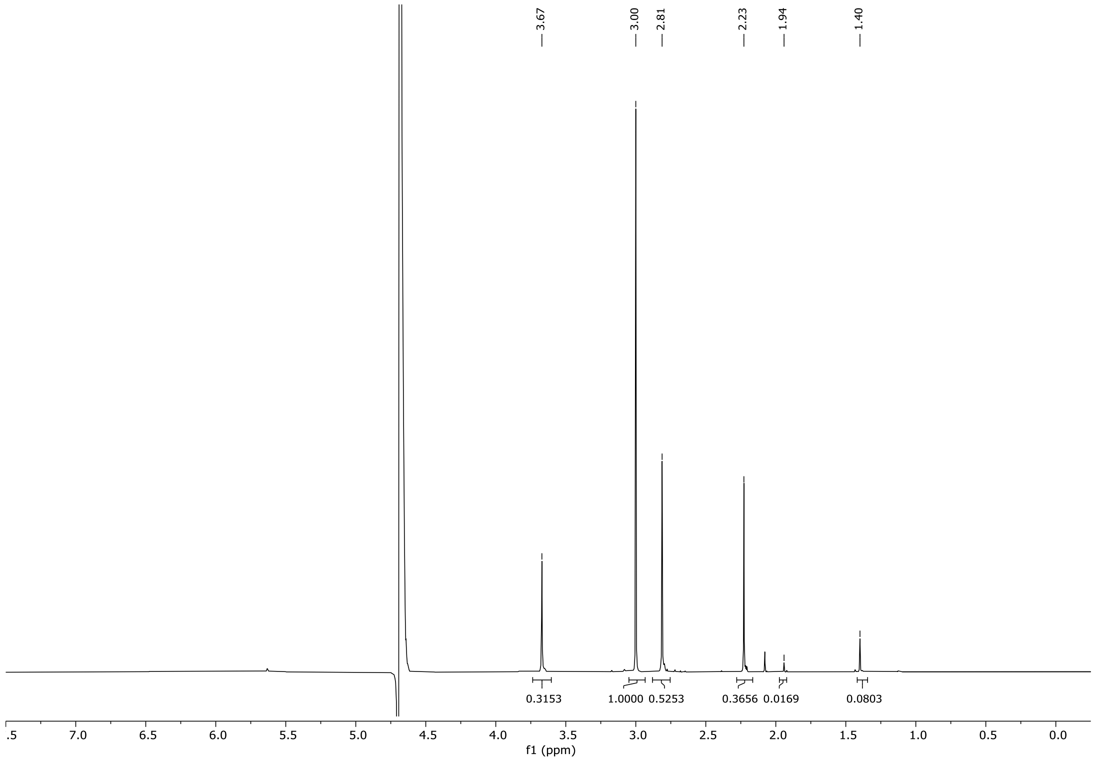

Figure S69. ^{1}H qNMR t = 0 h for Rh/Al_2O_3 (Table S9 entry 1).

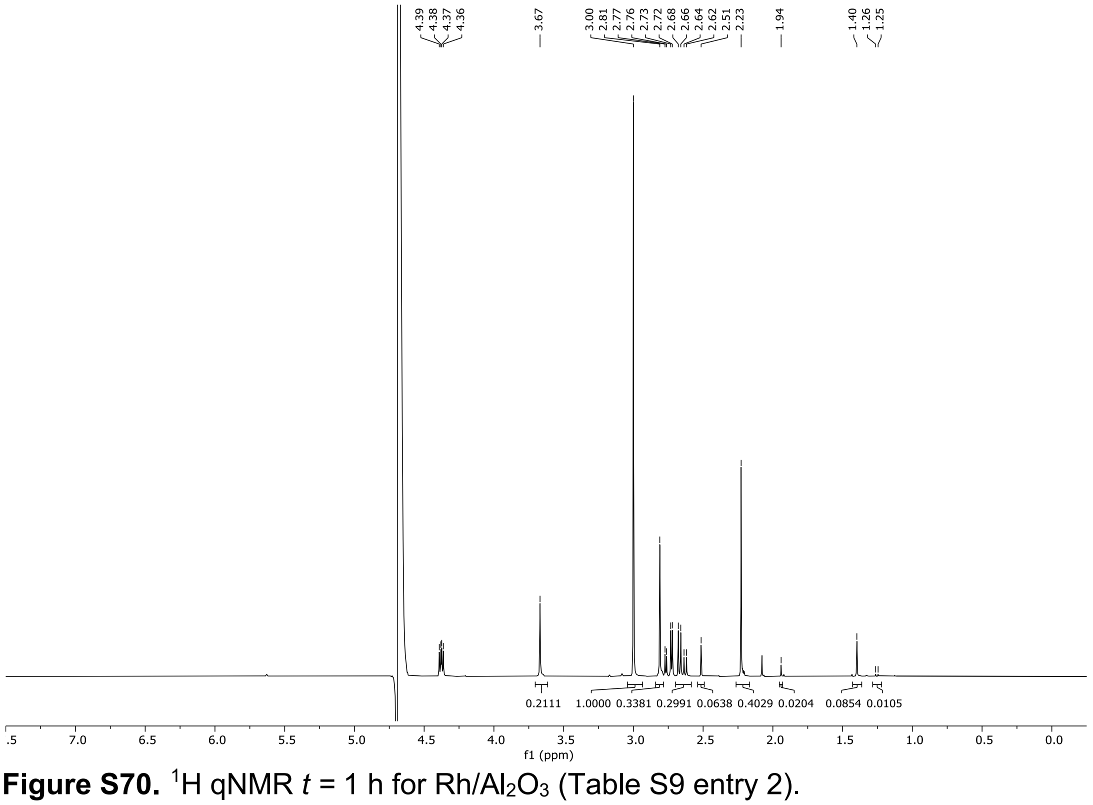

Figure S70. ^{1}H qNMR t = 1 h for Rh/Al_2O_3 (Table S9 entry 2).

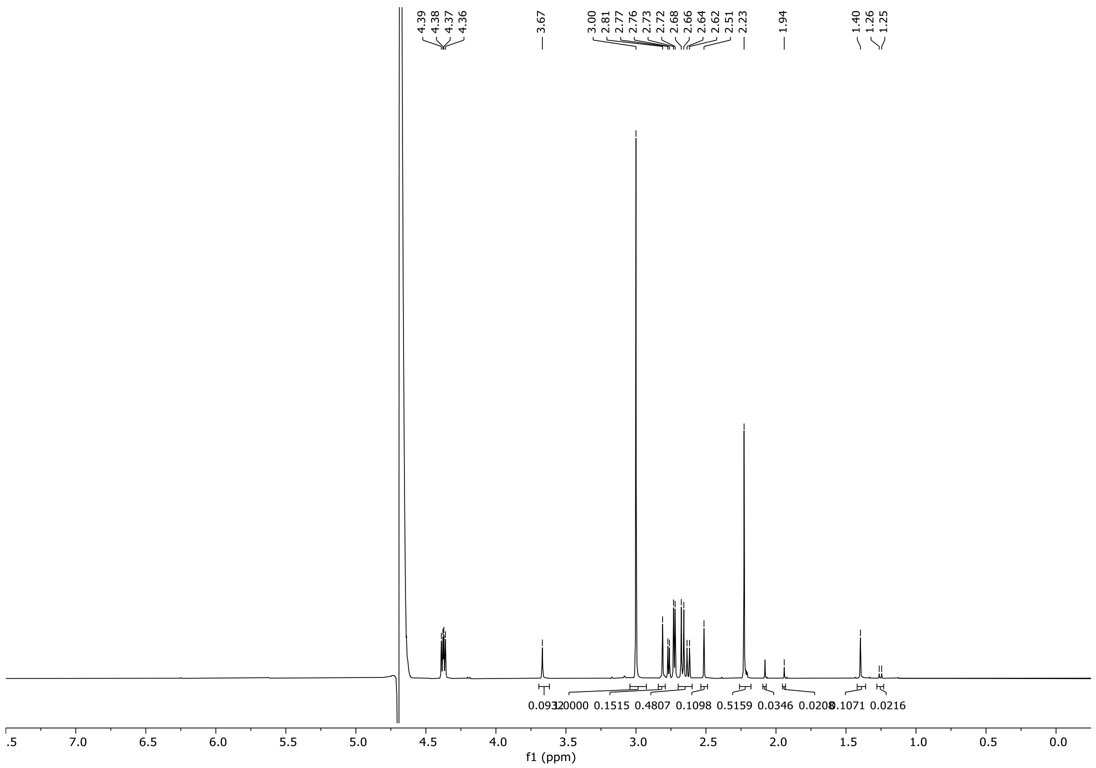

Figure S71. ^{1}H qNMR t = 2 h for Rh/Al_2O_3 (Table S9 entry 3).

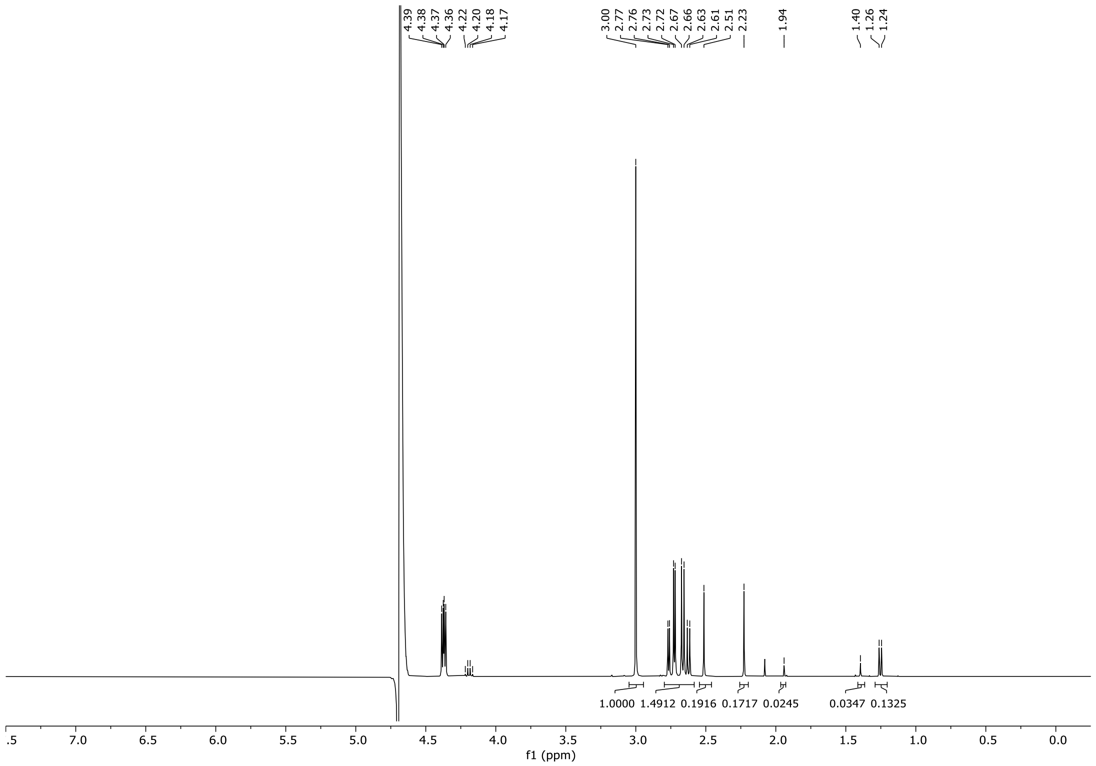

Figure S72. ^{1}H qNMR t = 3.5 h for Rh/Al_2O_3 (Table S9 entry 4).

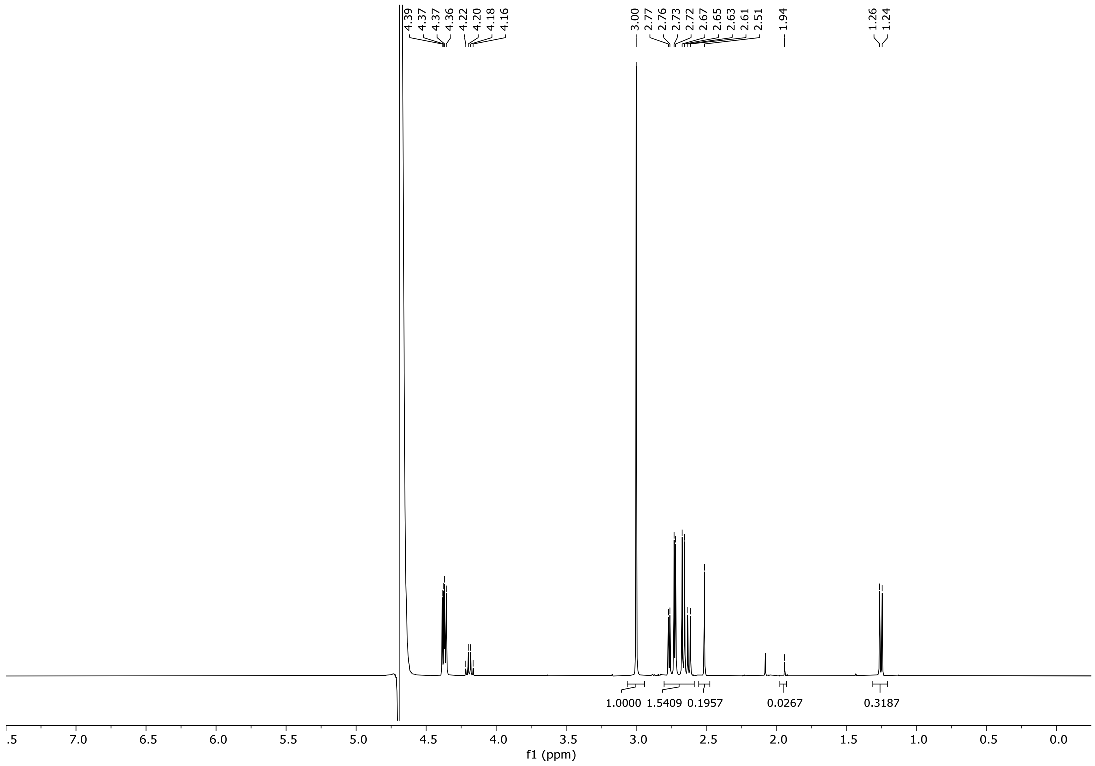

Figure S73. ^{1}H qNMR t = 6 h for Rh/Al_2O_3 (Table S9 entry 5).

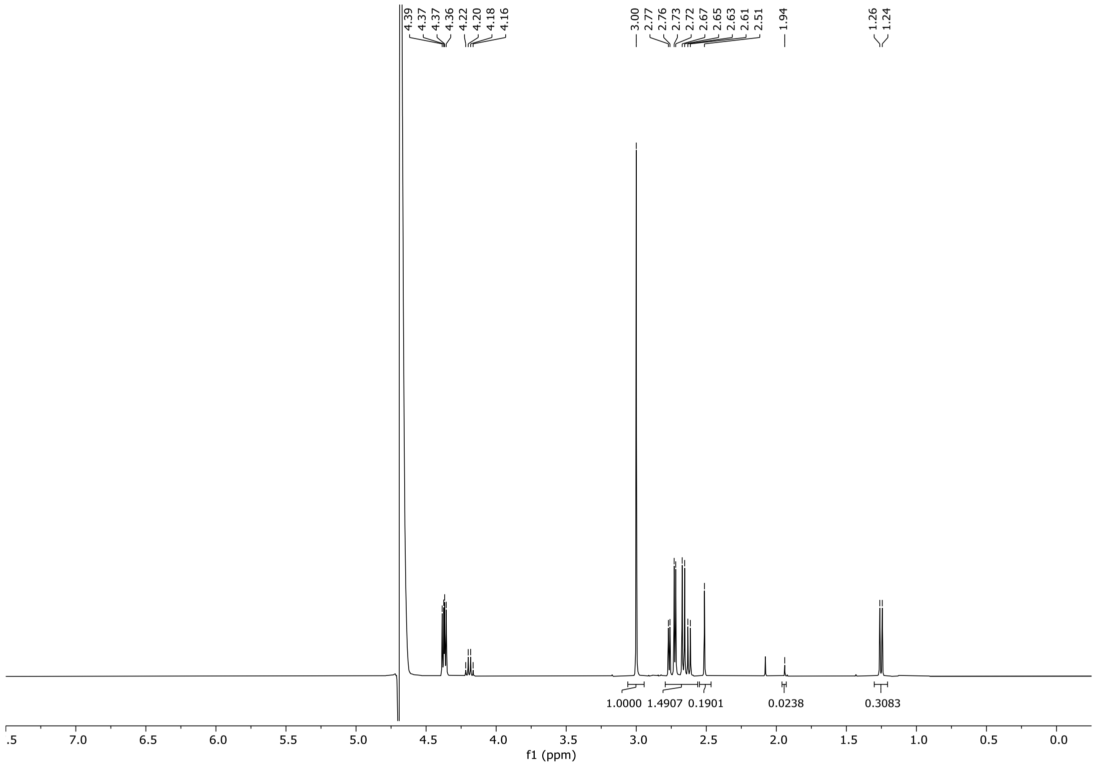

Figure S74. ^{1}H qNMR t = 9 h for Rh/Al_2O_3 (Table S9 entry 6).

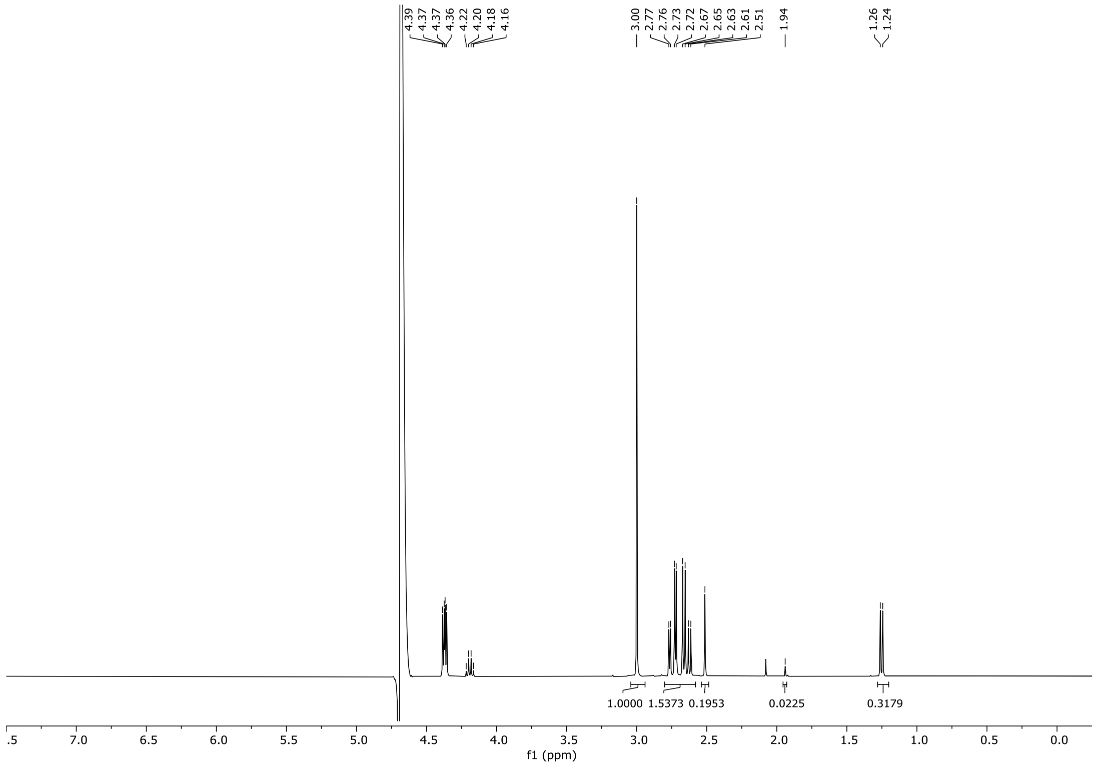

Figure S75. ^{1}H qNMR t = 24 h for Rh/Al_2O_3 (Table S9 entry 7).

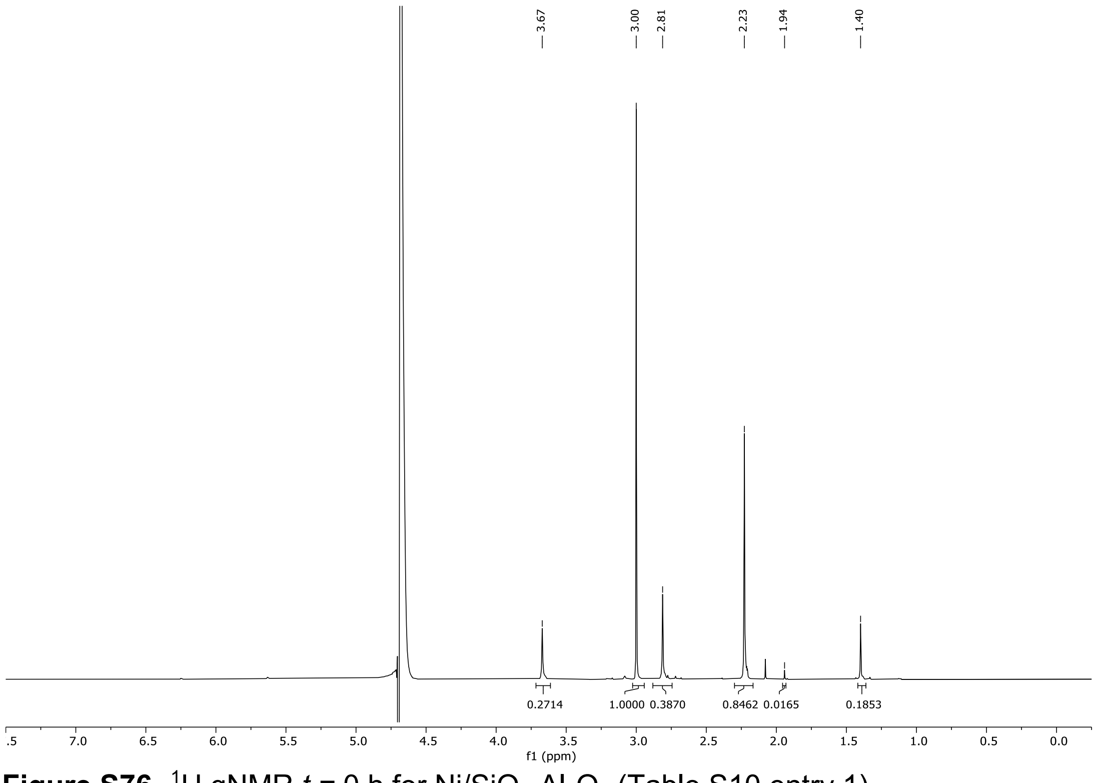

Figure S76. ^{1}H qNMR t = 0 h for Ni/SiO_2-Al_2O_3 (Table S10 entry 1).

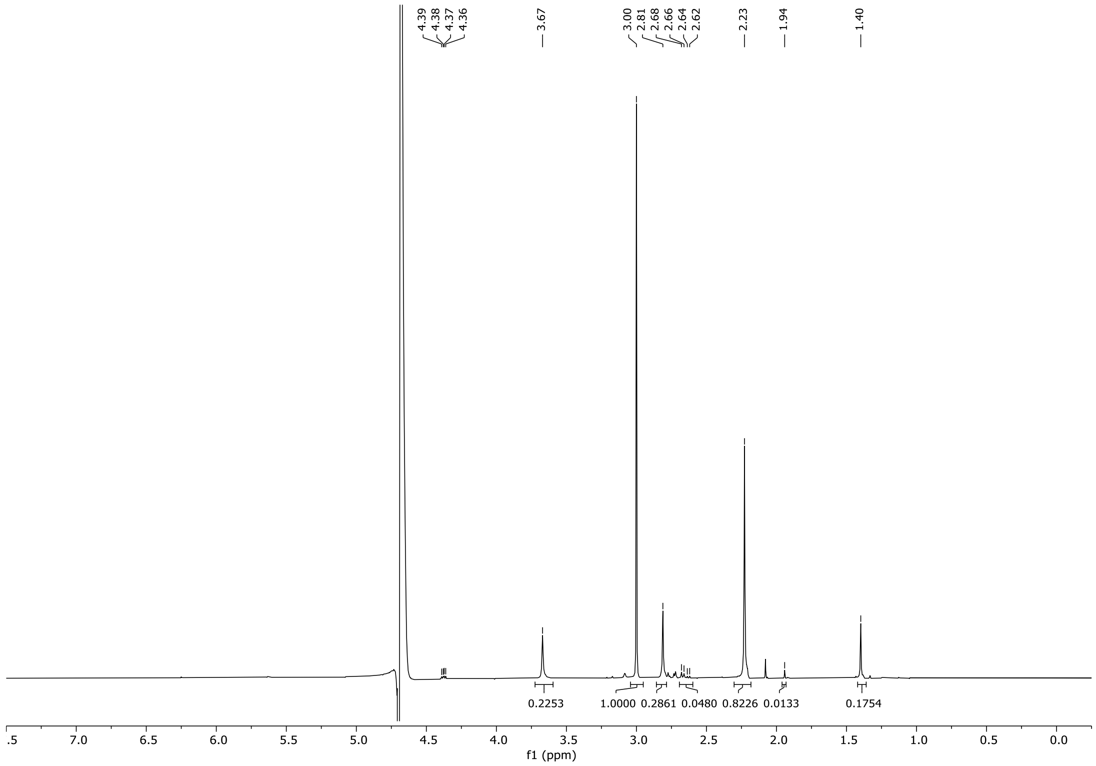

Figure S77. ^{1}H qNMR *t* = 1 h for Ni/SiO_2-Al_2O_3 (Table S10 entry 2).

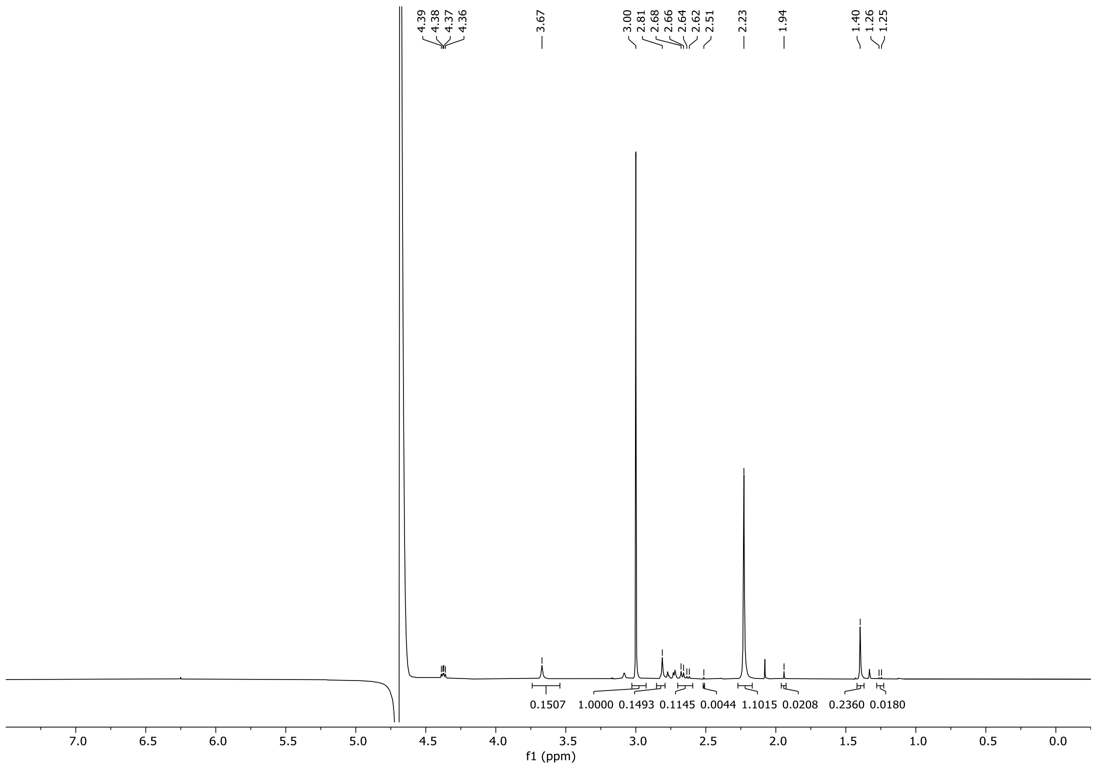

Figure S78. ^{1}H qNMR *t* = 9 h for Ni/SiO_2-Al_2O_3 (Table S10 entry 3).

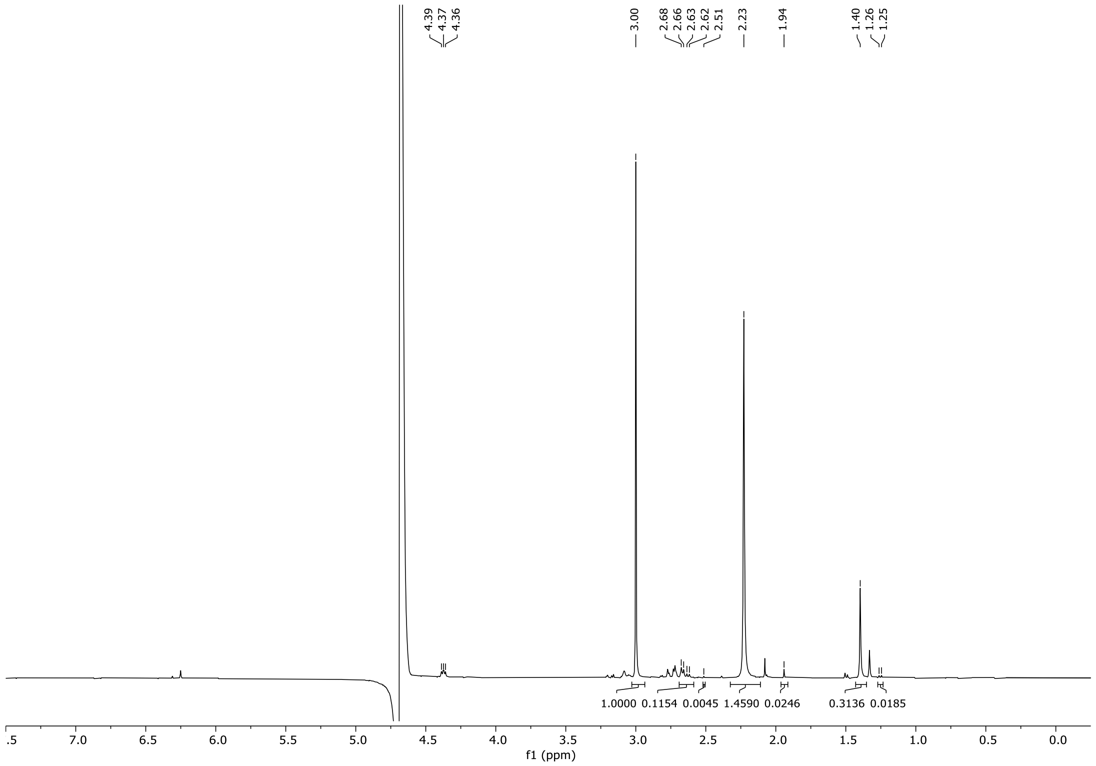

Figure S79. ^{1}H qNMR t = 24 h for Ni/SiO_2-Al_2O_3 (Table S10 entry 4).

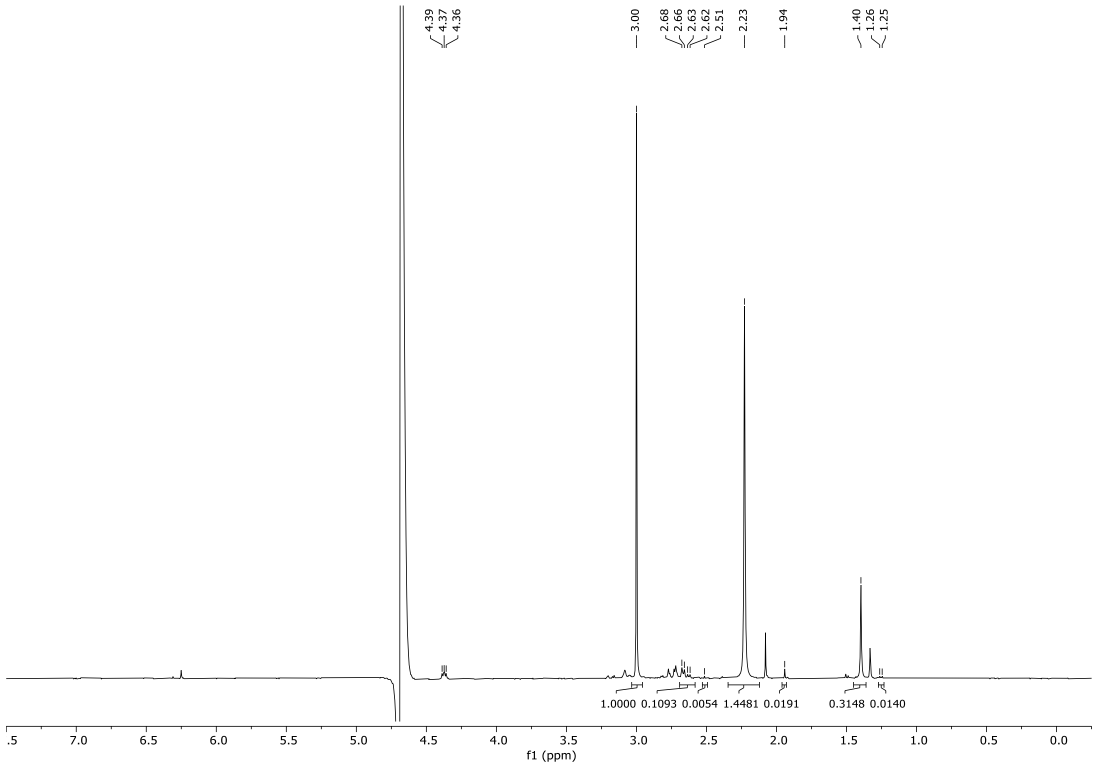

Figure S80. ^{1}H qNMR t = 48 h for Ni/SiO_2-Al_2O_3 (Table S10 entry 5).

2.7 Control reactions

2.7.1 Argon atmosphere

To demonstrate that hydrogen is necessary as reducing agent, reactions under argon atmosphere under otherwise unchanged standard conditions were performed. Therefore, a disposable glass vial with PTFE/silicone-lined septum (see section 1.2 for more details) was charged with the metal catalyst (Rh/Al_2O_3, 0.01 equiv., 1 mol%, 0.62 mg **or** Ni/SiO_2-Al_2O_3, 0.1 equiv., 10 mol%, 0.26 mg). Then, oxaloacetic acid (0.03 mmol, 3.96 mg, 1 equiv.) was added from an aqueous stock solution (0.06 M in water and adjusted to pH 7−8 with NaOH) and water was added to a total volume of 1 mL solvent. After purging the vials for 3 min with argon, the vial was equipped with an argon balloon and was stirred at 22 °C for 18 h. Experiments were done in triplicates if not otherwise noted. qNMRs were acquired according to section 1.4.2. Mean values of yields and standard errors were calculated for each set of reactions.

Table S11. Reaction conditions: oxaloacetic acid (30 mM), Rh/Al_2O_3 (1 mol%) **or** Ni/SiO2-Al_2O_3 (10 mol%), argon (1 atm), pH 7−8, 22 °C, 18 h.

			integrals relative to DMS (6H) set to 1.0000 at 3.0000 ppm						yield [%]					
**#**	**catalyst loading (equiv., mol%, mg)**	**conc. DMS [M]**	**oxalo-acetate (2H)**	**malate (2H)**	**succi-nate (4H)**	**pyruvate (3H)**	**lactate (3H)**	**acetate (3H)**	**oxalo-acetate**	**malat e**	**succi-nate**	**pyruvate**	**lactate**	**acetate**
**1**	Rh/Al_2O_3 (0.01 equiv., 1 mol%, 0.62 mg)	0.02221	0.0167	0	0	2.6216	0	0.0195	1.9	0	0	97.0	0	0.7
**2**	Rh/Al_2O_3 (0.01 equiv., 1 mol%, 0.62 mg)	0.02221	0.0152	0	0	2.5981	0	0.0184	1.7	0	0	96.2	0	0.7
**3**	Rh/Al_2O_3 (0.01 equiv., 1 mol%, 0.62 mg)	0.02221	0	0	0	2.5550	0	0.0192	0	0	0	94.6	0	0.7
									1.2 ± 0.6	0 ± 0	0 ± 0	95.9 ± 0.7	0 ± 0	0.7 ± 0
**4**	Ni/SiO_2-Al_2O_3 (0.1 equiv., 10 mol%, 0.26 mg)	0.02221	0	0	0	2.0825	0	0.0302	0	0	0	77.1	0	1.1
**5**	Ni/SiO_2-Al_2O_3 (0.1 equiv., 10 mol%, 0.26 mg)	0.02221	0	0	0	2.0876	0	0.0354	0	0	0	77.3	0	1.3
**6**	Ni/SiO_2-Al_2O_3 (0.1 equiv., 10 mol%, 0.26 mg)	0.02221	0	0	0	2.0370	0	0.0354	0	0	0	75.4	0	1.3
									0 ± 0	0 ± 0	0 ± 0	76.6 ± 0.6	0 ± 0	1.2 ± 0.1

No reduction product was observed for both catalysts. Small amounts of starting material are left in the case of Rh/Al_2O_3, whereas with Ni/SiO_2-Al_2O_3 oxaloacetate decarboxylated completely to pyruvate.

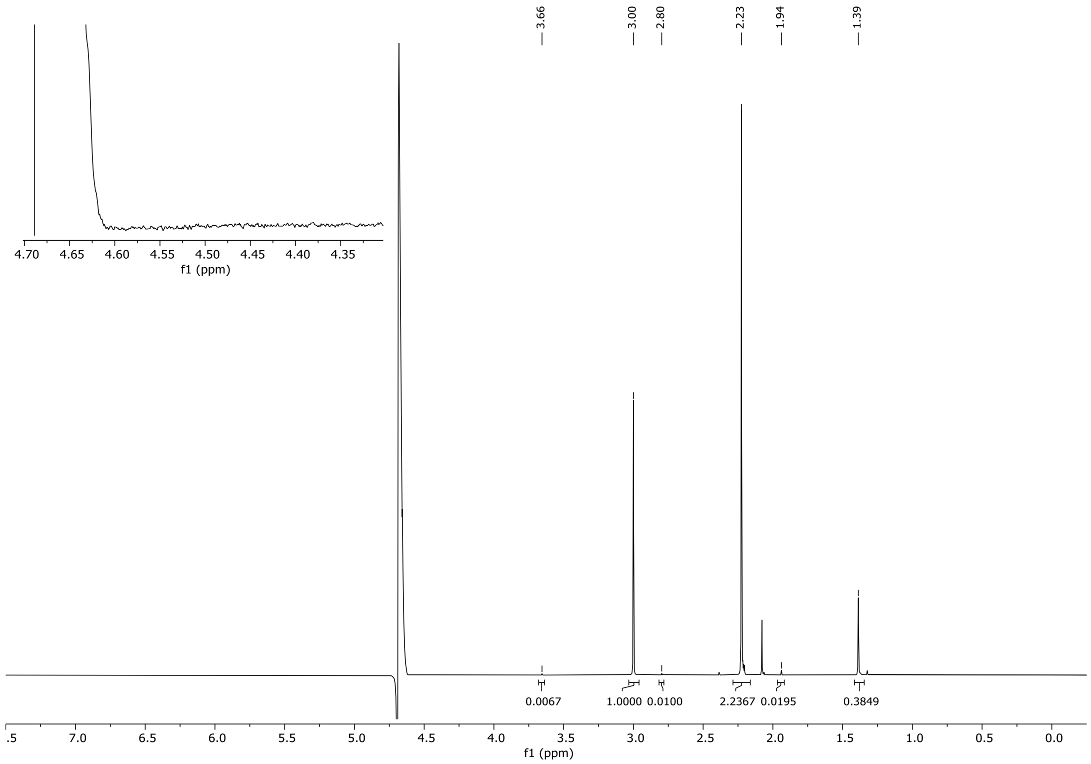

Figure S81. Representative ^{1}H qNMR using Rh/Al_2O_3 as catalyst (Table S11 entry 1−3).

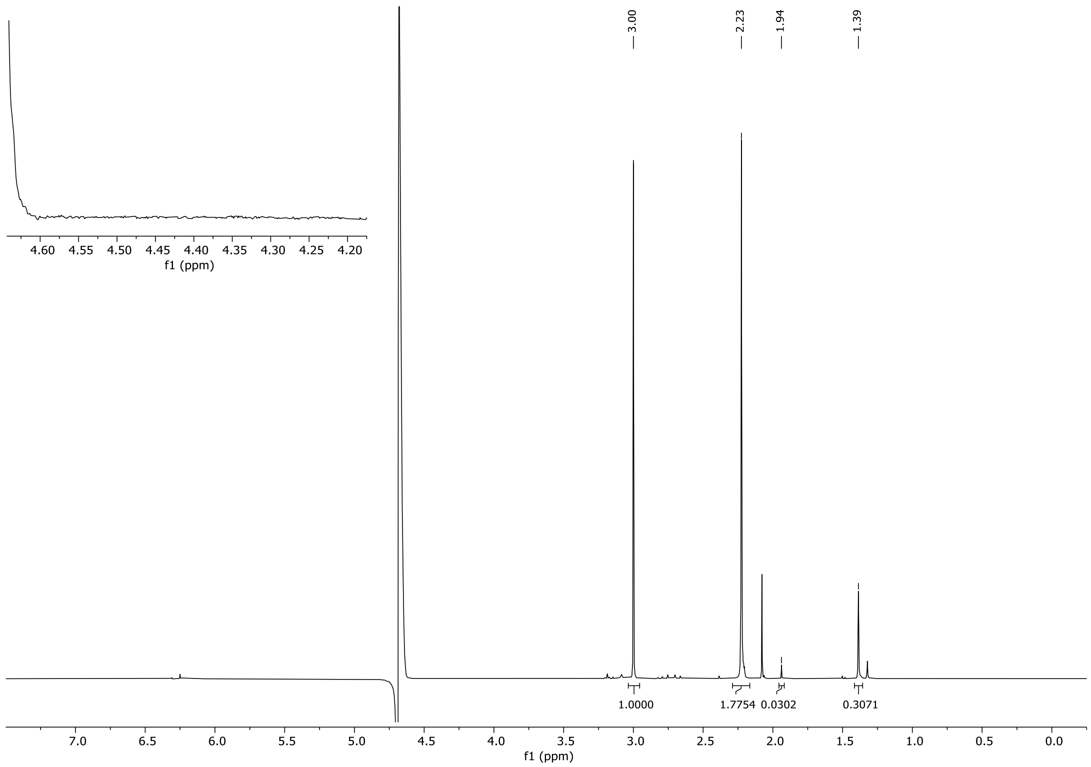

Figure S82. Representative ^{1}H qNMR using Ni/SiO_2-Al_2O_3 as catalyst (Table S11 entry 4−6).

2.7.2 Support without metal

To exclude the possibility that the support has reducing properties and to demonstrate that a metal is responsible for the catalytic reduction, reactions were performed with only the support materials. Therefore, a disposable glass vial with PTFE/silicone-lined septum (see section 1.2 for more details) was charged with the support material (Al_2O_3, 0.2 equiv., 20 mol%, 0.61 mg **or** SiO_2-Al_2O_3, 0.2 equiv., 20 mol%, 1.7 mg). Then, oxaloacetic acid (0.03 mmol, 3.96 mg, 1 equiv.) was added from an aqueous stock solution (0.06 M in water and adjusted to pH 7−8 with NaOH) and water was added to a total volume of 1 mL solvent. After purging the vials for 3 min with H_2, the vial was equipped with an H_2 balloon and was stirred at 22 °C for 18 h. Experiments were done in triplicates if not otherwise noted. qNMRs were acquired according to section 1.4.2. Mean values of yields and standard errors were calculated for each set of reactions.

Table S12. Reaction conditions: oxaloacetic acid (30 mM), Al_2O_3 (0.2 equiv., 20 mol%, 0.61 mg) **or** SiO_2-Al_2O_3 (0.2 equiv., 20 mol%, 1.7 mg), H_2 (1 atm), pH 7−8, 22 °C, 18 h.

			integrals relative to DMS (6H) set to 1.0000 at 3.0000 ppm						yield [%]					
**#**	**support material**	**conc. DMS [M]**	**oxalo-acetate (2H)**	**malate (2H)**	**succi-nate (4H)**	**pyruvate (3H)**	**lactate (3H)**	**acetate (3H)**	**oxalo-acetate**	**malate**	**succi-nate**	**pyruvate**	**lactate**	**acetate**
**1**	Al_2O_3 (0.2 equiv., 20 mol%, 0.61 mg)	0.02577	0	0	0	2.1112	0	0.0144	0	0	0	90.7	0	0.6
**2**	Al_2O_3 (0.2 equiv., 20 mol%, 0.61 mg)	0.02577	0	0	0	2.0625	0	0.0151	0	0	0	88.6	0	0.6
									0 ± 0	0 ± 0	0 ± 0	89.6 ± 1.0	0 ± 0	0.6 ± 0
**3**	SiO_2-Al_2O_3 (0.2 equiv., 20 mol%, 1.7 mg)	0.02577	0	0	0	1.6547	0	0.0251	0	0	0	71.1	0	1.1
**4**	SiO_2-Al_2O_3 (0.2 equiv., 20 mol%, 1.7 mg)	0.02577	0	0	0	1.7271	0	0.0159	0	0	0	74.2	0	0.7
									0 ± 0	0 ± 0	0 ± 0	72.6 ± 1.6	0 ± 0	0.9 ± 0.2

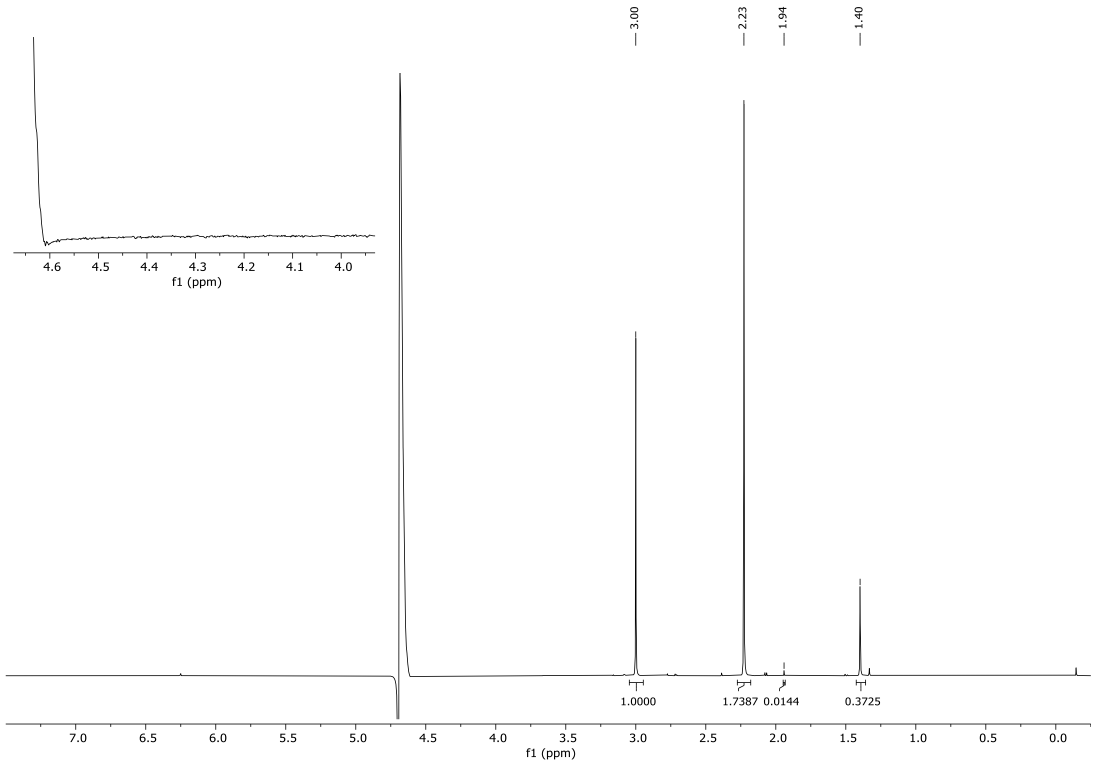

Figure S83. Representative ^{1}H qNMR for Al_2O_3 (Table S12 entry 1+2).

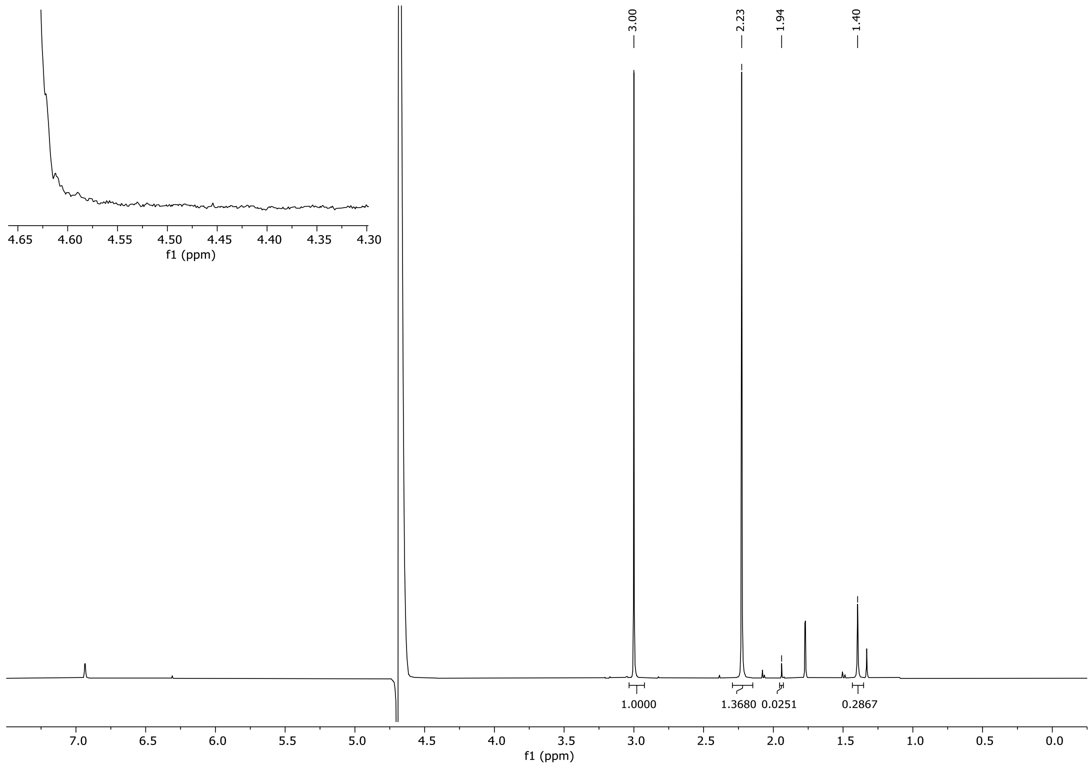

Figure S84. Representative ^{1}H qNMR for SiO_2-Al_2O_3 (Table S12 entry 3+4).

2.8 Reaction with meteorites

2.8.1 Classification and composition of iron meteorites

Table S13. Characteristics and composition of investigated iron meteorites.

#	meteorite	location	year	mass	classification	Composition (%)				Composition (ppm)			
						Ni	Co	P	S	Ga	Ge	Ir	Rh[1]
1[2]	Campo del Cielo	Argentina	1576	50 t	iron, IAB-MG	6.68	0.43	0.25	-	87	407	3.6	2.39
2[3]	Gibeon	Namibia	1836	26 t	iron, IVA	7.93	0.41	0.04	-	2.0	0.12	2.3	1.16
3[4]	Sikhote Alin	Russia	1947	23 t	iron, IIAB	5.90	0.42	0.46	0.28	52	161	0.03	2.21

The meteorites in the table above consist mainly of iron. In addition to the elements listed, which are used to classify the iron meteorites, other elements such as C, Cu, As, Zn, W, Mo, Au, Os, Ru, Pd, Pt.[5] The total mass of the meteorite is given in Table S13. However, when the meteorites enter the Earth's atmosphere, they shatter into pieces of different sizes, which can be spread over a scattering field of several square kilometers on the Earth. It should be noted that the composition between pieces of the same meteorite and in a meteorite piece itself is not homogeneously distributed and the numbers given may vary slightly depending on the reference.

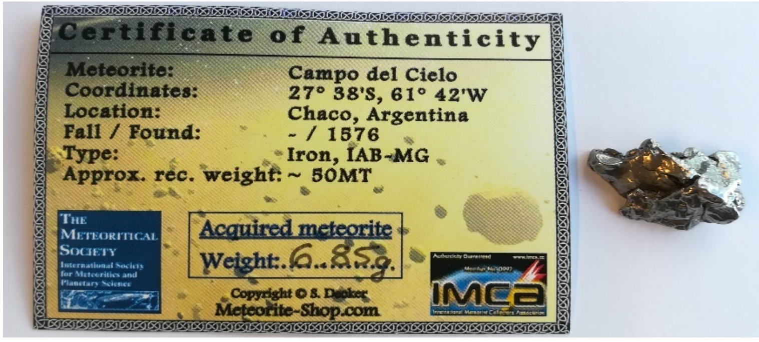

Figure S 85. Campo del Cielo meteorite.

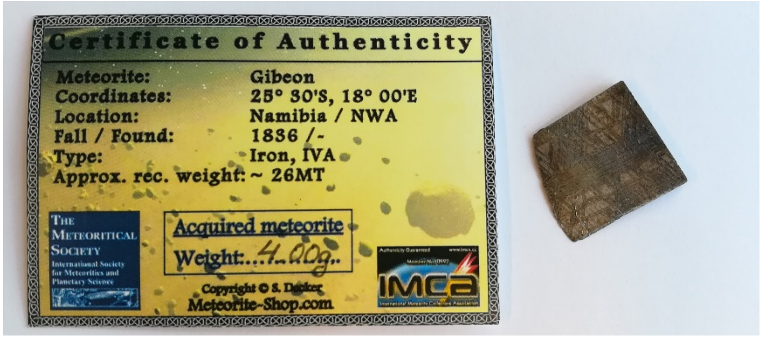

Figure S 86. Gibeon meteorite.

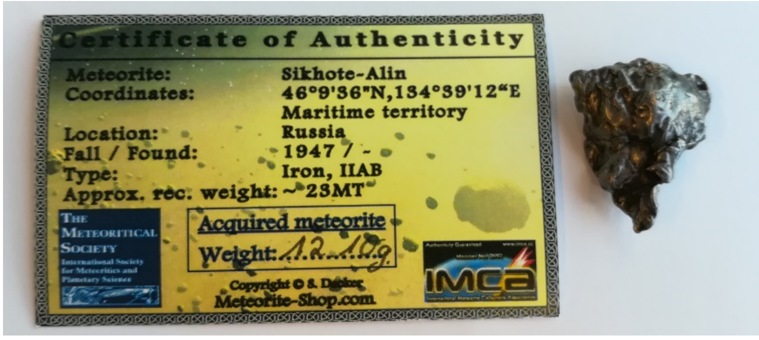

Figure S87. Sikhote Alin meteorite.

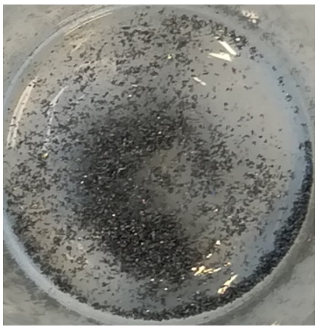
Figure S88. Meteorite powder, here of the Gibeon meteorite, used in the reaction.

2.8.2 Meteorite screen for the reduction of oxaloacetate at 5 bar hydrogen

A disposable glass vial with pierced PTFE/silicone-lined septum (see section 1.2 for more details) was charged with fine meteorite chips (10 mg or 0.5 mg) or metal powder (10 mg). Then, oxaloacetic acid (0.03 mmol, 3.96 mg, 1 equiv.) was added from an aqueous stock solution (0.06 M adjusted to pH 7−8 with NaOH) and water was added to a total volume of 1 mL solvent. After purging the three times with H_2, the pressure reactor was pressurized with 5 bar H_2 and stirred at 22 °C for 18 h. Experiments were done in duplicates. qNMRs were acquired according to section 1.4.2. Mean values of yields and standard errors were calculated for each set of reactions.

Table S14. Reaction conditions: oxaloacetic acid (30 mM), meteorite or metal powder (10 mg or 0.5 mg), H_2 (5 bar), pH 7−8, 22 °C, 18 h.

			integrals relative to DMS (6H) set to 1.0000 at 3.0000 ppm						yield [%]					
**#**	**catalyst loading (mg)**	**conc. DMS [M]**	**oxalo-acetate (2H)**	**malate (2H)**	**succi-nate (4H)**	**pyruvate (3H)**	**lactate (3H)**	**acetate (3H)**	**oxalo-acetate**	**malate**	**succi-nate**	**pyruvate**	**lactate**	**acetate**
**10 mg**														
**1**[a,b]	Campo del Cielo (10 mg)	0.01815	0.0976	0.2669	0	1.4708	0.3662	0.0249	8.9	12.1	0	44.5	11.1	0.8
**2**[a,b]	Campo del Cielo (10 mg)	0.01815	0.1681	0.3617	0	1.1001	0.4776	0.0220	15.3	16.4	0	33.3	14.4	0.7
									12.1 ± 3.2	14.8 ± 2.7	0 ± 0	40.0 ± 4.5	13.3 ± 2.2	0.7 ± 0
**3**[a,b]	Gibeon (10 mg)	0.01815	0.0717	0.4087	0	1.3133	0.4083	0.0251	6.5	18.5	0	39.7	12.4	0.8
**4**[a,b]	Gibeon (10 mg)	0.01815	0.1797	0.4229	0	1.0048	0.3903	0.0226	16.3	19.2	0	30.4	11.8	0.7
									11.4 ± 4.9	18.9 ± 0.3	0 ± 0	35.1 ± 4.7	12.1 ± 0.3	0.7 ± 0
**5**[a,b]	Sikhote Alin (10 mg)	0.01815	0.0121	0.3561	0	1.5095	0.4077	0.0304	1.1	16.2	0	45.7	12.3	0.9
**6**[a,b]	Sikhote Alin (10 mg)	0.01815	0.0067	0.2642	0	1.4197	0.4266	0.0276	0.6	12.0	0	42.9	12.9	0.8
									0.9 ± 0.2	14.1 ± 2.1	0 ± 0	44.3 ± 1.4	12.6 ± 0.3	0.9 ± 0
**7**	Ni^0 (10 mg)	0.01815	0.0016	1.0739	0	0.6911	0.4877	0.0220	0.1	48.7	0	20.9	14.8	0.7
**8**	Ni^0 (10 mg)	0.01815	0.0047	0.8992	0	0.7505	0.4921	0.0214	0.4	40.8	0	22.7	14.9	0.6
									0.3 ± 0.1	44.8 ± 4.0	0 ± 0	21.8 ± 0.9	14.8 ± 0.1	0.7 ± 0
**9**	none	0.01815	0.0695	0	0	2.4910	0	0.0194	6.3	0	0	75.4	0	0.6
**10**	none	0.01815	0.0565	0	0	2.5979	0	0.0212	5.1	0	0	78.6	0	0.6
									5.7 ± 0.6	0 ± 0	0 ± 0	77.0 ± 1.6	0 ± 0	0.6 ± 0
**0.5 mg**														
**11**	Gibeon (0.5 mg)	0.01815	0.4024	0.0116	0	1.5973	0.0385	0.0257	36.5	1.1	0	48.3	1.2	0.8
**12**	Gibeon (0.5 mg)	0.01815	0.3915	0.0024	0	1.6358	0.0315	0.0275	35.5	0.1	0	49.5	1.0	0.8
									36.0 ± 0.5	0.6 ± 0.5	0 ± 0	48.9 ± 0.6	1.1 ± 0.1	0.8 ± 0

[a] malonate was observed in traces [b] formate was observed in traces

Fe^0 (10 mg) did not enable the reduction of oxaloacetate under the studied conditions.

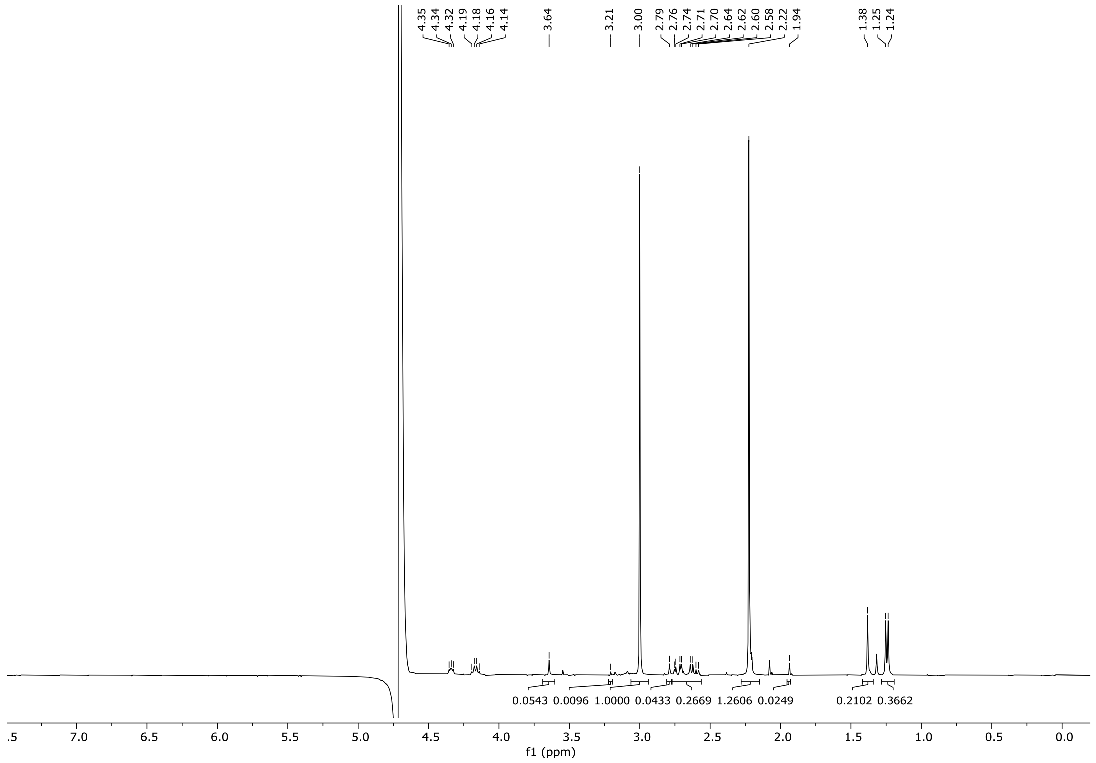

Figure S89. Representative ^{1}H qNMR of a reaction containing powder of the Campo del Cielo meteorite (Table S14 entry 1−2).

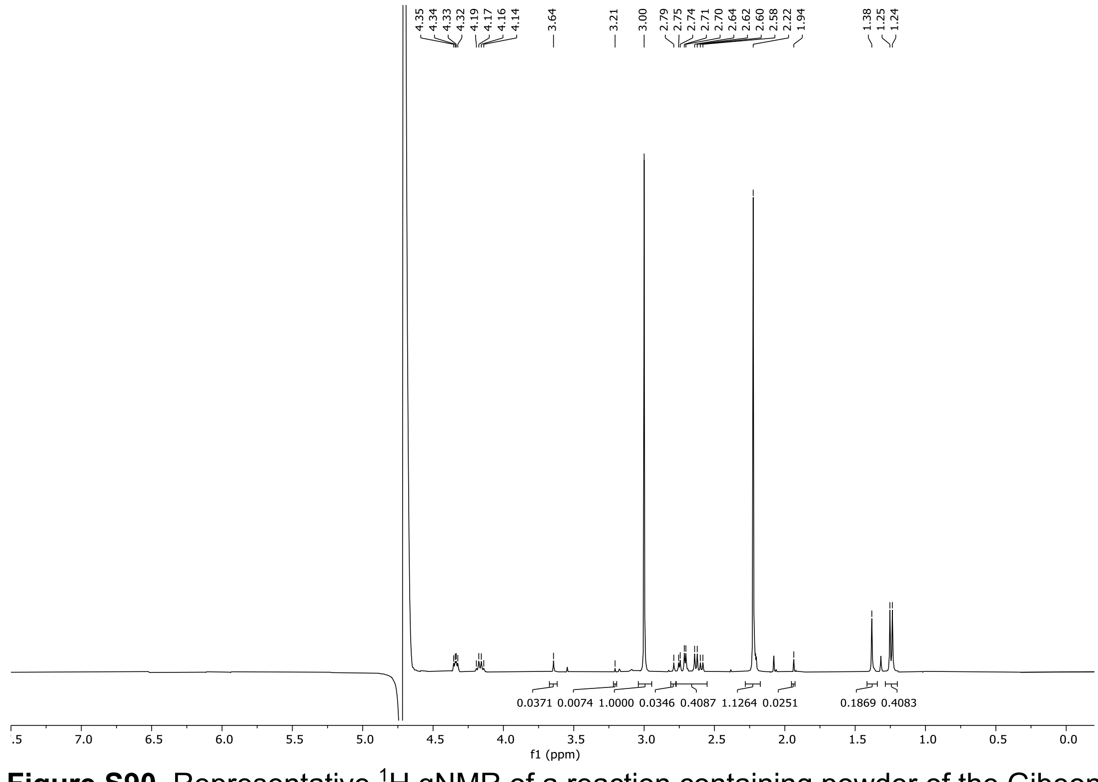

Figure S90. Representative ^{1}H qNMR of a reaction containing powder of the Gibeon meteorite (Table S14 entry 3−4).

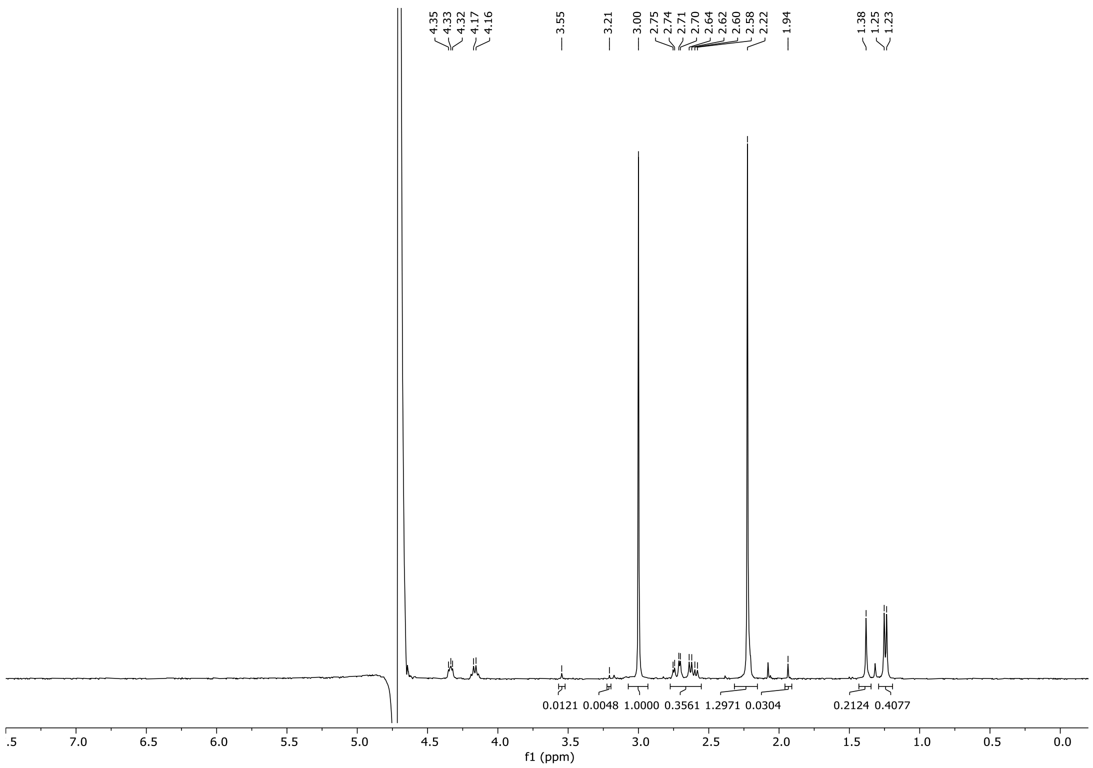

Figure S91. Representative ^{1}H qNMR of a reaction containing powder of the Sikhote Alin meteorite (Table S14 entry 5−6).

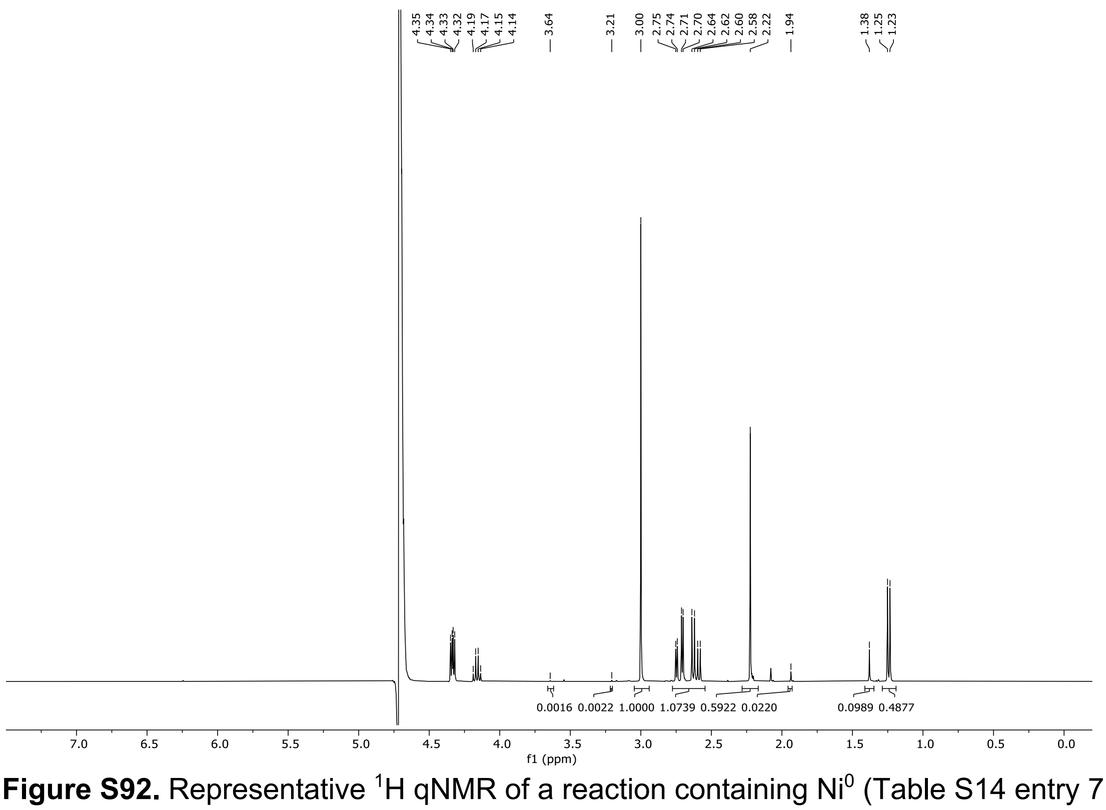

Figure S92. Representative ^{1}H qNMR of a reaction containing Ni^0 (Table S14 entry 7−8).

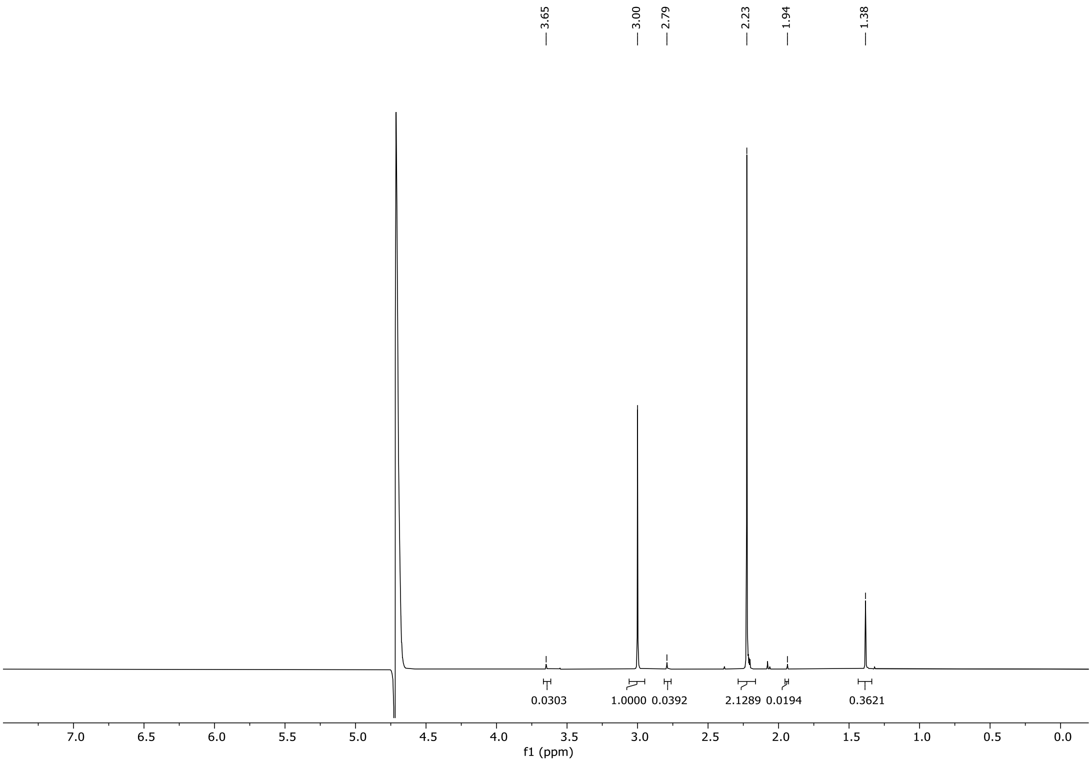

Figure S93. Representative ^{1}H qNMR without catalyst (Table S14 entry 9−10).

2.8.3 Meteorite screen for the reduction of oxaloacetate at 1 atm hydrogen

A disposable glass vial with PTFE/silicone-lined septum (see section 1.2 for more details) with meteorite powder (10 mg). Then, oxaloacetic acid (0.03 mmol, 3.96 mg, 1 equiv.) was added from an aqueous stock solution (0.06 M, pH 2) and water was added to a total volume of 1 mL solvent. After purging the vial for 3 min with H_2, the vial equipped with an H_2 balloon was stirred at 22 °C for 18 h. Experiments were done in duplicates. qNMRs were acquired according to section 1.4.2. Mean values of yields and standard errors were calculated for each set of reactions.

Table S15. Reaction conditions: oxaloacetic acid (30 mM), Gibeon meteorite (10 mg), H_2 (1 atm, balloon), pH 2, 22 °C, 18 h.

			integrals relative to DMS (6H) set to 1.0000 at 3.0000 ppm						yield [%]					
**#**	**catalyst loading (equiv., mol%, mg)**	**conc. DMS [M]**	**oxalo-acetate (2H)**	**malate (2H)**	**succi-nate (4H)**	**pyruvate (3H)**	**lactate (3H)**	**acetate (3H)**	**oxalo-acetate**	**malate**	**succi-nate**	**pyruvate**	**lactate**	**acetate**
**1**[a]	Gibeon (10 mg)	0.01815	0.0161	0.2650	0.0023	0.6921	0.7924	0.0497	1.5	24.0	0.1	20.9	24.0	1.5
**2**[a]	Gibeon (10 mg)	0.01815	0.0233	0.4664	0.0019	07975	0.8946	0.1753	2.1	21.2	0.0	24.1	27.1	5.3
									1.8 ± 0.3	22.6 ± 1.4	0 ± 0	22.5 ± 1.6	25.5 ± 1.5	3.4 ± 1.9

[a] formate was observed in traces

Gibeon meteorite powder was not able to reduce oxaloacetate at pH 7 at this pressure.

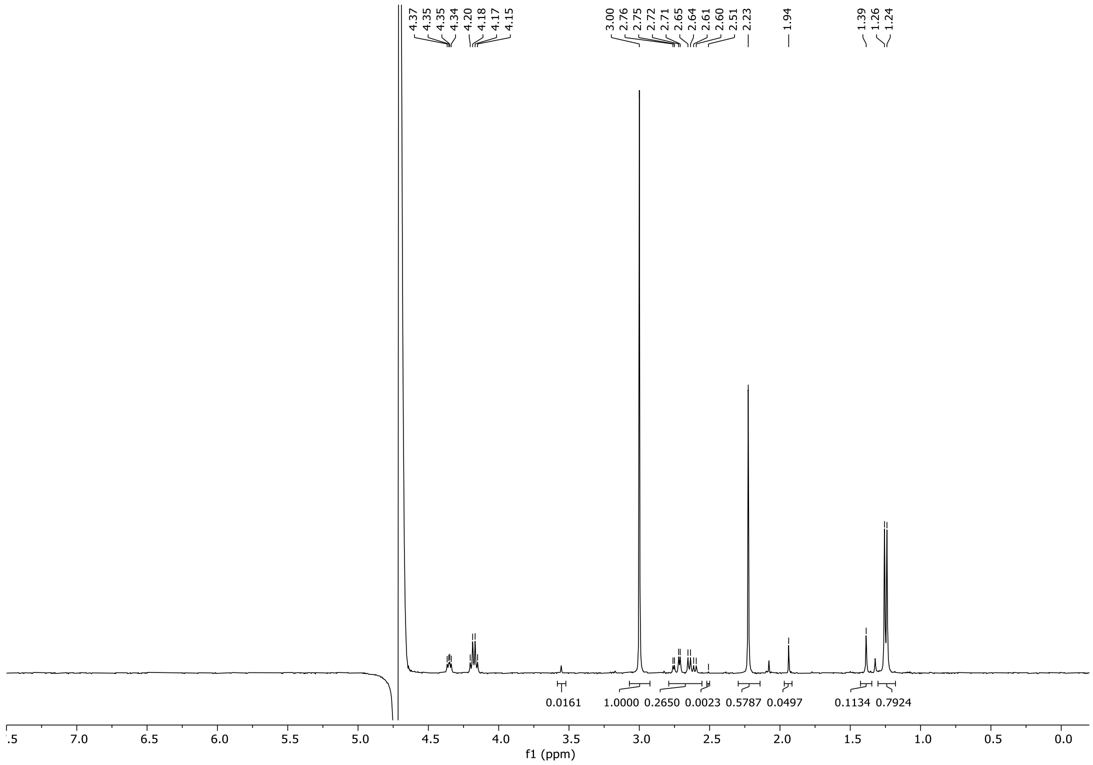

Figure S94. Representative ^{1}H qNMR of a reaction at pH 2 and 1 atm hydrogen containing powder of the Gibeon meteorite (Table S15).

2.8.4 Control reaction

2.8.4.1 Argon atmosphere

To investigate whether hydrogen is necessary as reducing agent, reactions under argon atmosphere were performed. Therefore, a disposable glass vial with PTFE/silicone-lined septum (see section 1.2 for more details) was charged with meteorite powder (10 mg or 0.5 mg) or metal powder (10 mg). Then, oxaloacetic acid (0.03 mmol, 3.96 mg, 1 equiv.) was added from an aqueous stock solution (0.06 M adjusted to pH 7−8 with NaOH) and water was added to a total volume of 1 mL solvent. After purging the vials for 3 min with argon, the vial was equipped with an argon balloon and was stirred at 22 °C for 18 h. qNMRs were acquired according to section 1.4.2.

Table S16. Reaction conditions: oxaloacetic acid (30 mM), meteorite powder **or** Ni^0 (10 mg), argon (1 atm), pH 7−8, 22 °C, 18 h.

			integrals relative to DMS (6H) set to 1.0000 at 3.0000 ppm						yield [%]					
**#**	**catalyst loading (mg)**	**conc. DMS [M]**	**oxalo-acetate (2H)**	**malate (2H)**	**succi-nate (4H)**	**pyruvate (3H)**	**lactate (3H)**	**acetate (3H)**	**oxalo-acetate**	**malate**	**succi-nate**	**pyruvate**	**lactate**	**acetate**
**1**	Campo del Cielo (10 mg)	0.01815	0	0	0	1.6812	0	0.1124	0	0	0	50.9	0	3.4
**2**	Gibeon (10 mg)	0.01815	0	0	0	2.1529	0	0.0831	0	0	0	65.1	0	2.5
**3**	Sikhote Alin (10 mg)	0.01815	0	0	0	1.9880	0	0.0909	0	0	0	60.1	0	2.7
**4**	Ni^0 (10 mg)	0.01815	0	0	0	2.4508	0	0.0583	0	0	0	74.1	0	1.8

The NMR samples contained small amounts of starting material and acetate, and mainly the decarboxylation product pyruvate. No reduction to malate or lactate was observed. From this it can be concluded that iron or nickel from the meteorite cannot reduce oxaloacetate by itself, but hydrogen is needed as a reducing agent.

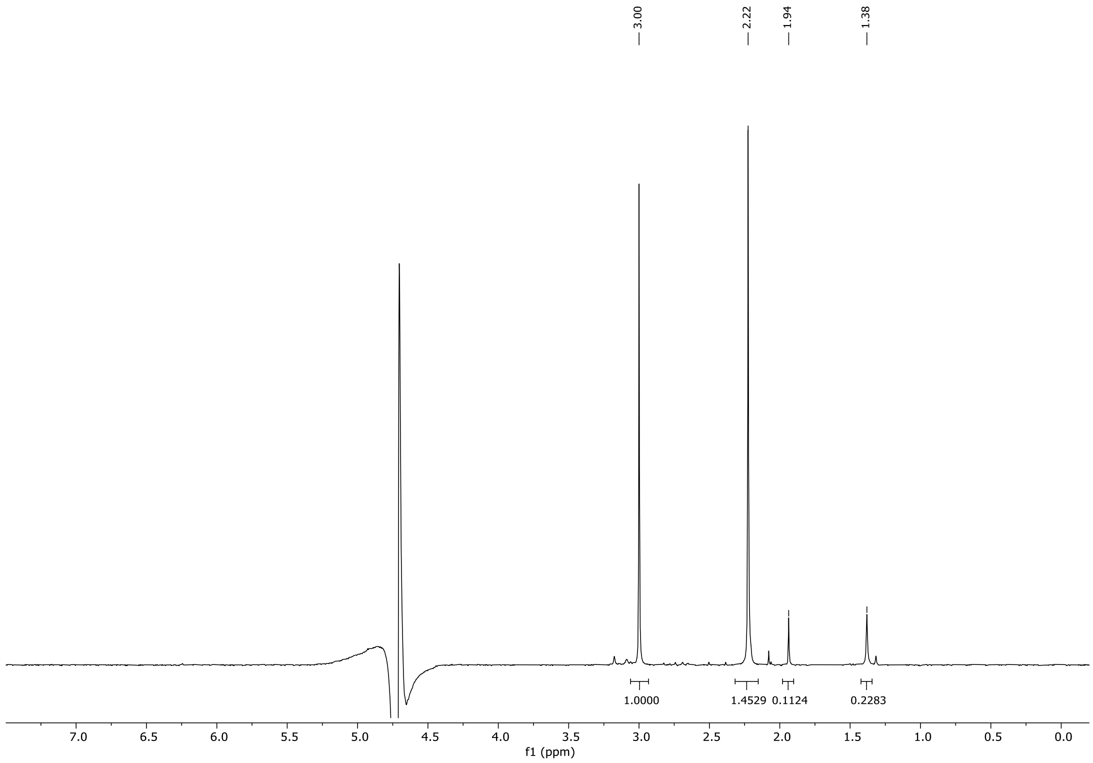

Figure S95. ^{1}H qNMR of a reaction under argon containing powder of the Campo del Cielo meteorite (Table S16 entry 1).

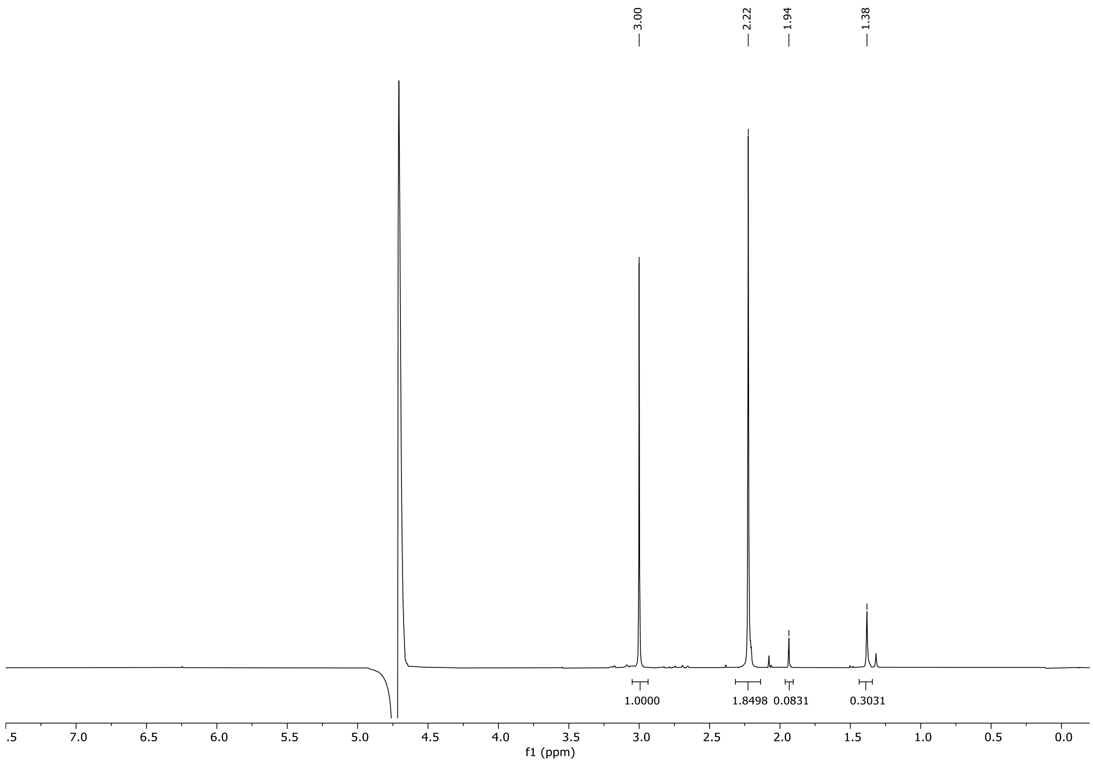

Figure S96. ^{1}H qNMR of a reaction under argon containing powder of the Gibeon meteorite (Table S16 entry 2).

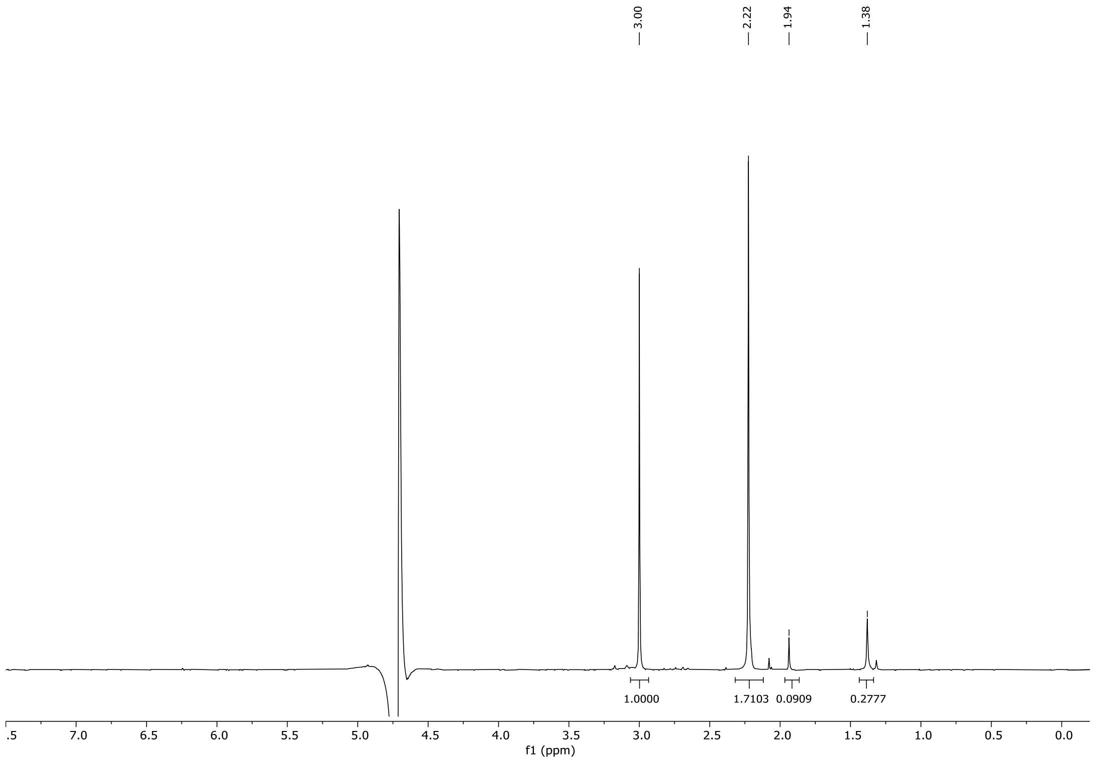

Figure S97. ^{1}H qNMR of a reaction under argon containing powder of the Sikhote Alin meteorite (Table S16 entry 3).

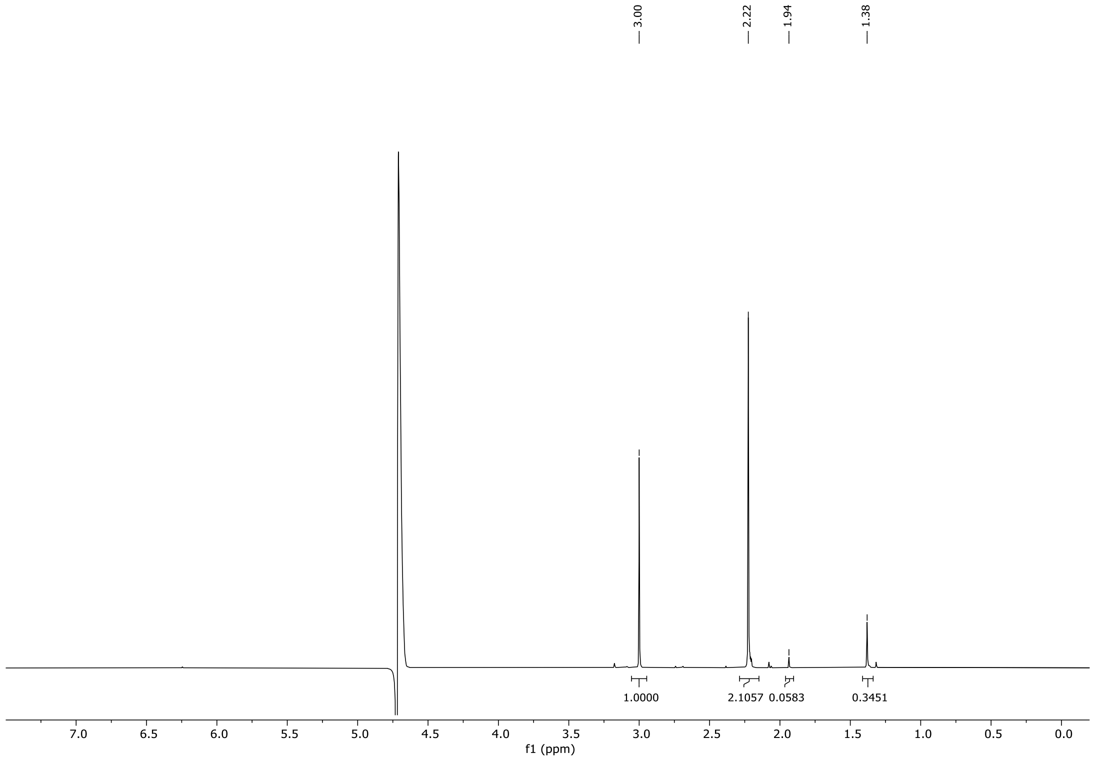

Figure S98. ^{1}H qNMR of a reaction under argon containing Ni^{0} powder (Table S16 entry 4).

2.8.4.2 Leakage of organic substances

To exclude the leakage of organic matter out of the meteorite, 10 mg of each meteorite powder was added to a vial containing 1 mL of MQ-H_2O. The solution was stirred for 18 H at 22 °C. qNMRs were acquired according to section 1.4.2.

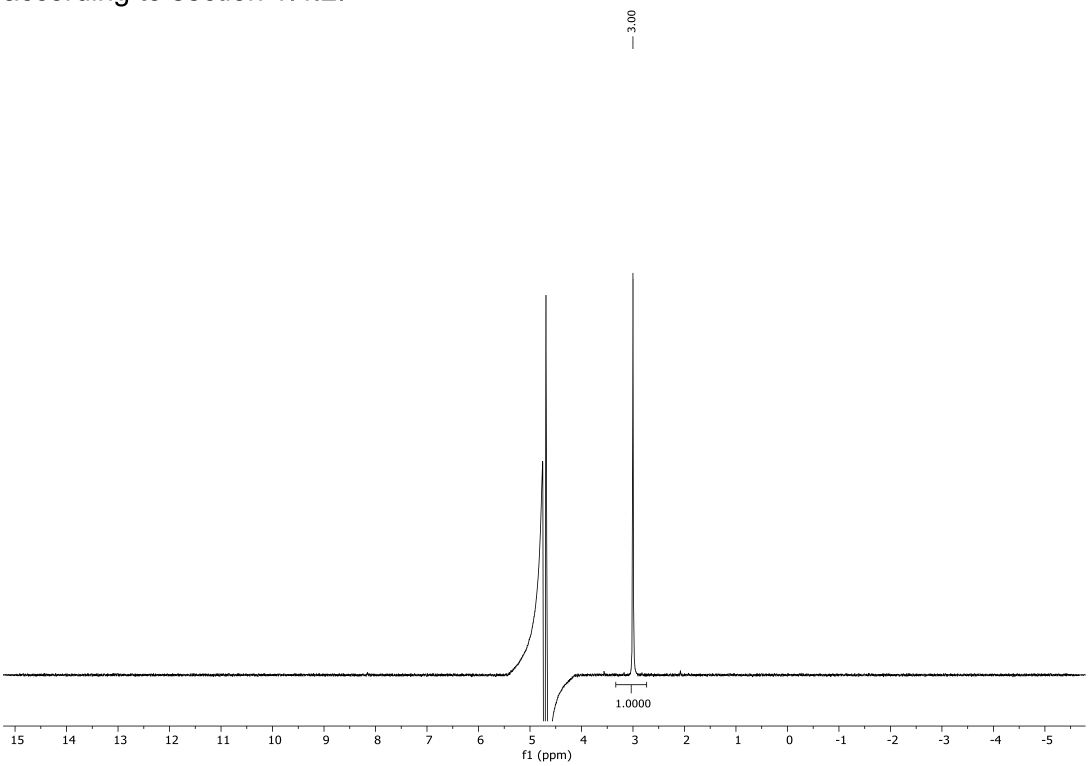

Figure S99. ^{1}H qNMR of the supernatant after stirring Campo del Cielo meteorite powder in water.

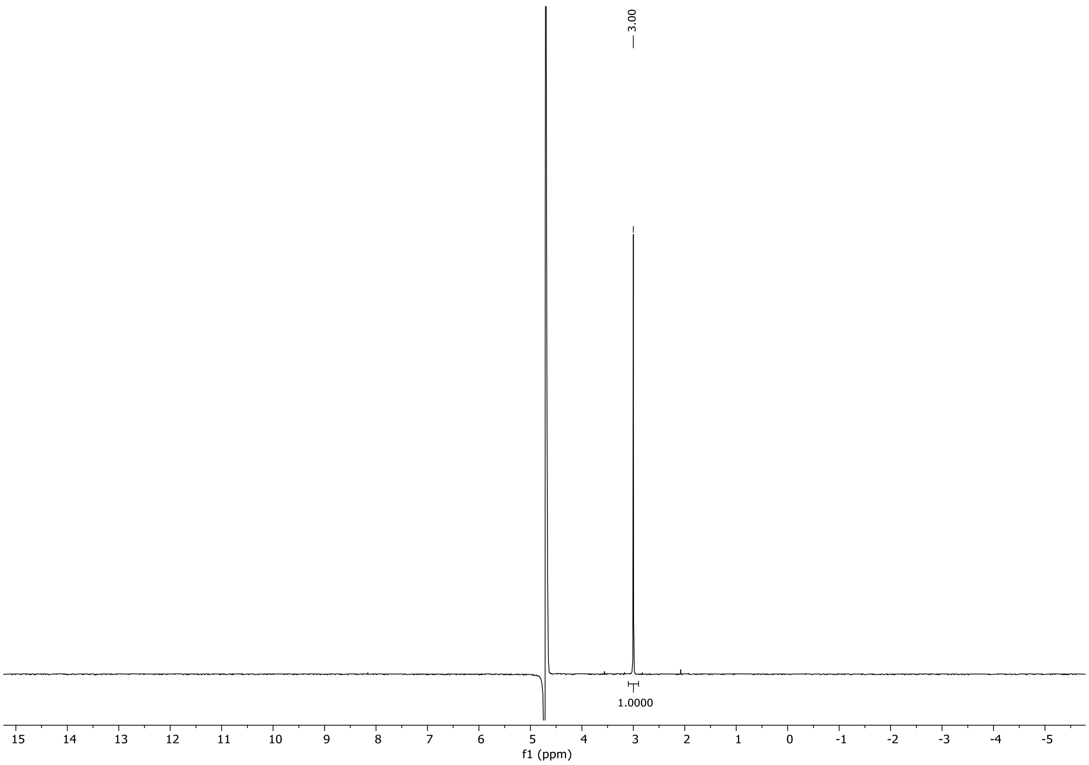

Figure S100. ^{1}H qNMR of the supernatant after stirring Gibeon meteorite powder in water.

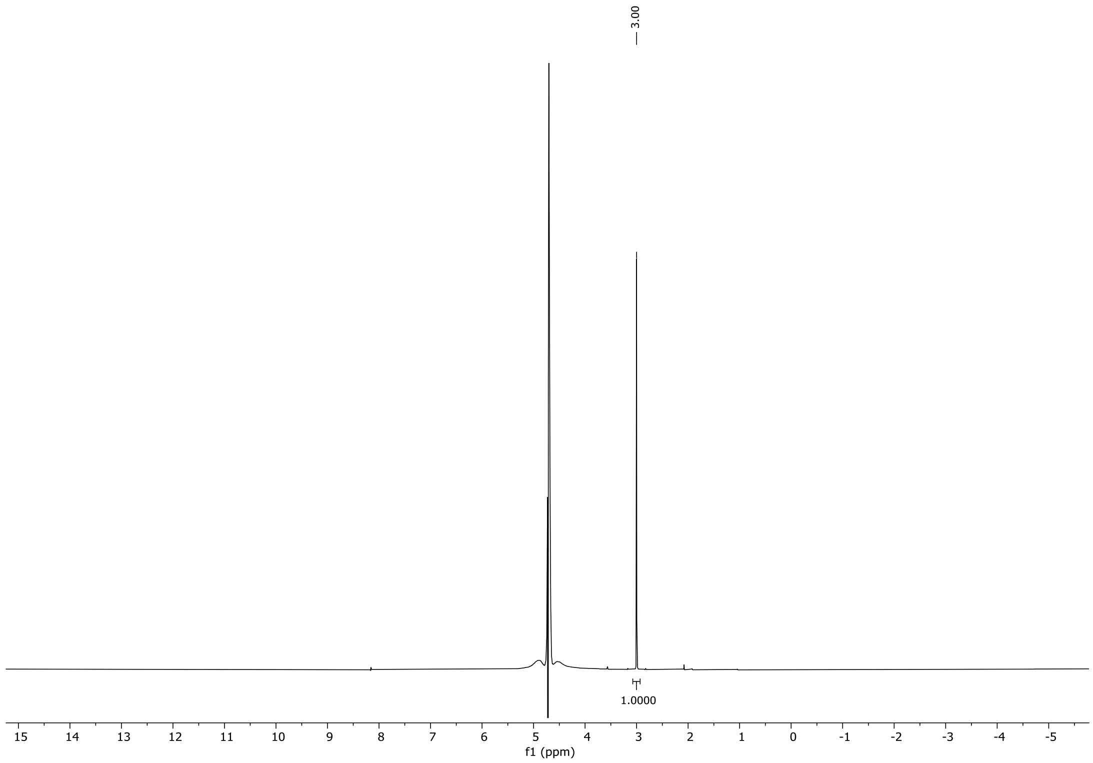

Figure S101. ^{1}H qNMR of the supernatant after stirring Sikhote Alin meteorite powder in water.

3 References

[1] D. E. Ryan, J. Holzbecher, R. R. Brooks, *Chem. Geol.* **1990**, *85*, 295–303.

[2] V. F. Buchwald, in *Handb. Iron Meteorites Vol. 2*, University Of California Press, Berkeley, **1975**, pp. 372–381.

[3] V. F. Buchwald, in *Handb. Iron Meteorites Vol. 2*, University Of California Press, Berkeley, **1975**, pp. 583–601.

[4] V. F. Buchwald, in *Handb. Iron Meteorites Vol. 2*, University Of California Press, Berkeley, **1975**, pp. 1119–1134.

[5] A. N. Krot, K. Keil, E. R. D. Scott, C. A. Goodrich, M. K. Weisberg, *Treatise Geochemistry Second Ed.* **2013**, *1*, 1–63.
